# Optogenetic stimulation of mouse Hoxb8 microglia in specific regions of the brain induces anxiety, grooming, or both

**DOI:** 10.1038/s41380-023-02019-w

**Published:** 2023-04-10

**Authors:** Naveen Nagarajan, Mario R. Capecchi

**Affiliations:** Department of Human Genetics, University of Utah School of Medicine, Salt Lake City, Utah USA

**Keywords:** Neuroscience, Psychiatric disorders

## Abstract

Previously, we have shown that either disruption of the *Hoxb8* gene or ablation of a microglial subpopulation, *Hoxb8* microglia, results in mice exhibiting both chronic anxiety and OCSD-like behavior, compulsive pathological hair pulling (trichotillomania), to the point of showing lesions at the sites of overgrooming. Herein we show, that optogenetic stimulation of *Hoxb8* microglia in specific regions of the brain induces elevated anxiety, grooming or both. Optogenetic stimulation of *Hoxb8* microglia within the dorsomedial striatum (DMS) or the medial prefrontal cortex (mPFC) induces grooming, whereas stimulation of *Hoxb8* microglia in the basolateral amygdala (BLA) or central amygdala (CeA) produces elevated anxiety. Optogenetic stimulation of *Hoxb8* microglia in the ventral CA1 region of the hippocampus (vCA1) induces both behaviors as well as freezing. In vitro we directly demonstrate that optogenetic stimulation of *Hoxb8* microglia in specific regions of the brain activate neighboring neural activity through the induction of the c-fos-immediate early response. These experiments connect outputs from optogenetically stimulated *Hoxb8* microglia, within specific regions of the brain, to the activation of neurons and neural circuits that in turn enable induction of these behaviors. These experiments suggest that *Hoxb8* microglia are likely to be among, or the main, first responders to signals that evoke these behaviors. The same regions of the brain (DMS, mPFC, BLA, CeA and vCA1) have previously been defined at the neuronal level, by optogenetics, to control anxiety in mice. Intriguingly, the optogenetic experiments in microglia suggest that the two populations of microglia, canonical non-*Hoxb8* and *Hoxb8* microglia, function in opposition rather than in parallel to each other, providing a biological reason for the presence of two microglial subpopulations in mice.

## Introduction

Chronic anxiety is an enormous world health problem. In the US, the National Institute of Mental Health reports that the lifetime occurrence of chronic anxiety is greater than 20% among adults, with higher prevalence in women (23.4%) than in men (14.8%). These statistics are even more disturbing for adolescents, with 38% of females and 26% of males reporting disabling disease. Yet our molecular genetic understanding of chronic anxiety and its relation to stress has not dramatically advanced since delineation of the Hypothalamic–Pituitary–Adrenal axis several decades ago [1, 2].

We have generated a mouse line in which the disruption of a single gene, *Hoxb8*, results in both chronic anxiety and OCSD-like behavior, pathological overgrooming, to the point of lesion at the sites of overgrooming (trichotillomania-like behavior, a subset of OCSD) [3–6]. As in humans, our mouse model exhibits strong female sex bias for both disorders [6]. The more excessive grooming and elevated anxiety observed in female *Hoxb8* mutant mice can be reduced to male levels by the elimination of female sex hormone production either by removal of the ovaries or by treatment with trilostane which inhibits female sex hormone synthesis [6]. Conversely, *Hoxb8* mutant males supplemented by progesterone and β-estradiol show increased female levels of pathological grooming and anxiety [6]. Further, we have demonstrated by cell transplantation of purified, cell-sorted *Hoxb8* progenitor cells derived from *Hoxb8* mutant mice, but not progenitor cells derived from wild-type *Hoxb8* mice, into wild-type recipient mice devoid of microglia, that defective *Hoxb8* microglia are causative for both behavioral pathologies (6 and unpublished results).

*Hoxb8* is a transcription factor. *Hox* genes are key players during development in all multicellular organisms, their primary role being the specification of body plans. Unexpectedly, *Hoxb8* mouse mutants do not exhibit changes in body morphology, but rather changes in behavior [3]. However, *Hox* genes are also involved in establishing hematopoiesis and *Hoxb8* is used to specify the myeloid lineage during definitive hematopoiesis [7]. Microglia, the macrophages of the brain, are derived from myeloid progenitors [8]. In this context, expression of *Hoxb8* leads to the genesis of a previously unidentified subpopulation of microglia, *Hoxb8* microglia [4–7].

As stated above, microglia are the resident immune cells of the central nervous system (CNS). Based on elegant cell lineage tracing by F. Ginhoux et al., it was believed that there was a single source of microglia, the yolk sac, starting at gestation day 7.5 followed by migration of the microglia progenitor cells into the developing brain at E9.5 [8]. By their own admission, *Runx1*, their cell lineage marker, could only account for ~60% of adult microglia, leaving room for additional microglial cell lineages [8]. Subsequently, our laboratory demonstrated that there are at least two progenitor pools for microglia: canonical non-*Hoxb8* microglia [8] and *Hoxb8* microglia, that are also born in the yolk sac but a day later (E8.5) and then migrate to the AGM (Aorta Gonad Mesonephros region) and fetal liver, where they are amplified 16 and 280-fold respectively, prior to their entry into the brain starting at E12.5 [5, 7]. The two microglia populations can be further distinguished because only *Hoxb8* progenitors are dependent on *cMyb* for survival [7, 8]. Though non-*Hoxb8* microglia comprise ~70% of total microglia in the adult brain, they cannot compensate for the loss of *Hoxb8* microglia function (~30% of microglia in the adult mouse brains) [5, 6], arguing for separation of functions between these two subpopulations of microglia.

Mouse models have implicated corticostriatal thalamic circuitry [9, 10], the amygdala [11–16] and the ventral CA1 region of the hippocampus (vCA1 [17]) as critical brain regions controlling levels of anxiety and OCSD-like behaviors [18]. Herein, we will show that optogenetic activation of *Hoxb8* microglia in the same defined regions of the brain induce anxiety, grooming or both. What do we mean by optogenetic activation of *Hoxb8* microglia? Microglia are not neurons and do not generate action potentials [19, 20]. We mean that optogenetic stimulation of *Hoxb8* microglia causes membrane depolarization resulting from light induced influx of cations, as was demonstrated in microglia residing in the spinal cord by Yi et al. [21] and herein for microglia in the brain. We have found that the behavioral outputs are exquisitely sensitive to optogenetic stimulation of *Hoxb8* microglia, but work within time frames of multiple seconds rather than milliseconds, a longer time frame possibly required for example, to secrete ligands that could stimulate neighboring neurons or to signal the microglial membrane depolarization in some other way to neuronal counter parts.

It should be stated, that by optogenetic stimulation of *Hoxb8* microglia in specific regions of the brain, we are not inducing pathological levels of grooming and/or anxiety. These results should not be confused with the consequences of disruption of the *Hoxb8* gene [3–6]. Optogenetic activation of *Hoxb8* microglia in specific regions of the brain induces higher levels of anxiety, grooming or both, but not pathological levels of anxiety or grooming. At appropriate levels both anxiety and grooming are beneficial. The optogenetic inducible behaviors are readily reversible, stop when the laser is turned off and show no sex bias. In contrast, *Hoxb8* mutant mice show chronic, pathological manifestations of both behaviors that are not reversible and exhibit female sex bias. It is the pathological manifestation of the behaviors that appear to exhibit sex bias.

To summarize, herein we demonstrate that two well defined behaviors in mice, anxiety and grooming, can be specifically induced by optogenetic stimulation of *Hoxb8* microglia in specific regions of the brain. Optogenetic stimulation of *Hoxb8* microglia in the DMS and mPFC induces grooming, whereas optogenetic activation in the BLA and CeA induces anxiety. Opotogenetic stimulation of *Hoxb8* microglia in the vCA1 hippocampus induces both behaviors as well as freezing. Surprisingly, co-optogenetic activation of both microglial subpopulations in these same regions of the brain result in canceling the induction of grooming and anxiety behaviors. Taken together, the two sets of results strongly suggest that the two populations of microglia function in opposition to each other, thus providing a biological reason for the existence of the two subpopulations of microglia in mice.

## Material and methods

All experiments were approved by the Institutional Animal Care and Use Committee (IACUC), University of Utah.

### Mice

*Gt(ROSA)26Sor*^*tm14(CAT-tdTomato)Hze*^ (Ai14, #007908) and *Gt(ROSA)26Sor*^*tm32*^^(CAG-COP4*H134R/EYFP)Hze^ conditional allele (Ai32, #012569) were obtained from the Jackson Laboratory. *Cx3cr1*^*GFP*^ mice (#005582) mice were a kind gift from Dr. Monica Vetter, Department of Neuroanatomy and Neurobiology, University of Utah. *Hoxb8*^*IRES-Cre*^ was generated in our laboratory and reported in Chen et al. [4], *Iba1*^*IRES-Cre*^ was also generated in our laboratory and reported in Pozner et al. [22]. *GFAP-Cre* mice were obtained from the Jackson Laboratory (strain # 012886). To avoid germline recombination, homozygous *GFAP-Cre* males were bred to homozygous floxed females. Note that YFP, which is also under Cre control in the Ai32 allele, is only expressed in astrocytes (Supplementary Fig. 24j), thus germline recombination is not being observed in these crosses. 3–4 month-old mice were used for implantation. All test mice were conditioned for 5–15 min in specific experimental rooms prior to the experiments. All behavioral experiments were conducted during day light period of the light/dark cycle.

A uniform genetic background is critical for obtaining reproducible mouse behavioral studies. For this reason, every mouse strain that we have used for these experiments was extensively backcrossed (i.e., breeding for at least seven generations to the same C57Bl/6 mouse strain obtained from the Bar Harbor Jax Laboratories), allowing us to use C57Bl/6 mice as controls, among others.

### Surgery, implantations, and housing

All survival surgeries were performed under aseptic conditions under stereotaxic equipment (Kopf instruments). Mice were anaesthetized using 4.0% isoflurane during induction and maintained at 1.5% throughout the surgical procedure. All surgically implanted mice were housed in individual cages till the end of the experiment. All stereotactic coordinates are in relation to bregma in mm. All mice received unilateral implantation of cannula (PlasticsOne, Roanoke, VA) for the brain regions DMS, dmPFC, BLA, CeA and vCA1. A beveled cannula was used to target CeA. Cannulas were implanted at the following stereotaxic coordinate: DMS (+0.74 AP, +2.0 ML, −2.2 DV), mPFC (+1.9 AP, 0.4 ML, −2.0 DV), BLA (−1.6 mm AP, ±3.1 mm ML, −4.5 mm DV), CeA (−1.06 mm AP, ±2.25 mm ML, −4.4 mm DV), vCA1 (−3.16 mm AP, +3.50 ML, −3.5 DV).

### Optogenetic stimulation

For optical stimulation common to all behavioral experiments, multimode optical fiber (NA 0.37; 200 μm core; Thorlabs, Newton, NJ) was connected to a 473 nm light source through an FC/PC adapter. The free end of the fiber was connected to the implanted cannula before the initiation of the experiment. Following experiment, the optic fiber was gently removed and a dust cap was secured on the cannula. The mice were kept back in the home cage to recover from the optogenetic stimulation.

### Behavioral experiments

#### Grooming behavior

All grooming behaviors in the experiments were measured by 6 min of video recordings with 2 min of each baseline, optogenetic stimulation and post stimulation condition within the home cage. The home cage environment was chosen for recording in order to ensure that other environmental factors do not affect the experimental outcome. Each experimental subject was pre-conditioned with optic fiber for 5 min before the experiment started. Laser power for the experimental and control subjects ranged between 6.6 and 8.5 mW for DMS and mPFC brain regions. The same laser powers were used to test control versus experimental subjects for the behavioral output. The laser power reported represents the power emerging from the laser light source. After each recording session, the optic fiber was carefully removed until the next experimental session. From the recorded videos, the behavioral phases of grooming were classified into phase III and phase IV if the experimental subject displayed facial grooming (phase III) or body grooming (phase IV). Each individual grooming bout corresponding to phase III and phase IV was scored for every experiment performed within the pre, during and post stimulation conditions for each experimental and control subject for every genotype tested. The latency or the onset of grooming was measured based on the first occurrence of phase III or phase IV grooming bout in response to optogenetic stimulation.

### Freezing behavior

Freezing behaviors were measured manually by behavioral scoring of 6 min of video recordings. The pre-stimulation and post-stimulation epochs were 2 min each. All experiments were conducted within the home cage. Each experimental subject was pre-conditioned with optic fiber for 5 min before the experiment started. The optogenetics protocol consisted of 2 min baseline pre-stimulation, 2 minutes of blue light stimulation and 2 min of post stimulation recording. Photostimulation consisted of continuous light stimulation (473 nm) at 2.3 mW within vCA1 region of the hippocampus in order to induce freezing behavior. Other laser power that were tested for freezing behavior in experimental and control mice ranged between 1.7 and 2.8 mW. The laser power reported represents the power emerging from the laser light source. After each recording session, the optic fiber was carefully removed until the next experimental session. From the recorded videos, the behavioral phases of freezing were scored based on the transition time when the subject shifted from normal to freezing behavior. The time onset of freezing and length of freezing in response to optogenetic stimulation was quantified for each experimental subject tested.

### Elevated plus maze test

The elevated plus maze platform (dimension, 5 × 35 × 15 × 40 cm) that was used to assess anxiety-like behavior in the experiments exploited the conflict between the innate fear that mice exhibit in the open areas versus their preferred enclosed areas in the platform. The arms of the platform were non-reflective base plates that were gray in color. While the open arm had no walls, the closed arm walls were held solidly in slotted base. Mouse movement with and without stimulation was tracked using ANY-Maze software (Stoelting, Wood Dale, IL, USA). The optogenetics protocol consisted of 10 min baseline pre-stimulation, 5 min of blue light stimulation and 10 min of post stimulation recording. Photostimulation consisted of continuous light stimulation (473 nm) at 11.4 mW within BLA and CeA in order to induce anxiety-like behaviors. The laser power reported represents the power emerging from the laser light source. Following every experiment, the closed arms were lifted off and the platform was thoroughly cleaned with 70% ethanol and dried before every experiment.

### Open field test

The open-field (dimension, 40 × 40 × 35 cm) arena was used to assess anxiety-like behavior in the experiments. The subjects were tested to determine the preference for the center zone versus periphery zone. Mouse movement into center and periphery zone with and without optogenetic stimulation was tracked using ANY-Maze software (Stoelting, Wood Dale, IL, USA). The optogenetics protocol consisted of 6 min baseline pre-stimulation, 6 min of blue light stimulation and 6 min of post stimulation recording. Photostimulation consisted of continuous blue light stimulation (473 nm) at 11.5 mW within BLA and CeA in order to induce anxiety-like behaviors. The laser power reported represents the power emerging from the laser light source. Following every experiment, the open field arena was thoroughly cleaned with 70% ethanol and dried before every experiment.

### Slice electrophysiology and whole slice optogenetic stimulation

*HoxB8*^*IRESCre*^*/Ai32 or* C57/Bl6 mice were deeply anesthetized with isoflurane (4%). Brains were placed in ice-cold (4 °C) oxygenated Sucrose-based Artificial Cerebral Spinal Fluid (ACSF) solution (95% O_2_/5% CO_2_) containing (in mM): Sucrose (180.0), KCl (3.0), Na_2_PO_4_ (1.4), MgSO_4_ (3.0), NaHCO_3_ (26.0), glucose (10.0), and CaCl_2_ (0.5). Oblique slices (350 µm) preserving connections between frontal/orbital cortex and striatum were made and transferred to an incubation chamber containing oxygenated ACSF at room temperature for 2 h prior to recording. ACSF contained (in mM): NaCl (126.0), KCl (3.0), Na_2_PO_4_ (1.4), MgSO_4_ (1.0), NaHCO_3_ (26.0), glucose (10.0), and CaCl_2_ (2.5). The pH (7.30–7.40) and osmolarity (290–300 mOsm) of the ACSF and sucrose-ACSF were verified prior to each experiment. Cell attached recordings were performed in all the experiments. For whole slice optogenetic stimulation, individual slices were exposed to various stimulation frequencies of blue light or continuous blue light (470 nm) stimulation driven by LED within DG4 connected to a Mosaic system attached to the Olympus BX51WI microscope.

### Data analysis and statistics

Video recording data were manually analyzed to detect phase III and IV type of grooming behaviors. All data recorded from Anymaze were analyzed and statistical comparison was made using Prism (GraphPad) software. All electrophysiological data were analyzed using clampfit software package (Molecular Devices). Spike analysis to characterize spike frequency, instantaneous frequency, inter-event interval, half width, rise and decay time, time to peak and amplitude was performed after setting the threshold manually on individual recorded files. The same threshold was applied to all traces analyzed. Two-tailed unpaired *t* test with Welch’s correction, two-tailed paired *t* test, repeated measure One-way ANOVA with Geisser greenhouse correction followed by Tukey’s HSD posthoc multiple comparison tests, One-way ANOVA with Tukey’s HSD posthoc test and Kruskal-Wallis test followed by Dunn’s multiple comparison test, were used for the data comparison as described in the figures. In case of non-normally distributed data sets, nonparametric tests were used and are reported as appropriate. The data were considered not significant if *P* > 0.05 and significant if *P* < 0.05 (*), *P* < 0.01 (**) and *P* < 0.001 (***).

### Immunohistochemistry and confocal imaging

Mice were anesthetized and transcardially perfused with 4% PFA in PBS (pH 7.6). The brains were dissected, post-fixed for 2 h at room temperature, and processed through 10% sucrose in PBS overnight followed by 20% and 30% sucrose in PBS until they sink. These cryoprotected brains were embedded in 2% gelatin (Sigma G2500) and 0.9% NaCl in a metal mold, quickly frozen on a metal block cooled with liquid nitrogen, sectioned with Leica CM1900 at 20 µm thickness, and mounted on SuperFrost Gold Plus microscopy slides (Fisher Scientific). The sections were incubated with primary antibodies, chicken anti-GFP (1:500, Aves GFP-1020), rabbit anti-RFP (1:500, Rockland 600–401–379), anti-mouse NeuN (1:50, MAB377B, Sigma Aldrich), anti IBA1 (1:500, 019–19741, Wako), anti-mouse Kv1.3 K + channel (1:1000, Addgene), anti-rabbit c-Fos (1:1500, Cell Signaling Technology) diluted in Cyto-Q Immuno diluent and block (Innovex Biosciences). The secondary antibodies were anti-chicken Alexa Fluor 488 (1:500, A-11039, Thermo Fisher Scientific), goat anti-rabbit Alexa Fluor 555 (1:500, A-21428, Thermo Fisher Scientific), and anti-mouse Alexa Fluor 647 conjugated to streptavidin (S-21374, Thermo Fischer Scientific). All the antibodies used were monitored for its specificity and the cross-reactivity. The slides were stained with DAPI and mounted with Fluoromount-G (Southern Biotech), and image acquisition was performed with a Leica TCS SP5 confocal microscope. All images were collected using either 10× or 20× air objective. High-resolution images of microglia were acquired using 63× oil objective. The images were processed and analyzed using Imaris 7.7 (Bitplane).

## Results

### Induction of grooming by optogenetic activation of *Hoxb8* microglia in the DMS and mPFC

**Fig. 1:**
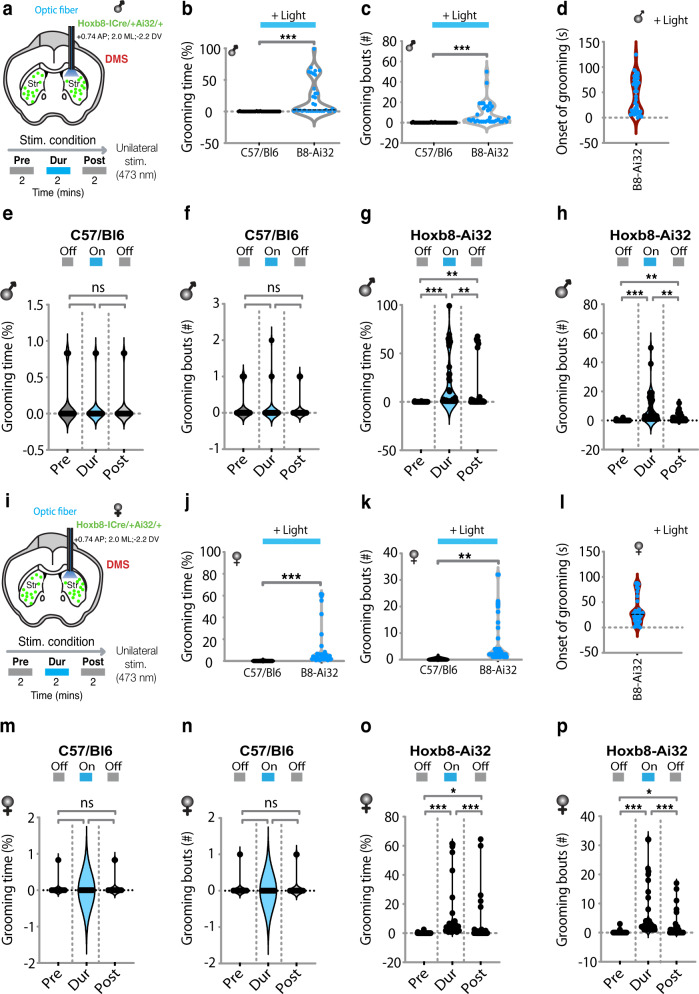
Optogenetic stimulation of Hoxb8 microglia within dorsomedial striatum (DMS) induces grooming behaviors. **a** Representation of cannula insertion and light (473 nm) delivery into DMS to stimulate *Hoxb8* microglia unilaterally in *Hoxb8*-^IRES-Cre/+^; Ai32^/+^ male (**a**) and female mice (**i**) or non-channelrhodopsin expressing C57/Bl6 control mice. Shown in the picture (**a**, **i**) is a straight cannula that was stereotaxically inserted within DMS in the right brain hemisphere at the coordinates +0.74AP, ±2.0 ML, 2.2DV. Stimulation condition consisted of a two-minute baseline recording, followed by 2 min of continuous optogenetic stimulation at 473 nm and 2 min of post stimulation recording. **b**, **c** Significant increase in the percentage of grooming time and the number of grooming bouts compared to C57/Bl6 control male mice in response to 2 min continuous optogenetic stimulation. **d** Representation of the onset of grooming (latency to groom) in response to 473 nm photostimulation that is restricted to *Hoxb8*-^IRES-Cre/+^; Ai32^/+^ male mice but not to C57/Bl6 control male mice. **e**–**h** Representation of pre, during and post stimulation condition showing a significant increase in the percentage of grooming time and the number of grooming bouts in *Hoxb8*-^IRES-Cre/+^; Ai32^/+^ (**g**, **h**) but not in C57/Bl6 control (**e**, **f**) male mice. (**j**, **k**). Significant increase in the percentage of grooming time and the number of grooming bouts in *Hoxb8*-^IRES-Cre/+^; Ai32^/+^ female mice compared to C57/Bl6 controls in response to 2 min continuous optogenetic stimulation condition. **l** Representation of the onset of grooming (latency to groom) in response to 473 nm photostimulation that is restricted to *Hoxb8*-^IRES-Cre/+^; Ai32^/+^ female mice but not to C57/Bl6 control female mice. **m**–**p** Representation of pre, during and post stimulation conditions showing a significant increase in the percentage of grooming time and the number of grooming bouts in *Hoxb8*-^IRES-Cre/+^; Ai32^/+^ (**o**, **p**), but not in C57/Bl6 control (**m**, **n**) female mice. Green dots within the stimulated area in Fig. 1a, i represents *Hoxb8* microglia expressing channelrhodopsin. Visual comparisons of the male and female data sets indicate great similarity between the grooming responses in the DMS to optogenetic stimulation of *Hoxb8* microglia. To further interrogate this question, we statistically compared the number of grooming bouts and % of grooming time spent by C57/Bl6 and *Hoxb8*^*IRESCre*^*/Ai32* mice during stimulation for significant differences in their grooming responses between the sexes and none was found (C57/Bl6 stim-on *P* = 0.996; *Hoxb8*^*IRESCre*^*/Ai32* stim-on *P* = 0.466 for male versus female comparison., % grooming time C57/Bl6 stim-on *P* > 0.999; *Hoxb8*^*IRESCre*^*/Ai32* stim-on *P* = 0.103.) Individual data point represents the behavioral response of the subject to individual laser powers to which the subjects were tested in at least 2–3 independent replicates. Laser power for the subjects ranged between 6.6 and 8.5 mW. ns, not significant. *P < 0.05, **P < 0.01 ***P < 0.001. Two-tailed Mann-Whitney *U* test was used for **b**, **c**, **j**, **k**. Repeated measure One-way ANOVA with Geisser greenhouse correction and Tukey’s HSD posthoc multiple comparison tests were used for **e**, **f**, **m**, **n**. Kurskal-Wallis test followed by Dunn’s multiple comparison test for **g**, **h**, **o**, **p**. 3–4 months old mice were used for the DMS implantation. *n* = 6 mice per gender, per genotype.

**Fig. 2:**
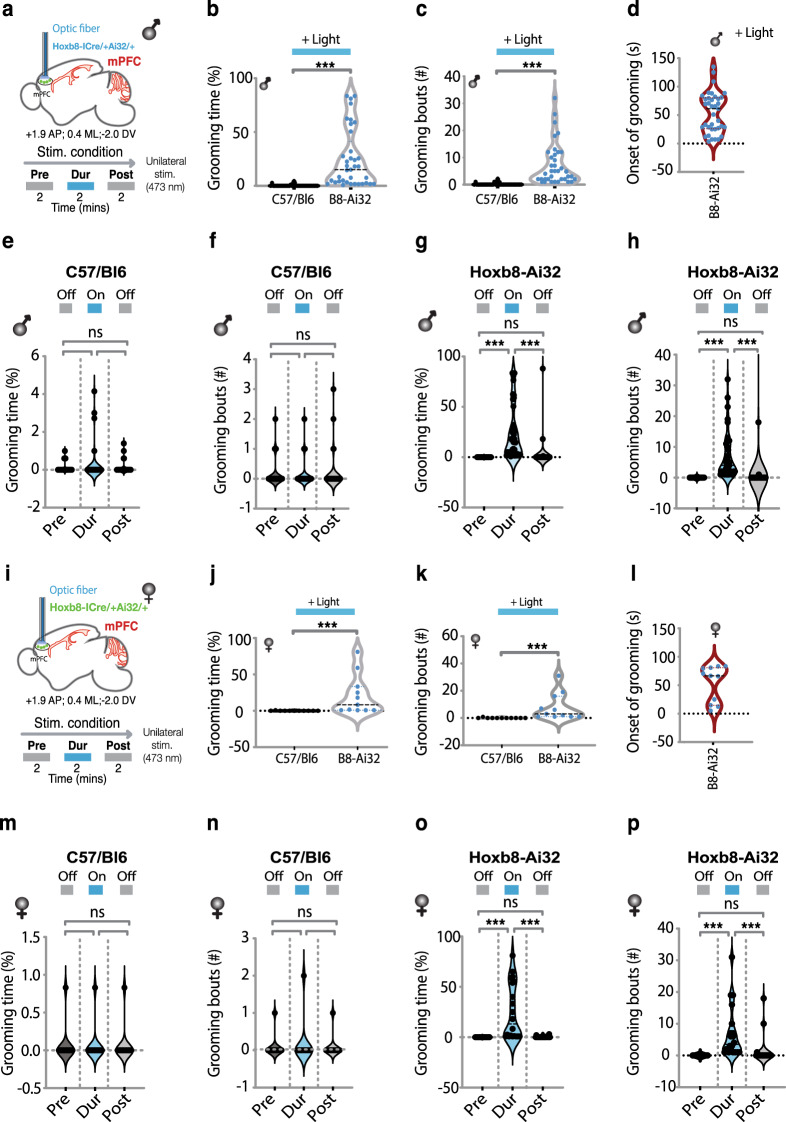
Optogenetic stimulation of Hoxb8 microglia within medial prefrontal cortex (mPFC) induces grooming behaviors. **a** Representation of cannula insertion and light (473 nm) delivery into mPFC to stimulate *Hoxb8* microglia unilaterally in *Hoxb8*-^IRES-Cre/+^;Ai32^/+^ male (**a**) and female (**i**) or non-channelrhodopsin expressing C57/Bl6 control mice. Shown in the picture (**a**, **i**) is a straight cannula that was stereotaxically inserted within mPFC in the right brain hemisphere at the coordinates +1.9 AP, 0.4 ML, −2.2 DV. Stimulation condition consisted of a 2-min baseline recording followed by 2 min of continuous optogenetic stimulation at 473 nm and 2 min of post stimulation recording. **b**, **c** Significant increase in the percentage of grooming time and the number of grooming bouts in *Hoxb8*-^IRES-Cre/+^;Ai32^/+^ male mice compared to C57/Bl6 controls in response to 2 min continuous optogenetic stimulation condition. **d** Representation of the onset of grooming (latency to groom) in response to 473 nm photostimulation that is restricted to *Hoxb8*-^IRES-Cre/+^; Ai32^/+^ male mice but not to C57/Bl6 control male mice. **e**–**h** Representation of pre, during and post stimulation condition showing a significant increase in the percentage of grooming time and the number of grooming bouts in *Hoxb8*-^IRES-Cre/+^;Ai32^/+^ (**g**, **h**) but not in C57/Bl6 control (**e**, **f**) male mice. (**j**, **k**). Significant increase in the percentage of grooming time and the number of grooming bouts in *Hoxb8*-^IRES-Cre/+^; Ai32^/+^ female mice compared to C57/Bl6 controls in response to 2-min continuous optogenetic stimulation condition. **l** Representation of the onset of grooming (latency to groom) in response to 473 nm photostimulation that is restricted to *Hoxb8*-^IRES-Cre/+^; Ai32^/+^ female mice but not to C57/Bl6 control female mice. **m**–**p** Representation of pre, during and post stimulation conditions showing a significant increase in the percentage of grooming time and the number of grooming bouts in *Hoxb8*-^IRES-Cre/+^;Ai32^/+^ (**o**, **p**), but not in C57/Bl6 control (**m**, **n**) female mice. Green dots within the stimulated area in Fig. 2a, i represents *Hoxb8* microglia expressing channelrhodopsin. Again, visual comparisons of the male and female data sets show that the *Hoxb8* microglia optogenetic induced grooming responses are very similar between the sexes, These data sets were also analyzed statistically to determine if any significant difference in the grooming response between the sexes in terms of grooming bouts or percent time spent grooming and none were found (C57/B6 stim-on *P* = 0.982; *Hoxb8*^*IRESCre*^*/Ai32* *P* = 0.965; % grooming time stim-on C57/Bl6 *P* = 0.984; *Hoxb8*^*IRESCre*^*/Ai32* *P* = 0.999). Individual data point represents the behavioral response of the subject to individual laser powers to which the subjects were tested in at least 2–3 independent replicates. Laser power for the subjects ranged between 6.6 and 8.5 mW. ns, *P* > 0.05, not significant. * *P* < 0.05, ** *P* < 0.01 *** *P* < 0.001. Two-tailed Mann-Whitney *U* test was used for **b**, **c**, **j**, **k**. Repeated measure One-way ANOVA with Geisser greenhouse correction and Tukey’s HSD posthoc multiple comparison tests were used for **e**, **f**, **m**, **n**. Kurskal-Wallis test followed by Dunn’s multiple comparison test for **g**, **h**, **o**, **p**. 3–4 months old mice were used for the mPFC implantation. *n* = 5 mice per gender, per genotype.

The components that are critical for achieving *Hoxb8* microglia dependent induction of anxiety and/or grooming in wildtype C57Bl/6 mice are: *Hoxb8* microglia, *Hoxb8*^*IRESCre*^ (a Cre driver specific to *Hoxb8* microglia within the CNS), *Ai32* (a Cre dependent light activatable channel-rhodopsin 2 and YFP as a fusion protein), the dorsomedial striatum (DMS), the basolateral amygdala (BLA), central amygdala (CeA) and ventral CA1 (vCA1) region of the hippocampus, the exposure to laser power (mW) and an adequate duration of light exposure (min). Thus, in *Hoxb8*^*IRESCre*^*/Ai32* mice, but not in C57Bl/6 control mice or Ai32/+ (mice lacking *Hoxb8*^*IRESCre*^), optogenetic stimulation within the DMS or mPFC for two minutes at laser powers of 6.6–8.5 mW results in the induction of robust grooming, which stops when the laser is turned off, with variable off kinetics (Figs. 1, 2; Supplementary Fig. 1). The protocol involves continuous recording of mouse behavior for six minutes in the home cage: two minutes prior to laser stimulation, followed by optogenetic stimulation for two minutes and recovery from optogenetic stimulation for the final two minutes. There is significant, strong stimulation of grooming with short latency, following initiation of laser illumination in the DMS and mPFC (Figs. 1, 2). The required levels of laser power (6.8–8.5 mW) were empirically determined and likely reflects the number of *Hoxb8* microglia that are needed to be activated to elicit the behavioral output in these brain regions. The findings were replicated in both male and female mice with no apparent differences observed between them (i.e., no sex bias Fig. 1b–h, j–p and Fig. 2b–h, j–p). Further statistical analysis of pair-wise comparisons were made between the data sets of each sex for significance differences in grooming responses following laser exposure in both the DMS and mPFC and none were found (see Figure legends for both Figs. 1 and 2 for the data). The scales on the y axis, which differ in separate sets of experiments, were chosen to allow inclusion of all the data points pertaining to each experiment. It is of interest that the recovery from laser exposure (i.e., cessation of grooming) which can occur in seconds appears to be more rapid and complete in the mPFC than in the DMS. Also, the grooming response to laser activation of *Hoxb8* microglia appears more robust in the mPFC than in the DMS.

We verified histologically the sites of cannula implantation and imaged microglia within the field of stimulation using high resolution [63× and 20×] confocal microscopy immediately after the recovery period (Supplementary Figs. 2, 3). The distribution and morphology of *Hoxb8* microglia within these regions appear normal and indistinguishable from tissue not exposed to the laser. Further, 100% of *Hoxb8* microglia identified with YFP produced in Ai32 mice (following *Hoxb8*^*IRESCre*^ transcriptional activation) co-labeled with Iba1, a pan microglia marker that labels all microglia (Supplementary Fig. 2). From these experiments, we conclude that we could not detect any signs of histological damage to *Hoxb8* microglia as a consequence of laser exposure. This is important because we have to consider the possibility that cellular damage could induce anxiety and grooming. Also, the recovery from laser exposure (termination of the behavior) often occurs in seconds. The induction of the behavior is reversible and stops when the laser is turned off. Such rapid recovery from exposure to the laser is inconsistent with potential tissue damage induced by the laser. Finally, we have repeated the experiments successively over and over in the same mouse, and the rapid recovery period does not change. Even if a small amount of undetectable cellular damage occurred during each round, such damage should become cumulative and be detectable both with respect to behavior and morphology of *Hoxb8* microglia. Yet we cannot detect the consequences of such repeated hypothetical damage.

### Optogenetic stimulation of *Hoxb8* microglia in the BLA and CeA induces anxiety

**Fig. 3:**
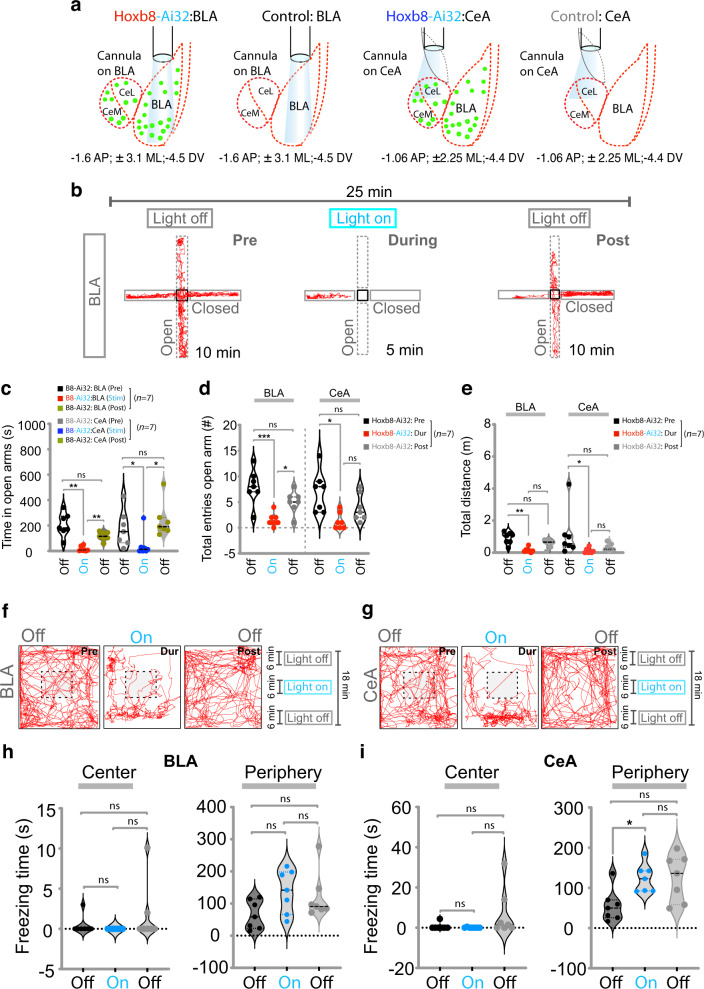
Optogenetic stimulation of Hoxb8 microglia within BLA and CeA induces anxiety behaviors. **a** Representation of cannula insertion and light (473 nm) delivery into BLA and CeA to stimulate *Hoxb8* microglia in *Hoxb8*-^IRES-Cre/+^;Ai32^/+^ or non-channelrhodopsin expressing C57/Bl6 control mice. A straight cannula was stereotaxically inserted in BLA at the coordinate (−1.6 mm AP; ± 3.1 mm ML; −4.5 mm DV) and a beveled cannula was inserted in CeA at the coordinate (−1.06 mm AP; ± 2.25 mm ML; and −4.4 mm DV). Experimental mice were pre-conditioned in the experimental room for 5 min before optogenetic stimulation. **b** Track plot of BLA implanted *Hoxb8*-^IRES-Cre/+^;Ai32^/+^ mice under pre, during and post stimulation conditions in an elevated plus maze test showing the preference of the experimental subject to remain in the closed platform in response to continuous optogenetic 473 nm laser stimulation of *Hoxb8* microglia. **c** Significantly reduced time in the open arms for the BLA and CeA implanted *Hoxb8*-^IRES-Cre/+^;Ai32^/+^ mice in response to continuous optogenetic (11.4 mW) stimulation relative to pre-stimulation conditions. **d**, **e** Significantly reduced open arm entries and total distance traveled in open arm for *Hoxb8*-^IRES-Cre/+^;Ai32^/+^ mice during photostimulation compared to pre and post stimulation condition. **f**, **g** Track plots for the BLA and CeA implanted *Hoxb8*-^IRES-Cre/+^; Ai32^/+^ mice in an open field showing a higher preference of the experimental subject to remain in the periphery compared to anxiety-sensitive center zone in response to 6 min of continuous photostimulation at 11.5 mW laser power. Increased (**h**, right panel) and significantly increased (**i**, right panel) total freezing time in periphery during photostimulation for the BLA (**h**, right panel) and CeA (**i**, right panel) implanted *Hoxb8*-^IRES-Cre/+^; Ai32^/+^ mice in the open field test without significantly affecting the freezing time at the center (**h**, left panel for BLA, **i**, left panel for CeA). Data comparison, Paired t test for **c**, Repeated measure One-way ANOVA with Geisser greenhouse correction and Tukey’s HSD posthoc multiple comparison test for **d**, **e**, **h**, **i**. ns, *P* > 0.05, not significant, *P < 0.05, **P < 0.001. 3-4 months old mice were used for BLA and CeA implantations. *n* = 7 mice for BLA and *n* = 6 mice for CeA.

Remarkably, optogenetic stimulation of *Hoxb8* microglia within the basolateral amygdala (BLA) and central amygdala (CeA) did not induce grooming, but instead induced elevated levels of anxiety. Neuronal circuits within these regions of the brain have previously been shown to be required for modulating levels of anxiety, fear response and emotion [9, 11–16]. We used the elevated plus maze and open field tests (OFTs) to assess levels of anxiety in mice. Continuous optogenetic stimulation of *Hoxb8* microglia for 5 min within the BLA and CeA induced robust anxiety-like behavior as measured by the elevated plus maze tests (i.e., obviating the open arm Fig. 3b–d, Supplementary Fig. 4c, d, i; a classic measure of anxiety in mice). The relatively longer laser stimulation periods (5 vs. 2 min) were required to allow video capture of sufficient data to be of significance on the elevated plus maze apparatus. During pre- and post-laser stimulation periods, the mice explored the open and closed arm equally (Fig. 3b–d), implying that the higher levels of anxiety are dependent on optogenetic stimulation of *Hoxb8* microglia. These results show that *Hoxb8* microglia within both the BLA and CeA induce similar levels of anxiety when optogenetically activated (Fig. 3c, d). During the initial time segment following laser stimulation, the total entries to the open (Fig. 3d; Supplementary Fig. 4i) but not to the closed arms (Supplementary Fig. 4j) were significantly decreased in *Hoxb8*^*IRESCre*^*/Ai32* mice compared to controls (i.e., C57/Bl6 mice Supplementary Fig. 4e, g). The total open arm entries quantified for the post-stimulation conditions were similar to during-stimulation conditions (Supplementary Fig. 4i) suggesting a slower recovery period following optogenetic stimulation of the BLA and CeA relative to the DMS and mPFC. The total distance traveled on the elevated maze during optogenetic stimulation of *Hoxb8* microglia in both the BLA and CeA decreased relative to pre-stimulation periods (Fig. 3e), due to mouse movement being interspersed with periods of freezing which was quantified (Fig. 3e; Supplementary Fig. 5c–l). This is a common observation when neurons in these regions are optogenetically stimulated [9, 12–14] and is indicative of higher levels of anxiety. The time spent in the open and closed arms and entry probability were not affected by laser exposure in control mice within the BLA and CeA (Supplementary Fig. 4c, 4e BLA, 4d, 4g, CeA, 6c, f). These control experiments not only argue for the requirement of optogenetic activation of *Hoxb8* microglia in the BLA and CeA being critical for the induction of anxiety, but also that laser exposure did not inflict significant damage to the cells within these brain regions, since the behavior of the control mice in the open and closed arms was not affected by equivalent laser stimulation in terms of both laser power and duration of laser exposure.

**Fig. 4:**
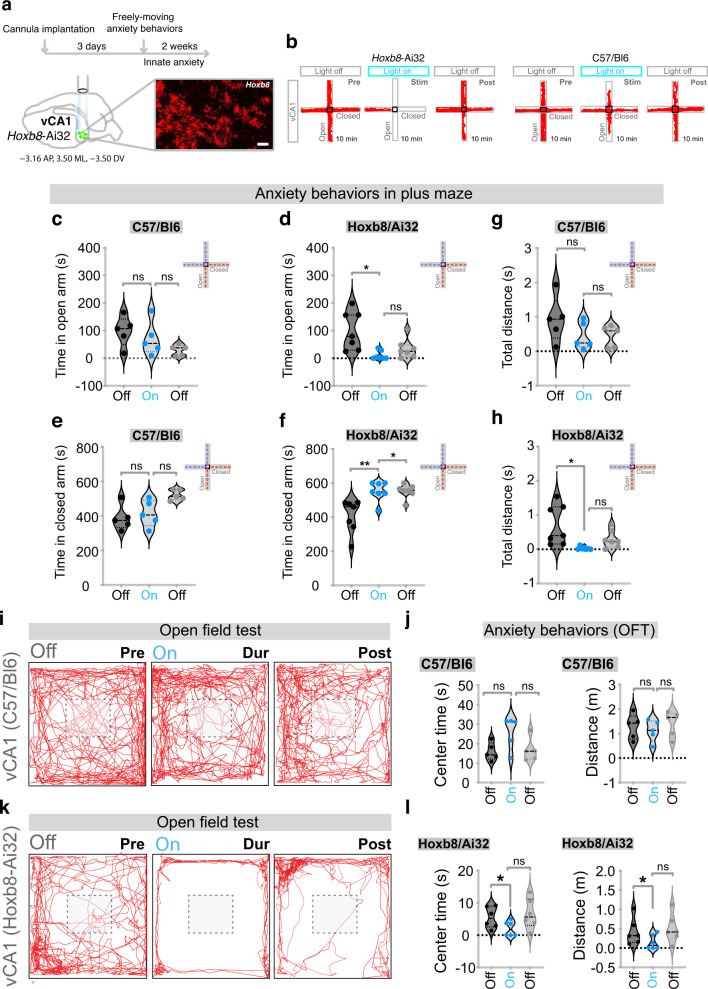
Optogenetic stimulation of Hoxb8 microglia within ventral CA1 region of hippocampus induces anxiety behaviors. **a** Experimental design and representation of cannula implantation of *Hoxb8*-^IRES-Cre/+^;Ai32^/+^ mice within the ventral CA1 region of the hippocampus. Shown is the blown up ventral CA1 region displaying *Hoxb8* microglia from *Hoxb8*^-IRES-Cre/+^;Rosa-^LSL-tdTomato^ mice. **b** Track plots of vCA1 implanted *Hoxb8*-^IRES-Cre/+^;Ai32^/+^ mice in response to photostimulation of *Hoxb8* microglia in an elevated plus maze test showing the preference of the experimental subject to remain in the closed platform in response to continuous optogenetic 473 nm laser stimulation of *Hoxb8* microglia at 2.3 mW laser power. Significantly reduced time in the open arms for vCA1 implanted *Hoxb8*-^IRES-Cre/+^;Ai32^/+^ (**d**) but not in vCA1 implanted C57/Bl6 mice (**c**) in response to 2.3 mW laser photostimulation. Significantly increased time in the closed arms of vCA1 implanted *Hoxb8*-^IRES-Cre/+^; Ai32^/+^ (**f**) but not in the vCA1 implanted C57/Bl6 mice (**e**). Significantly reduced total distance traveled in the open arm for vCA1 implanted *Hoxb8*-^IRES-Cre/+^;Ai32^/+^ (**h**) but not in C57/Bl6 mice (**g**) during photostimulation compared to pre or post stimulation condition. Track plots for the vCA1 implanted male C57/Bl6 (**i**) and *Hoxb8*-^IRES-Cre/+^;Ai32^/+^ mice (**k**) in an open field showing a higher preference of the experimental subject to remain in the periphery compared to anxiety-sensitive center zone in response to 6 min of continuous photostimulation at 2.3 mW laser power in *Hoxb8*-^IRES-Cre/+^;Ai32^/+^ (**k**) but not in C57/Bl6 mice (**i**). Significantly reduced time spent and distance covered in the center (**l**, left and right panel) of the open field for the *Hoxb8*-^IRES-Cre/+^; Ai32^/+^ but not C57/Bl6 (**j**, left and right panel) vCA1 implanted mice in response to photostimulation. Data comparison, Repeated measure One-way ANOVA with Geisser greenhouse correction and Tukey’s HSD posthoc multiple comparison test for **c**–**h**, and **j**, **l**. ns, *P* > 0.05 not significant, *P < 0.05, **P < 0.001. 3–4 months old male mice were used for vCA1 implantation. *n* = 5 mice for C57/Bl6, *n* = 7 mice for *Hoxb8*-^IRES-Cre/+^;Ai32^/+^ for plus maze test. *n* = 5 mice for C57/Bl6, *n* = 6 mice for *Hoxb8*-^IRES-Cre/+^;Ai32^/+^ for open field test.

To further examine the anxiety behavior elicited by optogenetic stimulation of *Hoxb8* microglia in the BLA and CeA, we used OFTs to assess levels of anxiety, including freezing behavior (Fig. 3f–i). In response to continuous optogenetic stimulation of *Hoxb8* microglia for six minutes within the BLA and CeA, *Hoxb8*^*IRESCre*^*/Ai32* mice (but not control mice) showed reduction in time spent in the center as well as increases in the total freezing time (Fig. 3f, h, BLA, 3g, i, CeA, Supplementary Fig. 7b–f, m, n). Again, the relatively longer times of laser application in the open field tests (6 vs. 2 min) were used to allow video capture of sufficient data to reach significance. The latter results corroborate the results we obtained with the elevated plus maze tests in terms of anxiety as measured by reduced time spent exploring the central domain in OFTs (Fig. 3f–g; Supplementary Fig. 7m, n) as well as in increased freezing time in periphery (Fig. 3h, i; Supplementary Fig. 7b BLA, 7f CeA *Hoxb8*; 7d C57bl/6 BLA, 7h CeA C57bl/6). Supplementary Fig. 6 provides further data that control mice do not respond to laser exposure within the BLA or CeA as assayed on the elevated plus maze or open field tests. We again confirmed that the distribution and morphological features of *Hoxb8* microglia exposed to the laser for longer periods of time within the BLA and CeA are not distinguishable from healthy and laser-unstimulated microglia within these brain regions (Supplementary Fig. 8).

### Optogenetic stimulation of *Hoxb8* microglia in the ventral CA1 hippocampus (vCA1) induces both anxiety and grooming as well as freezing

Recent reports have pointed to the presence of neuronal circuits within the vCA1 that are capable of encoding anxiety behavior [17, 23]. Would optogenetic stimulation of *Hoxb8* microglia within the vCA1 induce anxiety? Remarkably, not only anxiety but also grooming as well as freezing was induced by optogenetic stimulation of *Hoxb8* microglia in the vCA1. In Fig. 4b–f we show that following laser stimulation in the vCA1, *Hoxb8*^*IRESCre*^*/Ai32* mice but not control mice, preferred to remain within the closed arm of the elevated plus maze. The open arm time and distance covered were significantly reduced (Fig. 4d, h) while the time spent within the closed arm significantly increased with *Hoxb8*^*IRESCre*^*/Ai32* mice (Fig. 4f). In the OFT, *Hoxb8*^*IRESCre*^*/Ai32* mice that received laser stimulation spent significantly reduced total time in the center (Fig. 4k, l; Supplementary Fig. 10c) relative to controls (Fig. 4i, j; Supplementary Fig. 10b), preferred the periphery (Fig. 4k; Supplementary Fig. 10c) and spent less time at the center and covered less total distance (Fig. 4k, l; Supplementary Fig. 9d). The robust anxiety-like behaviors observed in *Hoxb8*^*IRESCre*^*/Ai32* mice were in stark contrast to C57Bl/6 control mice that equally explored the entire open field prior to, during and after laser stimulation (Fig. 4i, j; Supplementary Fig. 10b). In addition, the freezing time and number of episodes observed in *Hoxb8*^*IRESCre*^*/Ai32* mice compared to control mice were also significantly greater (Supplementary Fig. 10d, e). The elevated plus maze test also detected increased freezing time in *Hoxb8*^*IRESCre*^*/Ai32* mice compared to C57Bl/6 mice (Supplementary Fig. 10f, h) These data support the conclusion that optogenetic activation of *Hoxb8* microglia in the vCA1 induces a robust anxiety response.

Of special interest, significantly lower laser power of ~2.3 mW was sufficient to fully induce higher levels of anxiety as measured by both elevated plus maze and OFT compared to the BLA and CeA. In addition, the latency time for initiating anxiety behavior in the vCA1 (a couple of seconds) was much shorter than the requirement for prolonged (min) stimulation of the BLA or CeA brain regions.

**Fig. 5:**
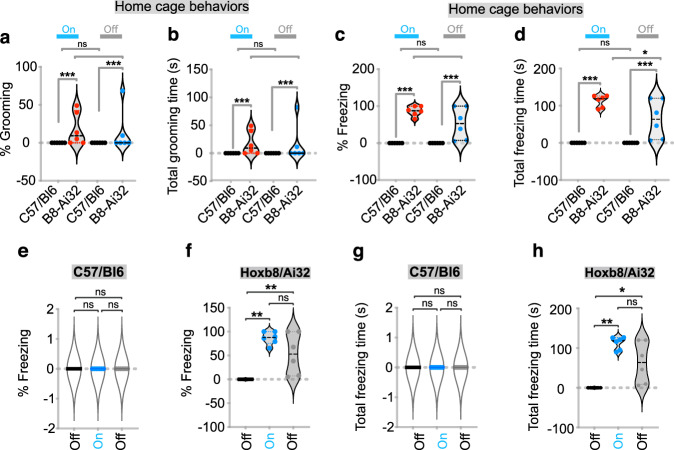
Optogenetic stimulation of Hoxb8 microglia within vCA1 region of hippocampus induces freezing and grooming behaviors in the home cage. Significant increase in the percentage of phase III facial grooming (**a**) and total grooming time (**b**) in *Hoxb8*-^IRES-Cre/+^; Ai32^/+^ compared to C57/Bl6 mice during photostimulation of *Hoxb8* microglia at 2.3 mW laser power and during post stimulation period. Significant increase in the percentage of freezing (**c**) and total freezing time (**d**) in *Hoxb8*-^IRES-Cre/+^; Ai32^/+^ compared to C57/Bl6 mice during photostimulation of *Hoxb8* microglia at 2.3 mW laser power (**e**–**h**). Increased percentage grooming and total freezing time in *Hoxb8/Ai32* compared to C57/Bl6 mice. Data represents video recordings acquired in the home cage environment and analyzed for measuring grooming and freezing behaviors. Data comparison, Two-tailed Mann-Whitney *U* test was used for **a**–**d**, Repeated measure one-way ANOVA with Geisser greenhouse correction and Tukey’s HSD posthoc test for group comparison for **e**, **g**, **i**, **k**. Kurskal-Wallis test followed by Dunn’s multiple comparison test for **f**, **h**, **j**, **l**. ns, *P* > 0.05 not significant, *P < 0.05, **P < 0.001. 3–4 months old mice were used for vCA1 implantation.

In the home cage, we observed strong induction of both grooming and anxiety in response to optogenetic stimulation of *Hoxb8* microglia in the vCA1 (Fig. 5). However, in contrast to the induction of grooming by optogenetic stimulation in the DMS or mPFC (requiring ~6.6–8.5 mW of power), a lower laser power (2.0 mW) was sufficient to induce grooming. The behavioral responses to vCA1 laser stimulation was primarily freezing interspersed with strong bouts of phase III and phase IV grooming behavior (Fig. 5a–h; Supplementary Fig. 9f–i). Controls exhibited no grooming or freezing bouts (Fig. 5a–e, g). In the DMS and mPFC, we did not observe mice freezing in response to optogenetic stimulation of *Hoxb8* microglia, a likely reflection that anxiety-like behavior is not induced by optogenetic activation of *Hoxb8* microglia in the DMS and mPFC. What is apparent is that the vCA1 hippocampus is very sensitive to optogenetic stimulation of *Hoxb8* microglia with respect to both the induction of anxiety and grooming, with the added propensity for freezing, a further reflection of anxiety. The high sensitivity of the vCA1 is reflected in terms of the response to laser exposure, in terms of power requirements, in the quickness of behavioral responses and the broad range of behavioral responses. We have examined the morphology of microglia cells within the vCA1 region of the hippocampus post-laser stimulation and they appear indistinguishable from laser unstimulated microglia (Supplementary Fig. 9b).

### Optogenetic stimulation of both *Hoxb8* microglia and non-Hoxb8 microglia in the different regions of the brain using *Iba1*^*IRESCre*^

Thus far, we have determined the behavioral consequences of optogenetic activation of *Hoxb8* microglia in the DMS, mPFC, BLA, CeA and vCA1. What are the behavioral consequences of activating non-*Hoxb8* microglia in these same regions of the brain? Unfortunately, we cannot directly do such experiments because to date a genetic marker specific to non-*Hoxb8* microglia has not been identified. However, we can optogenetically activate both microglial subpopulations, using *Iba1*^*IRESCre*^, which is expressed in both microglia subpopulations, and compare these results to optogenetic activation of only *Hoxb8* microglia with *Hoxb8* ^*IRESCre*^. We have previously described the construction of *Iba1*^*IRESCre*^ allele and shown that it functions well within all microglia [22]. Our expectations for these experiments were that either 1) no behavioral differences would be observed between activation of both microglial populations, within the appropriate region of the brain with *Iba1*^*IRESCre*^, compared to only activating *Hoxb8* microglia with *Hoxb8*^*IRESCre*^, indicating that non-*Hoxb8* microglia do not participate in the induction of grooming and anxiety or 2) higher levels of grooming and anxiety would be observed by activating both microglial populations, again in the appropriate regions of the brain, compared to activation of only *Hoxb8* microglia, suggesting that both microglial populations participate in the induction of the behaviors.

**Fig. 6:**
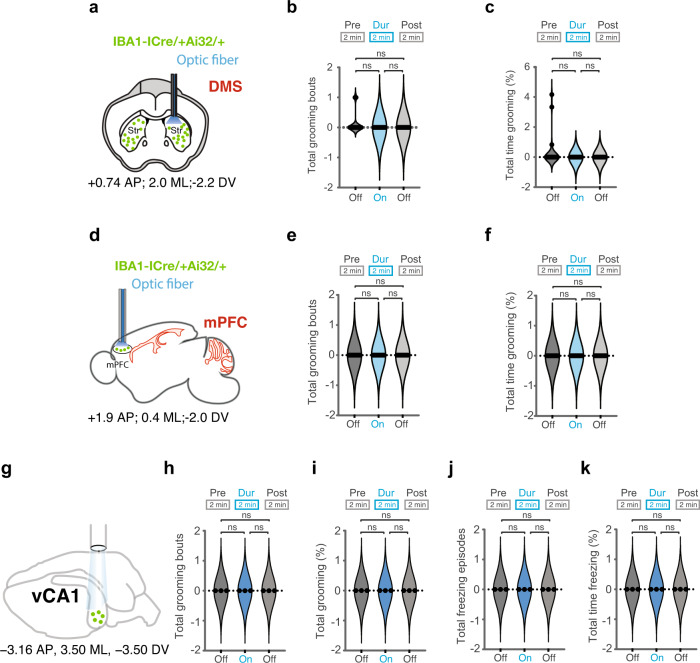
Optogenetic stimulation of both microglia subpopulations within DMS, mPFC, and vCA1 with Iba1-Cre fail to induce any grooming and freezing behaviors. **a** Representation of cannula insertion and light (473 nm) delivery into DMS to stimulate all microglia unilaterally in IBA1-^IRES-Cre/+^; Ai32^/+^ male mice. Shown in the picture (**a**) is a straight cannula that was stereotaxically inserted within DMS in the right brain hemisphere at the coordinates +0.74 AP, + 2.0 ML, −2.2 DV. Stimulation condition consisted of a two-minute baseline recording followed by 2 minutes of continuous optogenetic stimulation at 473 nm and 2 min of post stimulation recording. No significant change in the total number of grooming bouts (**b**) or the percentage of grooming time (**c**) in response to DMS stimulation of *IBA1*-^IRES-Cre/+^; Ai32^/+^ mice. **d** Representation of cannula insertion and light (473 nm) delivery into mPFC to stimulate all microglia unilaterally in IBA1-^IRES-Cre/+^; Ai32^/+^ female mice. Shown in the picture (**d**) is a straight cannula that was stereotaxically inserted within mPFC in the right brain hemisphere at the coordinates +1.9 AP, + 0.4 ML, −2.0 DV. Stimulation condition consisted of a 2-min baseline recording followed by 2 min of continuous optogenetic stimulation at 473 nm and 2 min of post stimulation recording. No significant change in the total number of grooming bouts (**e**) and the percentage of grooming time (**f**) in response to mPFC stimulation of IBA1-^IRES-Cre/+^; Ai32^/+^ mice. **g** Representation of cannula implantation of IBA1-^IRES-Cre/+^; Ai32^/+^ male mice within the ventral CA1 region of the hippocampus in the right brain hemisphere at the stereotactic coordinates −3.16 AP, 3.50 ML, −3.5 DV. No significant change in the total number of grooming bouts (**h**) and the percentage of grooming time (**i**) in response to vCA1 stimulation of *IBA1*-^IRES-Cre/+^; Ai32^/+^ mice. No significant change in the total number of freezing episodes (**j**) or the percentage of freezing time (**k**) in response to vCA1 stimulation. These results are in marked contrast to the results obtained by optogenetic stimulation of only *Hoxb8* microglia in the comparable regions of the brain (Figs. 1, 2, and 5) ns, not significant. Repeated measure One-way ANOVA with Geisser greenhouse correction and Tukey’s HSD posthoc multiple comparison tests were used for the figures **b**, **c**, **e**, **f**, **h**–**k**. 3-4 months old mice were used for the cannula implantation within DMS (*n* = 5 mice, males), mPFC (*n* = 4 mice, females) and vCA1 brain regions (*n* = 3 mice, males).

Instead, the experimental results were totally unexpected, no behaviors were induced (Fig. 6). Optogenetic activation of both microglia subpopulations in the appropriate regions of the brain resulted in quenching the behaviors induced by activation of *Hoxb8* microglia alone. For example, as shown in Fig. 6a–f, optogenetic activation of both microglial subpopulations using *Iba1*^*IRESCre*^ within either the DMS or mPFC do not induce grooming (Supplementary Fig. 11b–g, Fig. 12c–f), whereas optogenetic activation of only *Hoxb8* microglia in either region of the brain induces robust grooming (Figs. 1, 2). A trivial explanation that the *Iba1*^*IRESCre*^ driver did not work was ruled out by showing expression of *YFP* in all microglia. Showing that YFP is expressed necessarily means that ChR2 is also expressed, because the YFP and ChR2 are present in Ai32 as a fusion gene leading to production of a YFP/ChR2 fusion protein, both being dependent upon Cre for activation (Supplementary Fig. 12b). Further, in the home cage we reported the induction of both grooming and anxiety by optogenetic stimulation of only *Hoxb8* microglia in the vCA1 (Figs. 4, 5). Induction of anxiety was particularly evident from the extensive induction of freezing in these experimental mice. As also shown in Fig. 6h–k the characteristic grooming and freezing behavior was eliminated when *Iba1Cre* was used to optogenetically stimulate both microglial subpopulations within the vCA1. Thus, the levels of both grooming (in the DMS, mPFC and vCA1) and anxiety (in the vCA1) are eliminated by co-stimulation of both populations of microglia (Fig. 6) This is in marked contrast with the behaviors that are induced when only *Hoxb8* microglia are optogenetically stimulated within the appropriate regions of the brain (Figs. 1, 2, 4, and 5).

These unanticipated results of optogenetic activation of both microglia populations in the appropriate regions of the brain have completely revised how we view the role of *Hoxb8* and non-*Hoxb8* microglia in modulating the levels of grooming and anxiety in mice. These optogenetic results suggest that the two subpopulations of microglia work in opposition to each other. For example, *Hoxb8* microglia function to down regulate anxiety and grooming (i.e., function as brakes), whereas non-*Hoxb8* microglia function to upregulate anxiety and grooming (i.e., function as an accelerator). It appears that optogenetic stimulation of both populations of microglia in the DMS, mPFC and vCA1 results in canceling communication with neurons required to induce the behaviors. This model provides a biological reason for the existence of two populations of microglia in mice and provides a mechanism by which the two populations of microglia working in concert could provide fine control of the levels of anxiety and grooming to adjust to changing homeostatic conditions. Further, the model is consistent with the genetic disruption of *Hoxb8* resulting in chronic anxiety and pathological overgrooming in mice. In the absence of brakes, the accelerator takes over and chronic anxiety and pathological overgrooming ensue.

### Optogenetic stimulation of *Hoxb8* microglia in mPFC brain slices increases neuronal activity in neighboring neurons

**Fig. 7:**
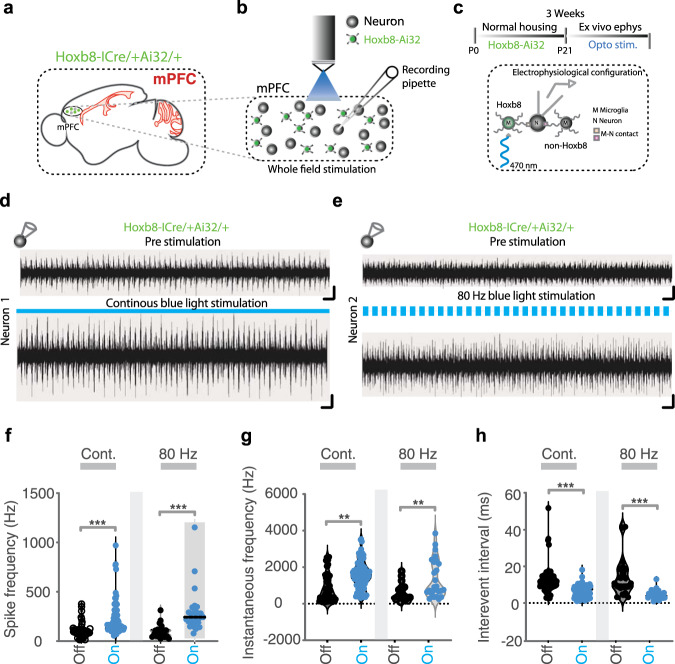
Optogenetic stimulation of Hoxb8 microglia in specific regions of the brain alters neighboring neuronal activity. **a** Representation of the location of mPFC used for optogenetics in brain slice preparation in *Hoxb8*-^IRES-Cre/+^;Ai32^/+^ male mice (3 week old). Zoomed portion of the *Hoxb8* microglia and neurons exposed to whole field optogenetic stimulation at 470 nm wavelength. Shown is a recording pipette that was positioned on a neuron nearby *Hoxb8* microglia (**b**) and a cartoon (**c**) of electrophysiological configuration. Representative traces from two independent (**d**, Neuron 1, **e**, Neuron 2) patched neurons under pre-stimulation and during continuous (**d**) or during 80 Hz (**e**) blue light phasic stimulation. Scale bar, 30 pA and 50 ms. **f**–**h** Spike frequency (**f**) and instantaneous frequency (**g**) are significantly increased but inter-event interval is significantly decreased (**h**) in cell attached neuronal recording in response to continuous or 80 Hz optogenetic whole field stimulation of *Hoxb8* microglia in brain slice preparation. Individual data point represents the spike activity of the recorded neuron from individual traces. Data represents spike analysis over multiple traces from individual cell-attached neuronal recordings performed blindly from three brain samples under pre-stimulation and during optogenetic stimulation conditions (*n* = 10 cells for continuous stimulation, *n* = 10 cells for 80 Hz stimulation). Data comparison, Wilcoxon matched-pairs signed rank test. ns, *P* > 0.05 not significant, ***P < 0.0001.

To address the question of direct neuronal response to optogenetic microglia stimulation we employed whole field optogenetic activation of *Hoxb8* microglia using mPFC-containing frontal cortical brain slice preparations derived from *Hoxb8*^*IRESCre*^*/Ai32* mice. As illustrated in whole field phasic (80 Hz) or continuous laser stimulation of *Hoxb8* microglia within the mPFC does indeed result in significantly increased neuronal spike activity of patched neighboring neurons (Fig. 7d–h). As a control, the experiment was repeated using brain slice preparations from the same region of the brain but derived from mice lacking the *Hoxb8*^*IRESCr*e^/*Ai32* alleles. Following the same laser exposure protocol yielded no increased neuronal spike activity in the neighboring neurons (Supplementary Fig. 14). We further characterized spike activity and quantified multiple spike parameters. Both continuous and phasic (80 Hz) whole field optogenetic stimulation of *Hoxb8* microglia indeed significantly increased the spike frequency and instantaneous frequency of the patched neurons (Fig. 7f, g) while significantly reduced the inter-event intervals (Fig. 7h) without affecting half width, rise time, decay time to peak or the normalized spike amplitude (Supplementary Fig. 15a–e). Thus, optogenetic activation of *Hoxb8* microglia within brain slice preparations derived from *Hoxb8*^*IRESCre*^*/Ai32* mice lead to significantly increased neuronal activity within neighboring neurons. Further, the measurement of current–voltage (*I*–*V*) relationship of the patched neurons mediated by cations at stepping membrane potential revealed a shift in the *I*–*V* curve with high sensitivity to the duration of light exposure and stimulation frequencies (Supplementary Fig. 16d, e). Only high frequency (80 Hz) stimulation induced shift in the *I*–*V* curves compared to low frequency (10 Hz) stimulation (Supplementary Fig. 16f, g).

### Expression of c-fos in neighboring neurons and Kv1.3 in *Hoxb8* microglia in response to optogenetic stimulation of *Hoxb8* microglia

Having shown that optogenetic stimulation of *Hoxb8* microglia results in the activation of neighboring neurons, the question arises whether the neural activation proceeds through the induction of the immediate early transcriptional response including *c-fos* protein production [24]. Supplementary Fig. 17a, b indeed illustrates significantly increased neuronal *c-fos* protein production in the neighboring neurons in response to laser exposure of *Hoxb8*^*IRESCre*^*/Ai32* mice at the site of optogenetic activation of *Hoxb8* microglia (i.e., in the vCA1 region of the hippocampus). As a control, c-fos protein could not be detected in brain slices at comparable brain positions on the contralateral side of the same mouse which was not exposed to laser activation (-light, Supplementary Fig. 17a).

Similarly, we have used immunofluorescence to determine if optogenetic activation of *Hoxb8* microglia leads to depolarization of the *Hoxb8* membrane, as assessed by the production of Kv1.3, a subtype of potassium channels used as a marker for the detection of membrane depolarization [21]. Supplementary Fig. 18 shows that upon laser activation of Hoxb8 microglia for 2 minutes within the vCA1 that induced immediate freezing behavior, markedly, significantly increased the expression of Kv1.3 protein as detected by immunohistochemistry (Supplementary Fig. 18a–c and M1–M4) within cell bodies and projections of *Hoxb8* microglia at the site of laser activation (+ light). As controls we examined *Hoxb8* cells in the tissue slices derived from comparable positions of the brain on the contralateral, non-laser exposed side of the same mouse and did not detect any expression of the Kv1.3 protein expression (- light). With respect to the Kv1.3 experiments in response to optogenetic stimulation of all microglia using *Iba1*^*IRESCre*^*/Ai32* mice the answer appears a bit more complex. Upon Iba1-Cre laser stimulation, large similar numbers of microglia produce Kv1.3 protein but the number of Kv1.3 foci per cell appear to be significantly reduced (Supplementary Fig. 18h). However, though the number of Kv1.3 foci/cell is smaller, the size of the foci appears to be consistently larger (compare Kv1.3 foci in microglia following Hoxb8 or *Hoxb8* + non-*Hoxb8* microglia optogenetic stimulation. M1-M4 in each case show representative, but a small sampling of total microglia examined. These results could indicate that we may be observing a transition of Kv1.3 from monomeric (Hoxb8-Cre stimulation) to a polymeric (Iba1-Cre stimulation) state. What we can conclude is that microglia membrane depolarization as measured by the robust production of Kv1.3, a marker of membrane depolarization, occurs in both *Hoxb8* microglia and total microglia following optogenetic stimulation of either *Hoxb8* microglia alone or in combination with both microglia populations using *Hoxb8*^*IRESCre*^ or *Iba1*^*IRESCre*^ respectively.

Having used c-fos and Kv1.3 immunohistochemistry to illustrate 1) that neighboring neurons respond to optogenetic stimulation of *Hoxb8* microglia in the appropriate regions of the brain by induction of neuronal immediate early transcription and 2) that optogenetic stimulation of *Hoxb8* microglia or all microglia lead to depolarization of their microglial membranes, we can further use this methodology to determine if c-fos is also produced at higher levels in response to optogenetic stimulation of both microglial subpopulations using Iba1-Cre (Supplementary Fig. 17). This experiment is of immediate importance because optogenetic stimulation of both microglial subpopulations lead the quenching of both grooming and anxiety behaviors. Supplementary Fig. 17c–e, show that significantly increased c-fos protein expression in neighboring neurons does not occur when both microglia populations are optotenetically activated by *Iba1*^*IRESCre*^. These experiments lend independent support for the model that the two populations of microglia, *Hoxb8* microglia and non-*Hoxb8* microglia, work in opposition to each other canceling all signals and therefore a responding signal is not emitted by neighboring neurons. It also illustrates that one of the critical consequences of optogenetic stimulation of *Hoxb8* microglia in appropriate regions of the brain is the significant induction of the c-fos-immediate early response in neighboring neurons.

### Specificity of the optogenetic experiments (Ai32, cell types, positions within the brain and Cre driver)

To conclude the *Results*, it is important to outline the controls that establish the specificity of the optogenetic experiments starting with the reported leakage of the Ai32 allele to the specificity of the *Hoxb8-Cre* driver in the brain.

It has been reported that the *Ai32* allele [25], a Cre dependent allele of Channelrhodopsin2 (ChR2) labeled with YFP, exhibits spurious neuronal expression of *ChR2* in non-Cre expressing neurons, resulting from read-through of the LSL cassette used to make *ChR2* and YFP transcription be dependent upon Cre [26]. Could this spurious neuronal expression of *ChR2* account for the induction of the grooming and anxiety behaviors? The spurious expression of *ChR2* is very low relative to the levels of expression observed in Cre expressing cells. For example, this leaky expression can only be detected in mice that are homozygous for the Ai32 allele. A two-fold reduction of ChR2 and YFP expression in Ai32 heterozygous mice render this expression level to be below detection [26]. Our breeding protocol generates experimental animals that are always heterozygous for Ai32 and we have confirmed the inability to detect spurious ChR2 and YFP neuronal expression in our experimental mice (Supplementary Fig. 19). The four panels shown are representative of the many that were examined. Further, we have shown that Ai32 mice, even when homozygous, in the absence of *Hoxb8*^*IRESCre*^, cannot be induced to groom or exhibit higher levels of anxiety by providing laser exposure (1.7–2.8 mW) within the vCA1, using laser power and duration of laser exposure that would normally induce grooming and anxiety in mice carrying all of the alleles needed to induce both behaviors (Figs. 4, 5; Supplementary Fig. 20 b–l). Further, anxiety as assessed by freezing behavior is also not observable in these control mice (Supplementary Fig. 20). These experiments argue not only that *Hoxb8*^*IRESCre*^ is required to activate ChR2 in *Hoxb8* microglia, to enable the induction of both behaviors in the vCA1, but also that any possible spurious expression of ChR2 in neurons, irrespective of their level of expression, detectable or not detectable (i.e., homozygous or heterozygous for the Ai32 allele, respectively), cannot be responsible for the induction of either behavior, since none was observed, in the absence of *Hoxb8*^*IRESCre*^. Potential leakage of *Ai32* in *neurons* should not be affected by the presence or absence of *Hoxb8*^*IRESCre*^ in microglia.

As controls, we have also tested several brain regions for optogenetic induction of *Hoxb8* microglia dependent grooming such as in the dorsolateral striatum (DLS) and the ventral medial striatum (VMS) (Supplementary Fig. 21). We have not observed significant induction of grooming through laser stimulation of *Hoxb8* microglia in the DLS. However, there does appear to be a lower but significant level of grooming induced by optogenetic activation of *Hoxb8* microglia in the VMS compared to the DMS (Supplementary Fig. 21d–f).

Optogenetic activation of *Hoxb8* microglia in other cortical regions such as the primary visual cortex or non-cortical regions, such as the retrotrapezoid nucleus (RTN) of the hindbrain, did not elicit any detectable grooming or freezing behavior (Supplementary Figs. 22, 23). These control experiments argue that grooming requires induction by *Hoxb8* microglia in precise regions of the brain in keeping with the need for spatial specificity.

We have also tested optogenetic activation of other cell types within the DMS such as astrocytes (using Cre-dependent channelrhodopsin (Ai32) and *GFAP-Cre* for optogenetic activation) without detecting any observable behaviors, (i.e., grooming, Supplementary Fig. 24 and anxiety as reflected by freezing, Supplementary Fig. 25). As a control, to ensure that *GFAP-Cre* properly activated channelrhodopsin2 transcription in astrocytes, we examined these cells for the production of YFP, which is also under the control of Cre in the Ai32 allele (as a fusion protein). Supplementary Fig. 24j illustrates strong YFP expression (green) in astrocytes comparable to YFP expression in *Hoxb8*^*IRESCre*^*/Ai32* mice, showing that *ChR2* was activated, but subsequently these activated astrocytes did not send signals to the neighboring neurons to promote behaviors such as grooming and/or anxiety. As a size comparator in the same tissue sample, for the much larger astrocytes, we included the marker *Iba1* (red) which labels all microglia and found no coincidence with the larger astrocyte labeling.

Another important final consideration is the transcriptional specificity of *Hoxb8*^*IRESCre*^ since we rely on this Cre driver to restrict optogenetic activation to *Hoxb8* microglia. We have previously reported that the expression of *Hoxb8*^*IRESCre*^, using the reporter *ROSA26*^*CAGLSLGFP*^ is indistinguishable from the wild-type mouse *Hoxb8* transcription profile [4]. We and others have not detected *Hoxb8* transcripts in mouse brains throughout development or in adult brains [5, 27]. The data provided by the Allen Institute is also in good agreement with our results, reporting no detectable *Hoxb8* transcripts throughout the mouse adult brain [https://mouse.brain-map.org/gene/show/15191]. There is *Hoxb8* expression in the peripheral nervous system (i.e., in the dorsal region of the spinal cord) and correspondingly, *Hoxb8* mutant mice show lower sensitivity to pain [4, 27]. However, pathological overgrooming and lower sensitivity to pain observed in *Hoxb8* mutant mice can be cleanly separated by conditional mutagenesis [4]. Conditional disruption of *Hoxb8* only in the hematopoietic lineage of *Hoxb8*^*F/F*^ mutants, elicits only excessive grooming and these mice did not exhibit insensitivity to pain. Conversely, disruption of *Hoxb8* in the spinal cord only caused the nociceptive phenotype [4]. These results strongly argue that the expression of *Hoxb8* in the peripheral nervous system should not affect our optogenetic experiments in the brain.

Since *Hoxb8* is not expressed in the brain during development or postnatally, then where and when is the *Ai32* expression turned on in *Hoxb8* microglia? Initiation of transcription occurs in *Hoxb8* microglia progenitor cells, prior to their entry into the brain [5]. But once *Ai32* expression has been turned on by Cre, channelrhodopsin2 and YFP will be constitutively produced in *Hoxb8* microglia throughout their lifetime.

## Discussion

We have demonstrated that optogenetic activation of *Hoxb8* microglia in specific regions of the brain induces higher levels of anxiety, grooming or both. These experiments demonstrate a direct connection between optogenetically stimulated *Hoxb8* microglia and the neurons/neuronal circuits responsible for inducing and modulating these two behaviors. The above results raise numerous questions:

1. By what mechanisms do stimulated *Hoxb8* microglia communicate with the responding neurons? Does communication involve specific molecules such as ligands?
2. Are the same or different *Hoxb8* microglia communication mechanisms involved in different brain regions that show different behavioral outputs?
3. Which neuronal subpopulations respond to activated *Hoxb8* microglia and are they the same or different in different regions of the brain?
4. What are the responses in addition to *c-fos* upregulation by the recipient neurons to optogenetically activated *Hoxb8* microglia and are they the same or different in different regions of the brain? Do these responses provide clues as to mechanisms used to discriminate between the induction of region-specific behaviors?

We are now positioned to address the above questions using the *Hoxb8* microglia optogenetic experimental paradigm described herein to provide a much deeper molecular insight as to how the levels of anxiety and grooming are controlled by microglia within specific regions of the brain.

We have also shown that neighboring neurons do respond to optogenetically stimulated *Hoxb8* microglia using mPFC brain slice preparations derived from *Hoxb8*^*IRESCre*^*/Ai32* mice, but not in control mice lacking the *Hoxb8* Cre driver and the *Ai32* allele. Significant neuronal responses were assessed by determining the increased spike frequency, instantaneous frequency and reduction of the inter-event interval. Once the neurons that normally responds to optogenetically activated microglia are defined then genetic manipulation of these neurons should allow extending these results in vivo and in vitro. It is of interest that one mechanism by which neighboring neurons respond to optogenetic stimulation of *Hoxb8* microglia is the induction of the immediate early transcriptional response involving c-fos production.

Readers might view the finding that disruption of *Hoxb8* or ablation of the *Hoxb8* microglia cell lineage gives rise to pathological levels of anxiety and grooming, whereas optogenetic activation of *Hoxb8* microglia results in elevated levels of anxiety and grooming as being paradoxical. However, if the normal role of *Hoxb8* microglia is to inhibit (down regulate) anxiety and grooming behavior, and optogenetic activation of *Hoxb8* microglia release them from this inhibitory role, then the paradox is resolved.

A number of surprises resulted from the *Hoxb8* microglia optogenetic experiments. One was that they produced well defined and unexpected behavioral results: anxiety, grooming and freezing. Secondly, depending upon which region of the brain *Hoxb8* microglia were optogenetically activated, the behavioral outputs differed. Interrogating the differences among the brain regions may provide the means for identifying the molecular mechanisms that give rise to these choices. Finally, the parameters of laser illumination to optimize behavioral outputs markedly differed in different regions of the brain, emphasizing the importance of the local environment in controlling the behavioral output.

Perhaps the most surprising result is that co-optogenetic activation, using *Iba1*^*Cre*^, of both non-*Hoxb8* microglia and *Hoxb8* microglia in the DMS, mPFC and vCA1 results in quenching grooming in the former and both grooming and anxiety in the vCA1. These unexpected results strongly suggest that the two microglia populations in mice play opposing roles in modulating grooming and anxiety, with *Hoxb8* microglia functioning to downregulate these behaviors (i.e., function as brakes), and non-*Hoxb8* microglia function to upregulate these behaviors (i.e., function as an accelerator). Together these roles could allow fine tuning the levels of both grooming and anxiety to establish new setpoints. The model that the two microglia populations work in opposition to each other to modulate the levels of these behaviors is consistent with all of the data we have acquired to date on the biology of the two microglia populations, including cell transplantation experiments and the effects of genetic ablation of *Hoxb8* [5, 6]. This model provides a new way to view microglia activity and further provides a biological reason for mice having two populations of microglia with distinct ontogenies. That c-fos expression is upregulated in neighboring neurons to optogenetically stimulated *Hoxb8* microglia but not following optogenetic stimulation of both microglial subpopulations, lends independent support for the model that *Hoxb8* and non-*Hoxb8* microglia functioning in opposition to each other in regulating the levels of grooming and anxiety in mice. In retrospect, we should have suspected that *Hoxb8* and non-*Hoxb8* microglia work in opposition to each other earlier, based on the severity of the behavioral phenotype resulting from disruption of the *Hoxb8* gene in mice, chronic anxiety and pathological overgrooming.

Optogenetics, as outlined in the text, has been extensively used to define the neuronal components associated with stress/anxiety [9–17]. In these cases, the sites of neuronal circuitry and where induction of microglia optogenetically induce anxiety, closely align. Neuronal optogenetic sites that upon activation induce grooming have also been defined [https://mouse.brain-map.org/gene/show/15191]. Significantly, the neurons which have been most fully investigated in terms of controlling grooming behavior through optogenetic stimulation are the Island of Calleja neurons [28, 29]. These neurons reside within the VMS, which we have shown exhibits significant elevation of grooming following activation of *Hoxb8* microglia within this region. These observations highlight these neurons as being of special interest as potential targets for the analysis of microglia/neuronal interactions with respect to optogenetic *Hoxb8* activation in the induction of grooming.

In the introduction we emphasized that the level of anxiety and associated pathologies is an enormous world health problem. But, only through a greater molecular understanding of the neurobiology underlying the pathological manifestations of anxiety and the associated disorders can we hope to begin to curb these disorders. Development of drugs that modulate either or both microglia subpopulations, non-*Hoxb8* and *Hoxb8* microglia, could play major roles in the establishment of the next generation treatments for these neuropsychiatric disorders. A final question that should be considered is whether optogenetic activation of *Hoxb8* microglia is a “novel” trigger faced by these cells that fortuitously, when applied in the appropriate regions of the brain induces very specific behavioral outputs, anxiety and/or grooming and/or freezing, or is it instead a reflection of a normal process involved in the induction of these behaviors. Neurons likely communicate with *Hoxb8* microglia not only chemically (through ligand/receptor interactions) but also electrically (i.e., for example from sensory signals emanating from the somatosensory cortex) by generating inward currents that would depolarize the *Hoxb8* microglia membrane and thereby trigger the induction of anxiety and grooming. To us, the second option appears to be much more likely and is testable.

To simplify nomenclature, we recommend that in the future, designation of non-Hoxb8 and Hoxb8 microglia as A- and B-microglia respectively, in keeping with their time of entry into the brain parenchyma.

## Supplementary information

Supplementary Figure 1

Supplementary Figure 2

Supplementary Figure 3

Supplementary Figure 4

Supplementary Figure 5

Supplementary Figure 6

Supplementary Figure 7

Supplementary Figure 8

Supplementary Figure 9

Supplementary Figure 10

Supplementary Figure 11

Supplementary Figure 12

Supplementary Figure 13

Supplementary Figure 14

Supplementary Figure 15

Supplementary Figure 16

Supplementary Figure 17

Supplementary Figure 18

Supplementary Figure 19

Supplementary Figure 20

Supplementary Figure 21

Supplementary Figure 22

Supplementary Figure 23

Supplementary Figure 24

Supplementary Figure 25

Supplementary Figure Legends
