# Supplementary figures and images for: Optogenetic stimulation of mouse Hoxb8 microglia in specific regions of the brain induces anxiety, grooming, or both
Source: Mol Psychiatry. 2023 Apr 10;29(6):1726–40. doi: 10.1038/s41380-023-02019-w (PMC11371632; doi:10.1038/s41380-023-02019-w)

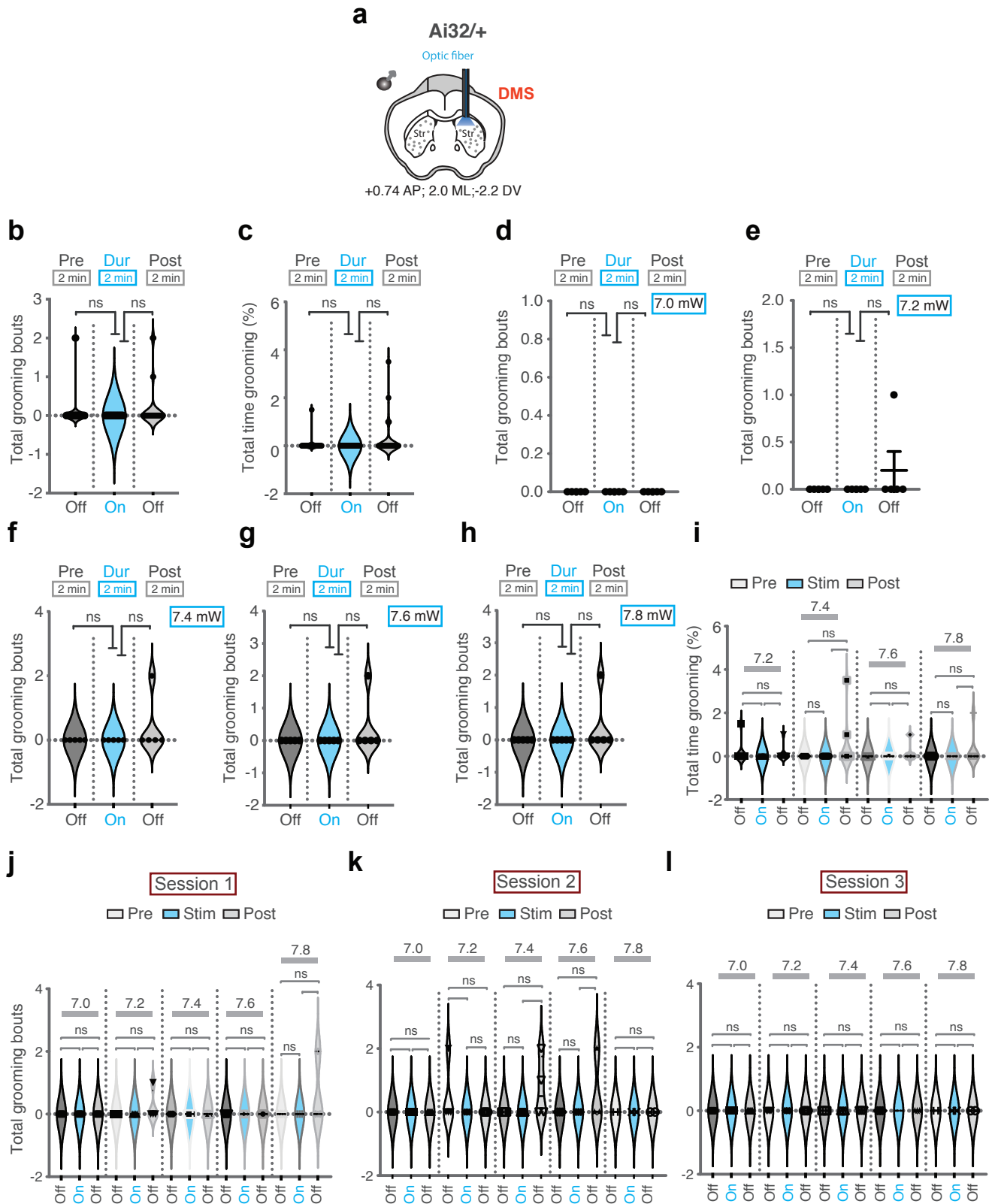

Supplement: Supplementary file 1 — Supplementary Figure 1 [file 41380_2023_2019_MOESM1_ESM.pdf]

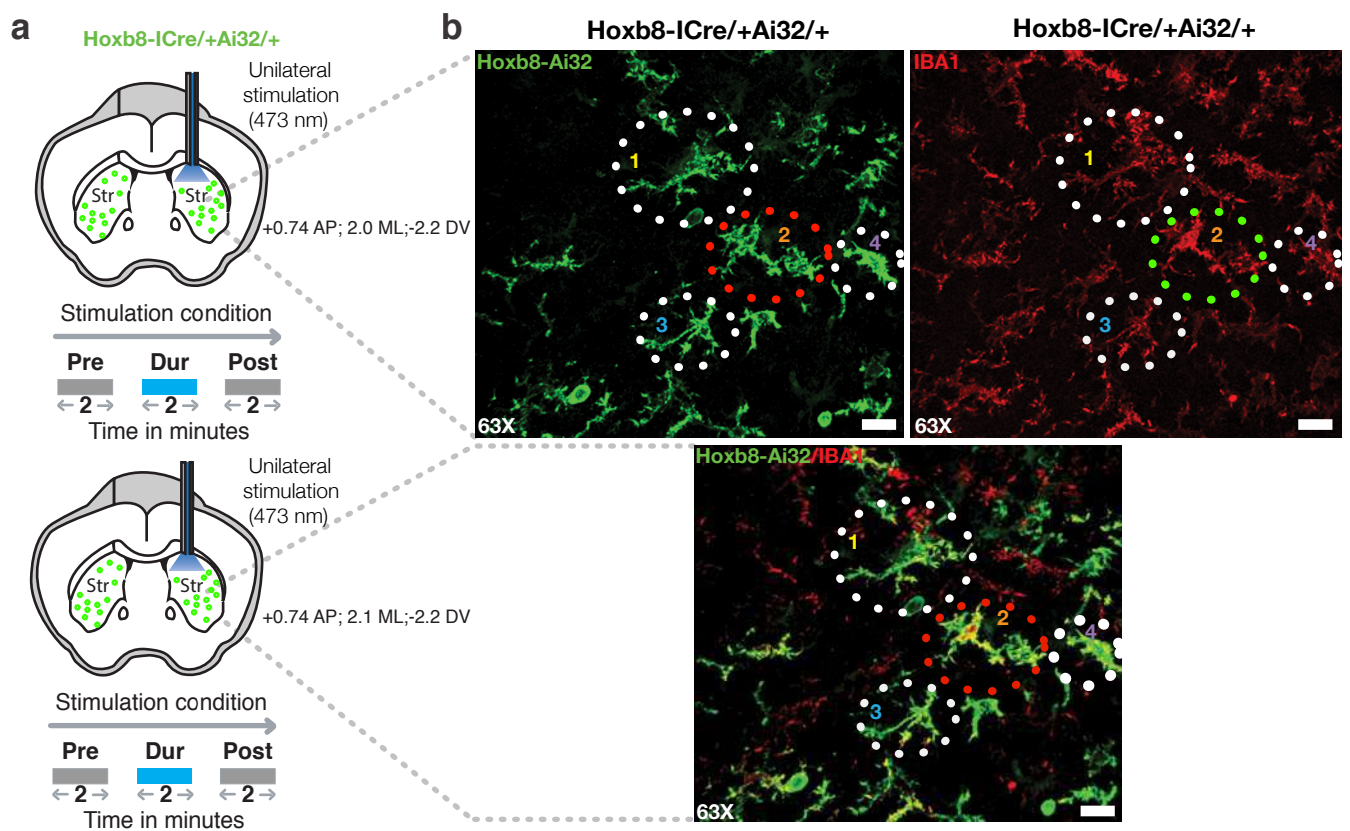

Supplement: Supplementary file 2 — Supplementary Figure 2 [file 41380_2023_2019_MOESM2_ESM.pdf]

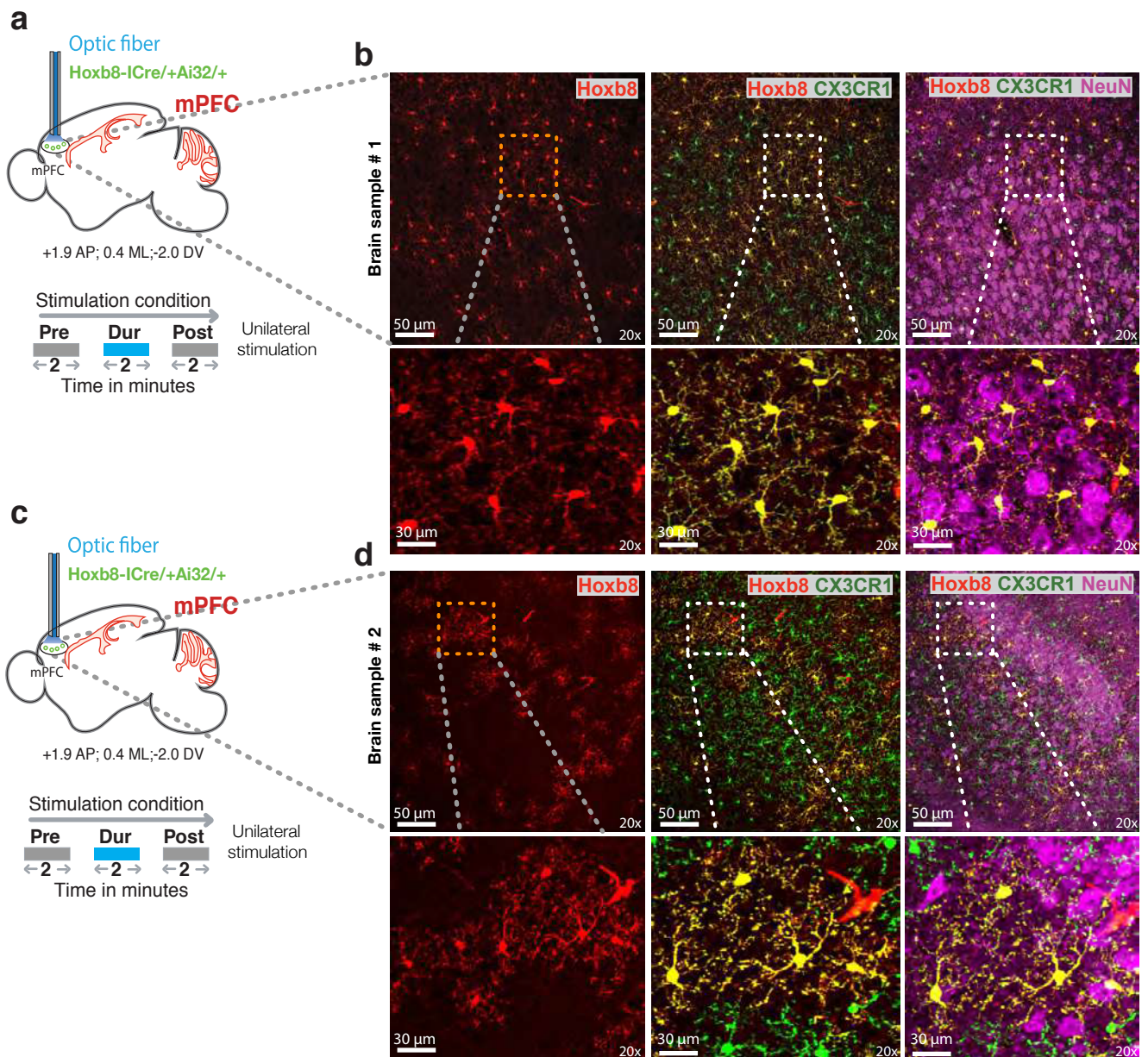

Supplement: Supplementary file 3 — Supplementary Figure 3 [file 41380_2023_2019_MOESM3_ESM.pdf]

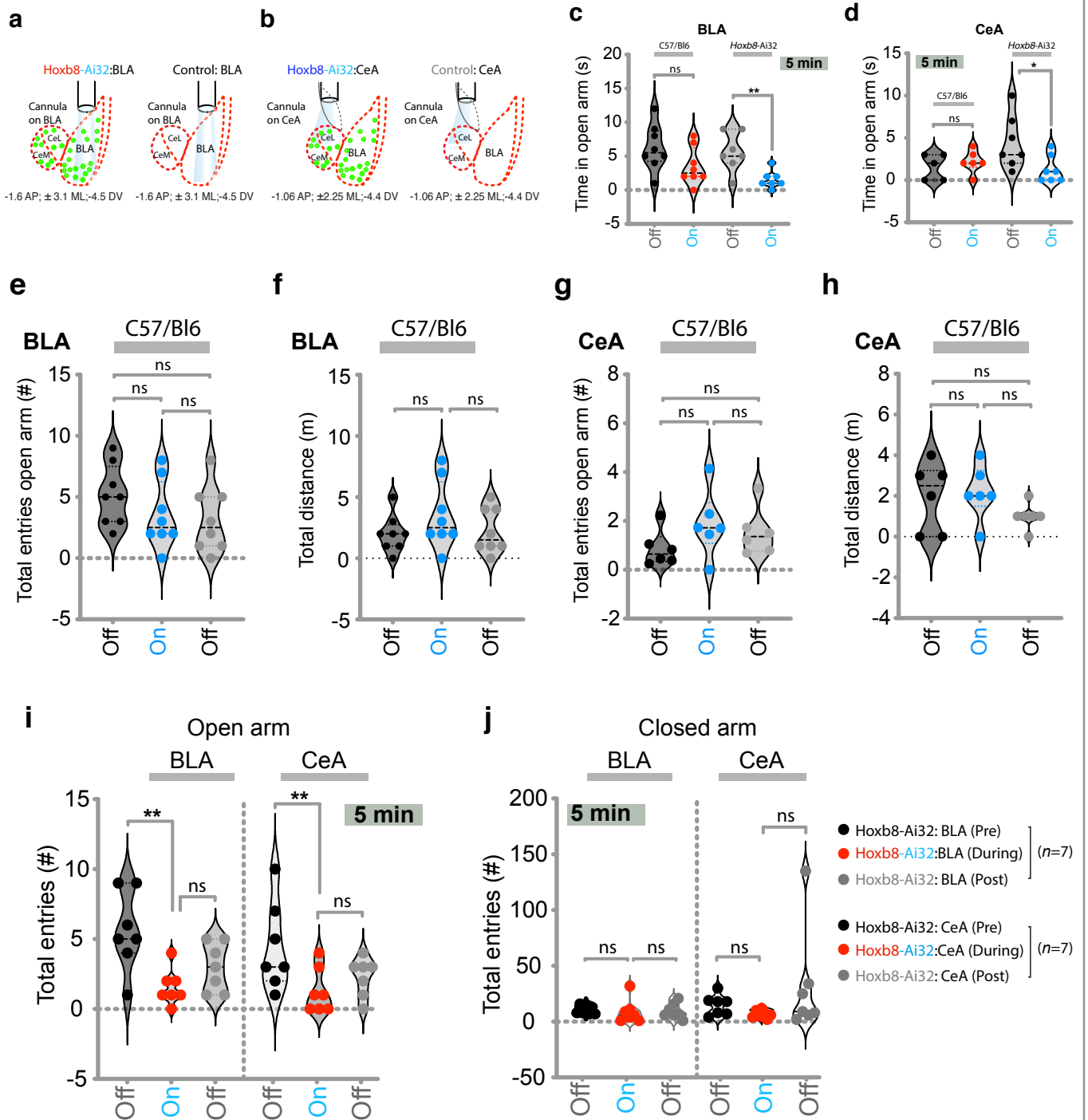

Supplement: Supplementary file 4 — Supplementary Figure 4 [file 41380_2023_2019_MOESM4_ESM.pdf]

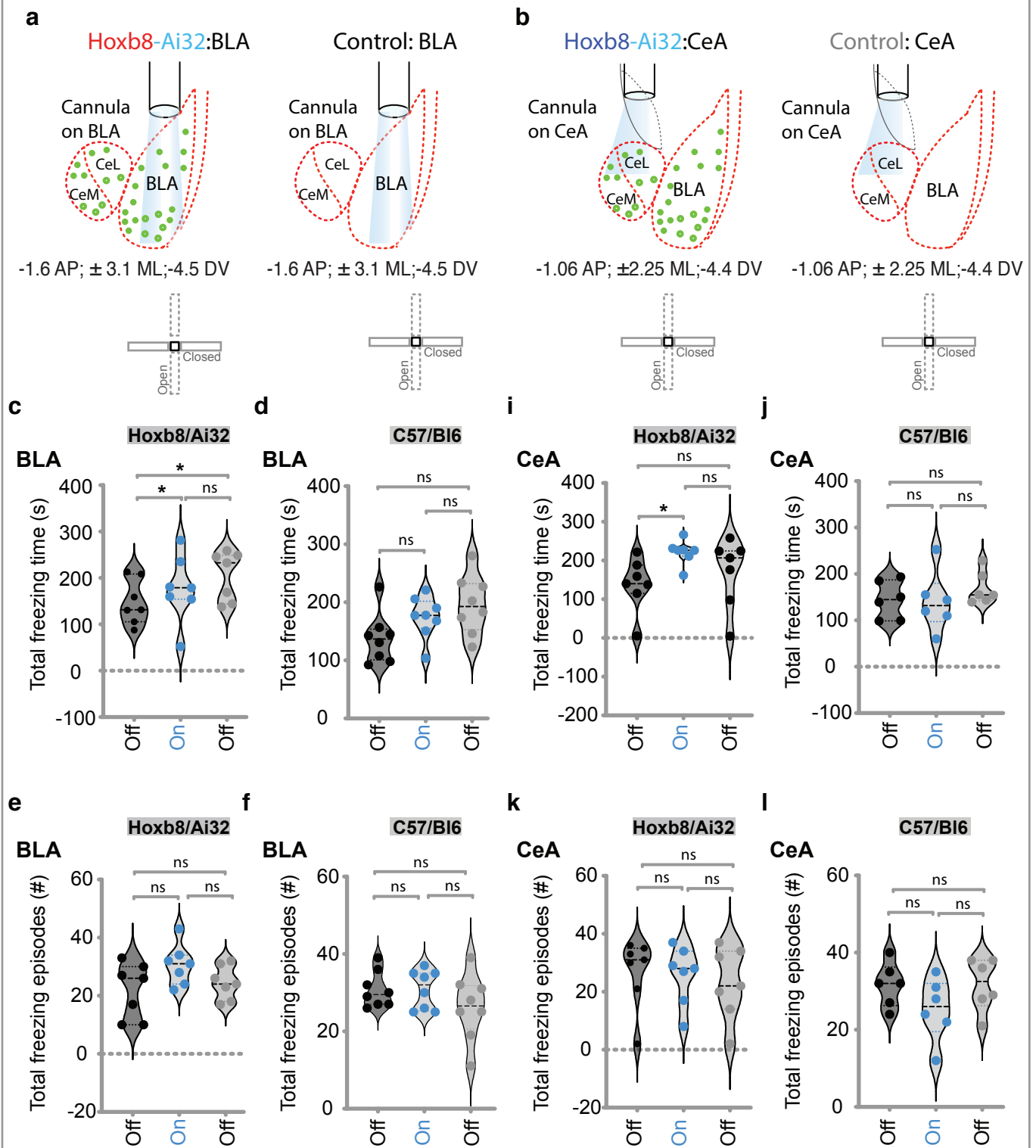

Supplement: Supplementary file 5 — Supplementary Figure 5 [file 41380_2023_2019_MOESM5_ESM.pdf]

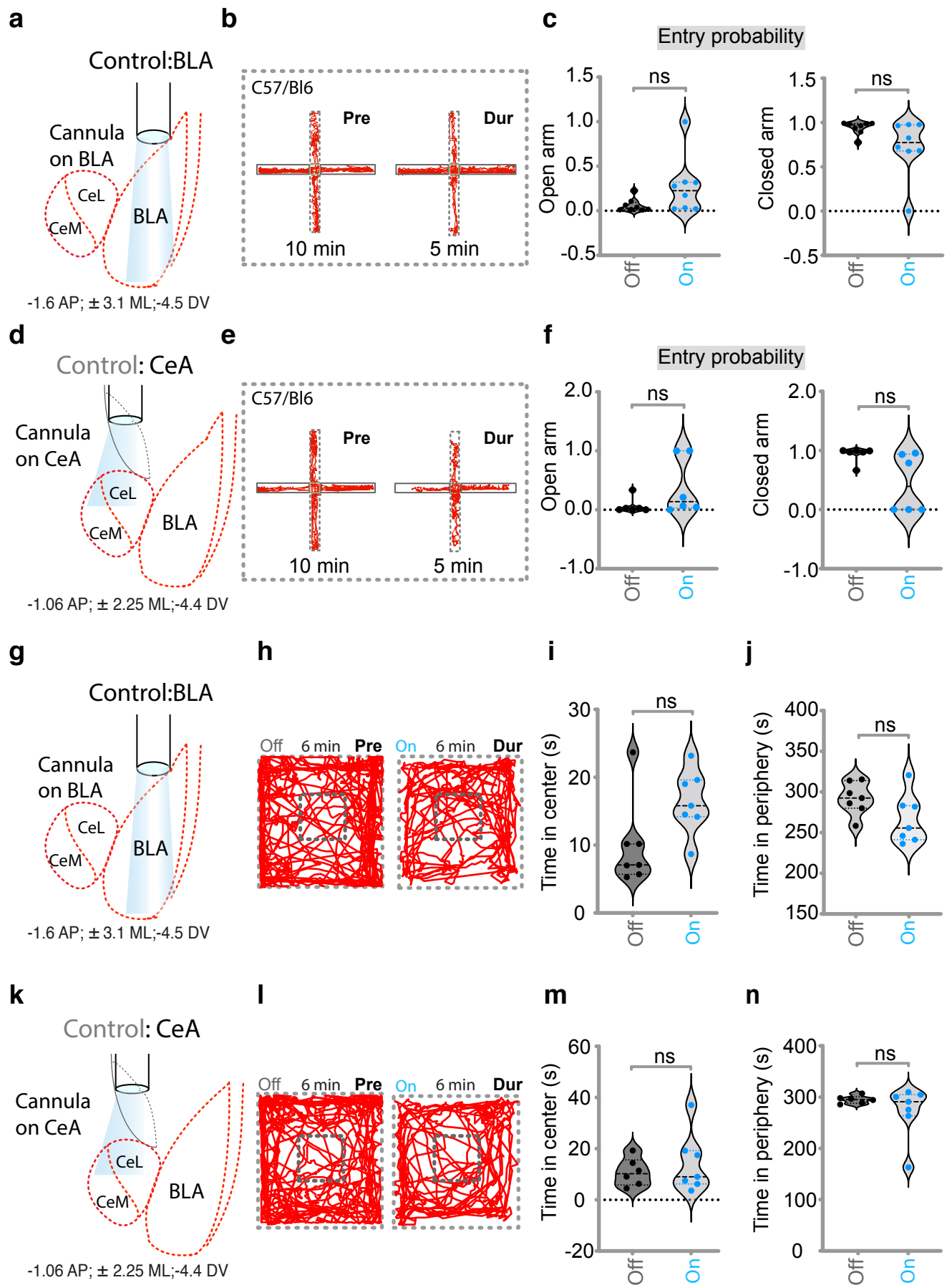

Supplement: Supplementary file 6 — Supplementary Figure 6 [file 41380_2023_2019_MOESM6_ESM.pdf]

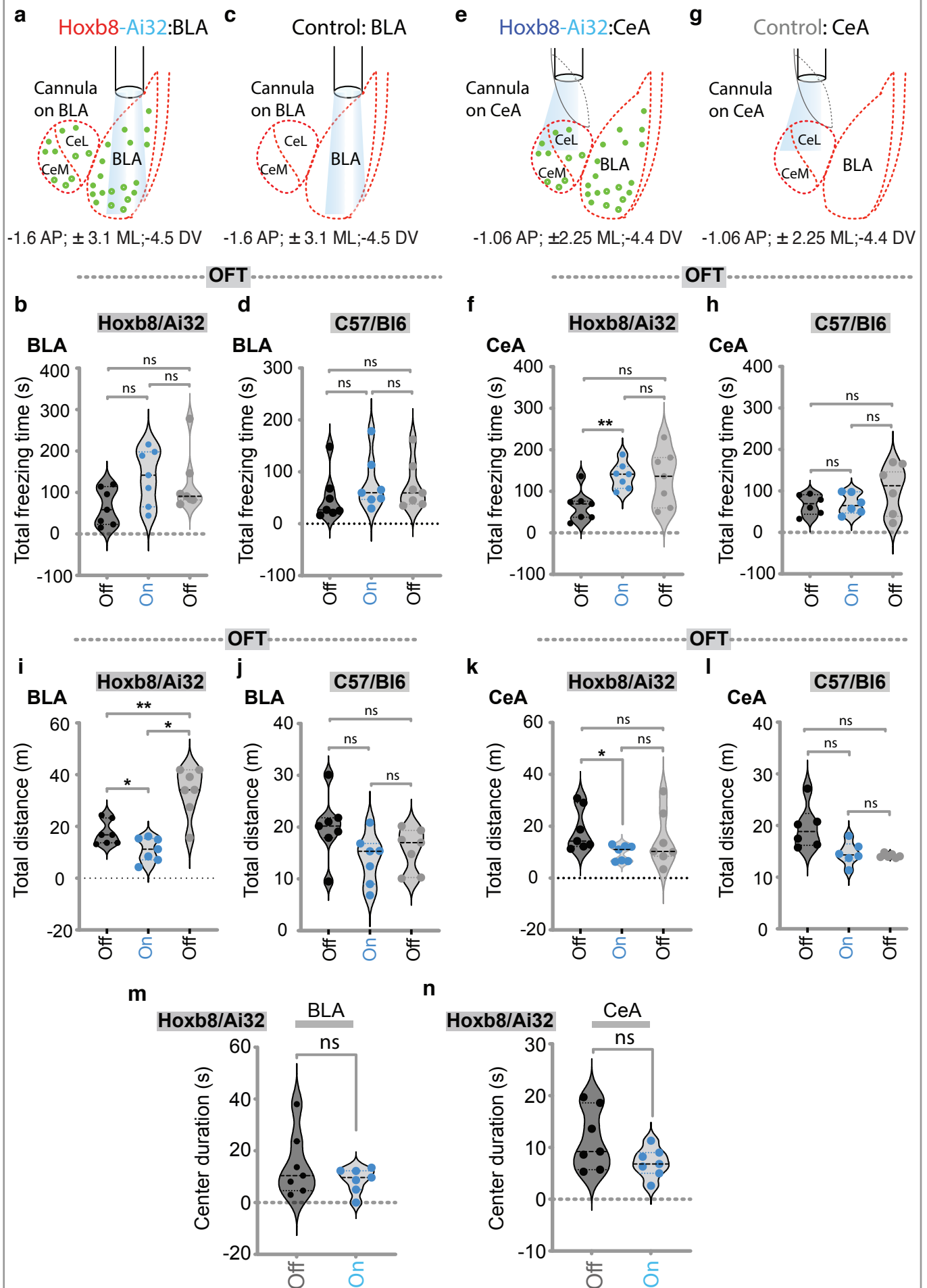

Supplement: Supplementary file 7 — Supplementary Figure 7 [file 41380_2023_2019_MOESM7_ESM.pdf]

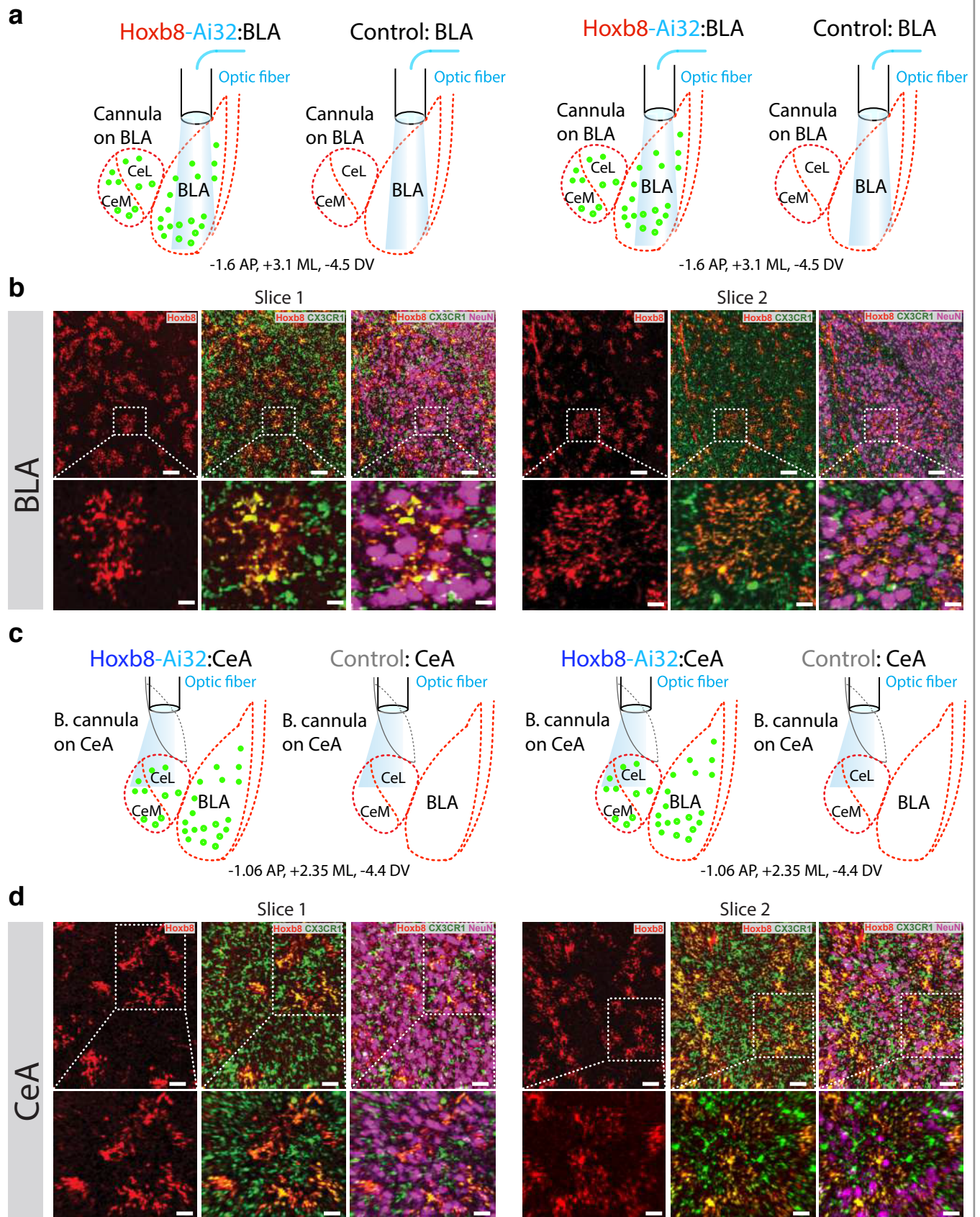

Supplement: Supplementary file 8 — Supplementary Figure 8 [file 41380_2023_2019_MOESM8_ESM.pdf]

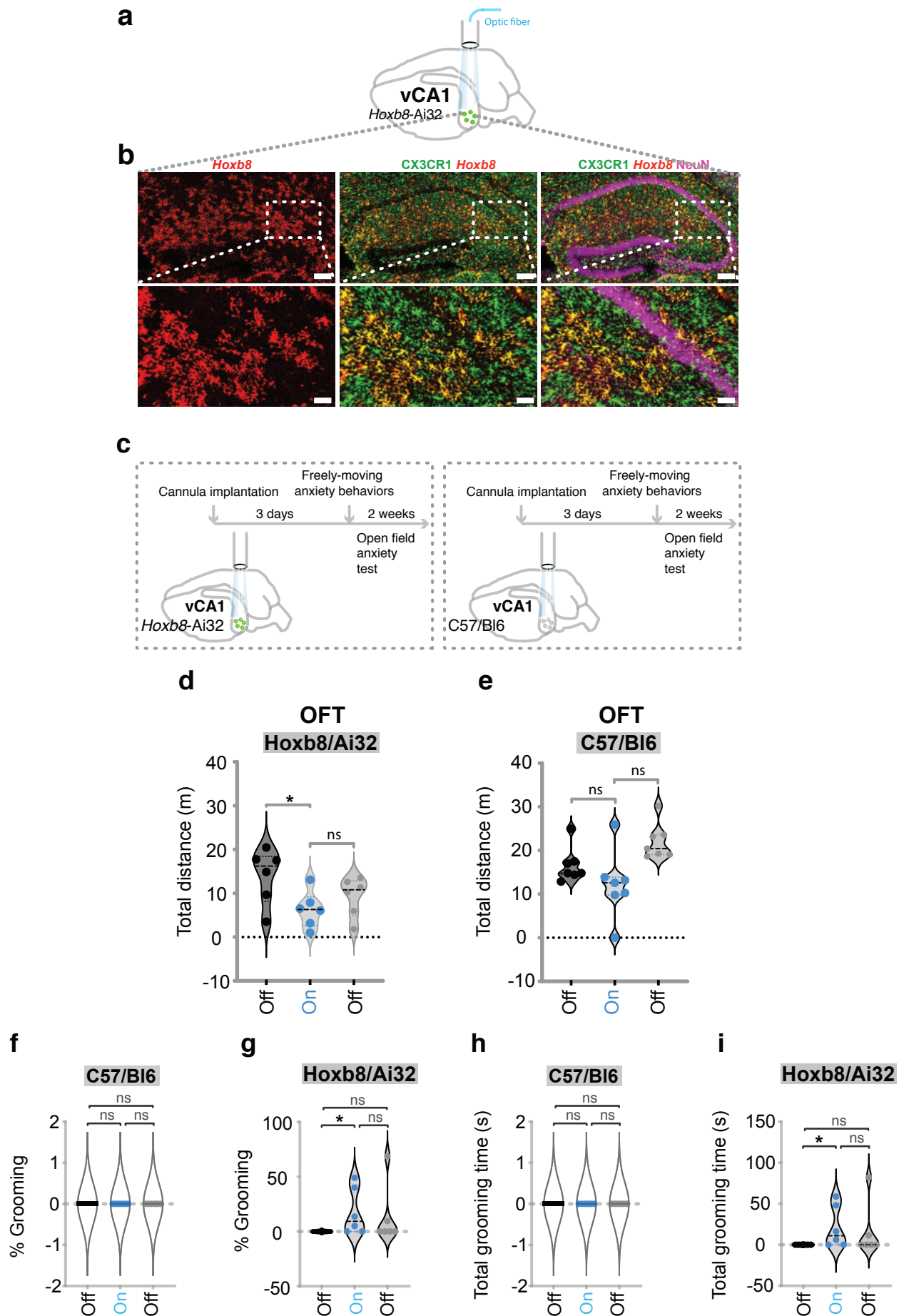

Supplement: Supplementary file 9 — Supplementary Figure 9 [file 41380_2023_2019_MOESM9_ESM.pdf]

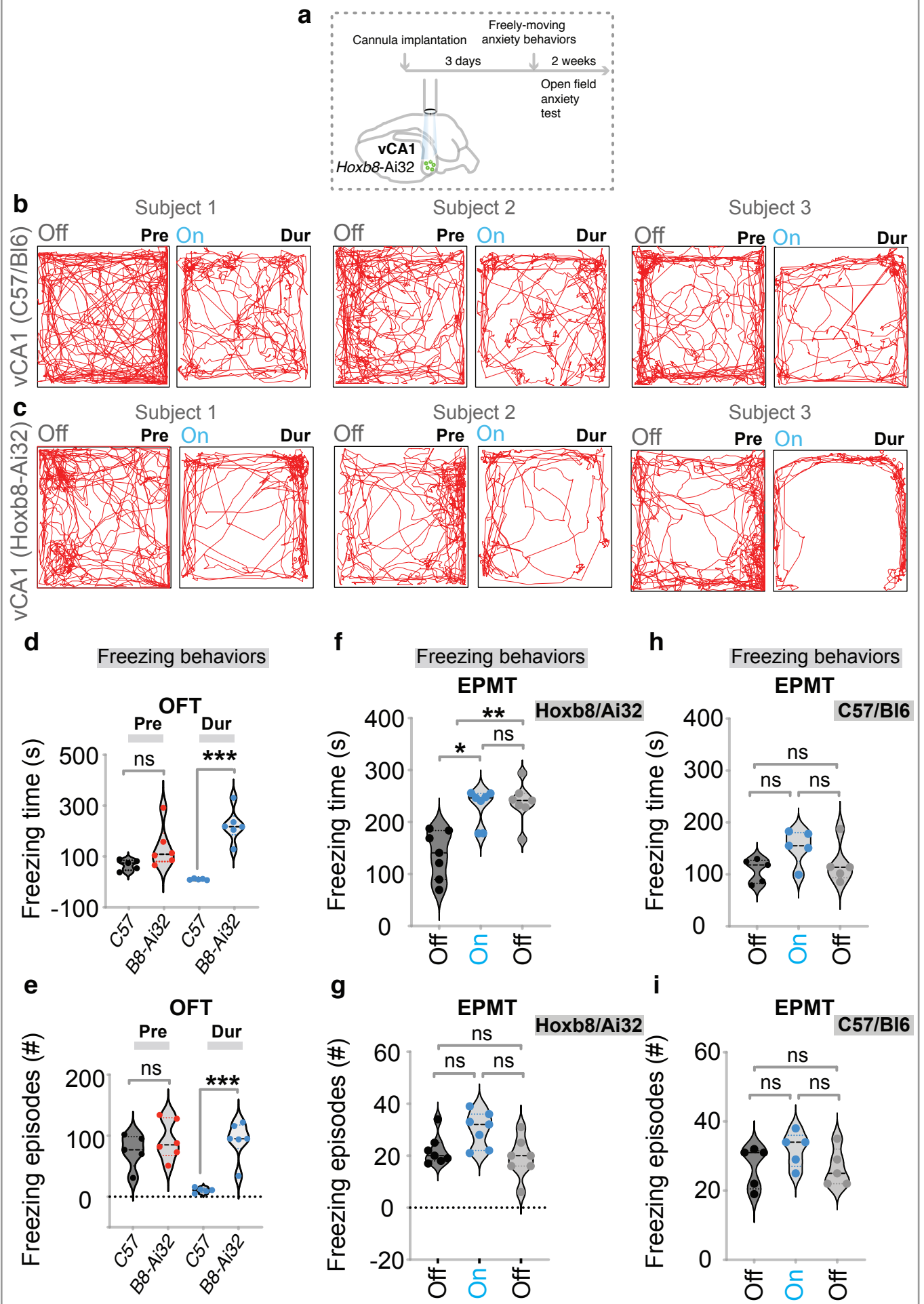

Supplement: Supplementary file 10 — Supplementary Figure 10 [file 41380_2023_2019_MOESM10_ESM.pdf]

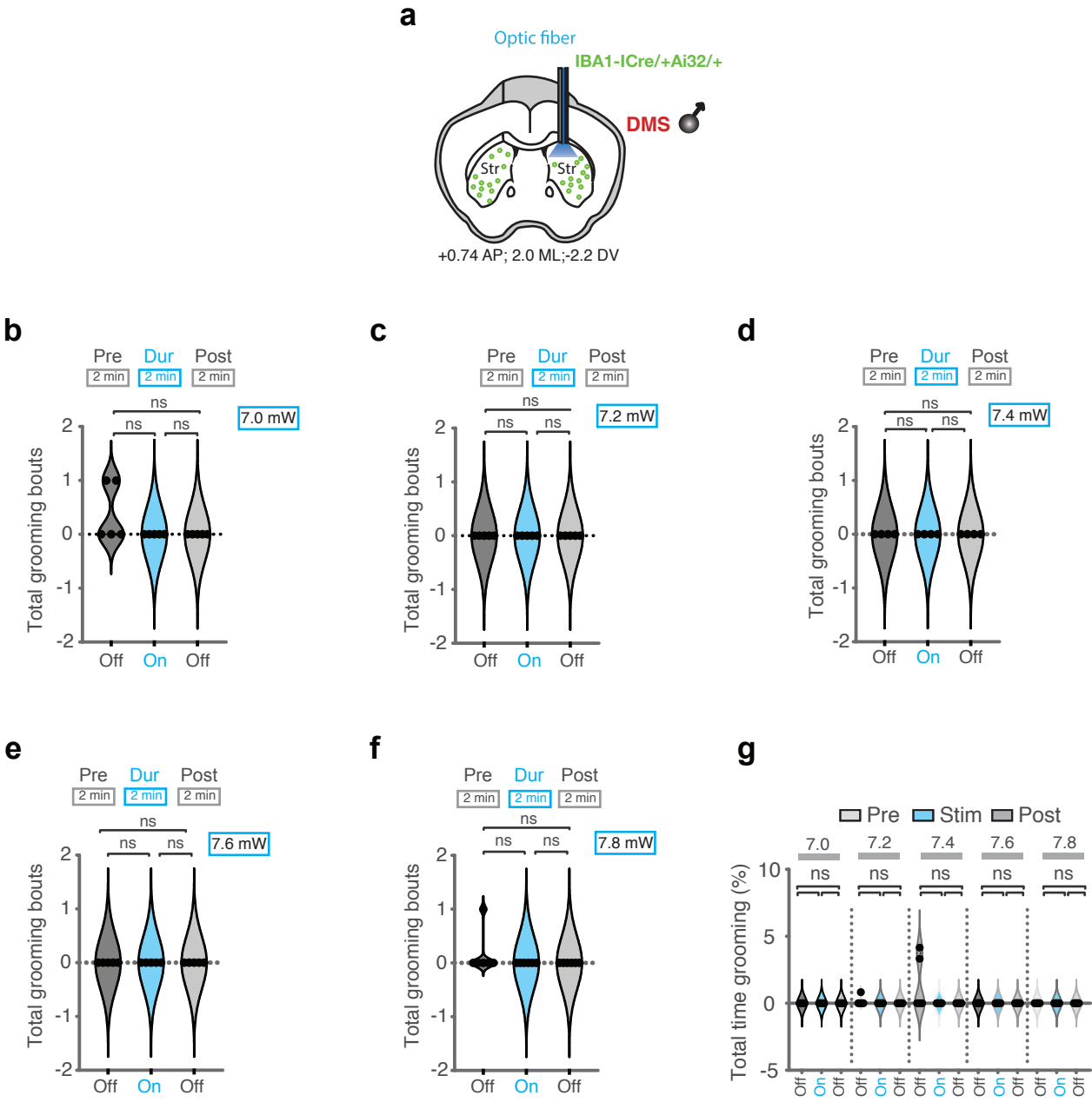

Supplement: Supplementary file 11 — Supplementary Figure 11 [file 41380_2023_2019_MOESM11_ESM.pdf]

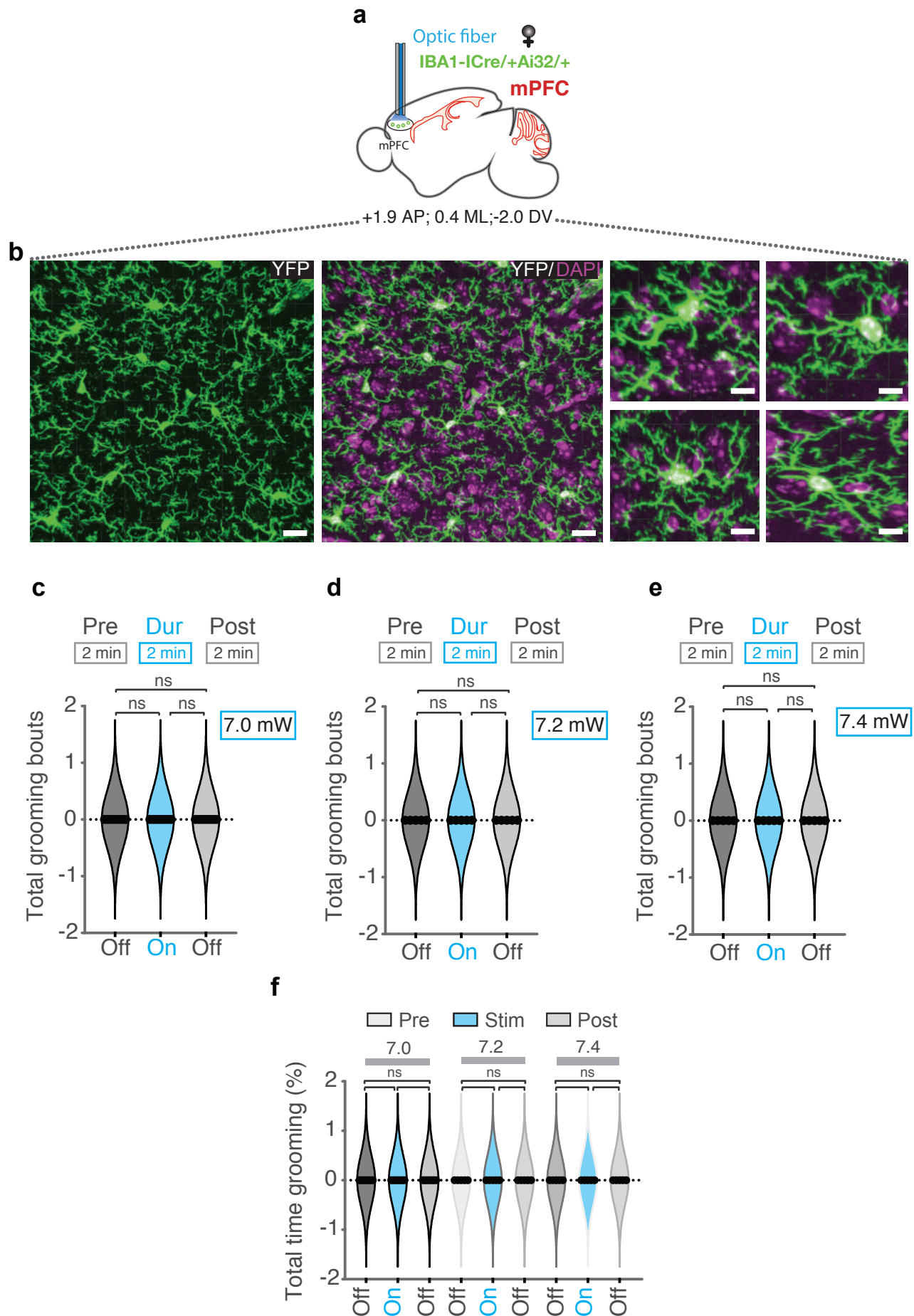

Supplement: Supplementary file 12 — Supplementary Figure 12 [file 41380_2023_2019_MOESM12_ESM.pdf]

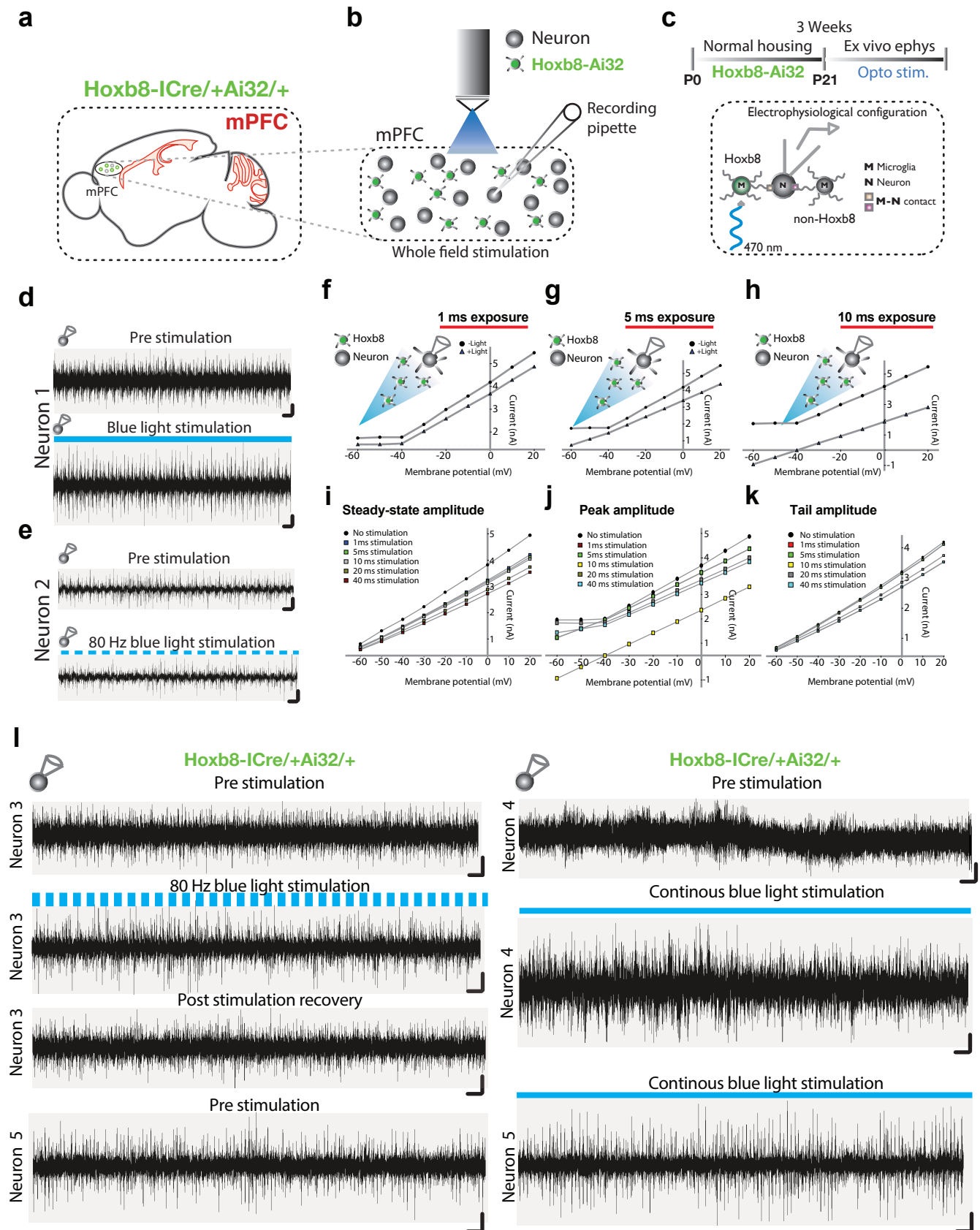

Supplement: Supplementary file 13 — Supplementary Figure 13 [file 41380_2023_2019_MOESM13_ESM.pdf]

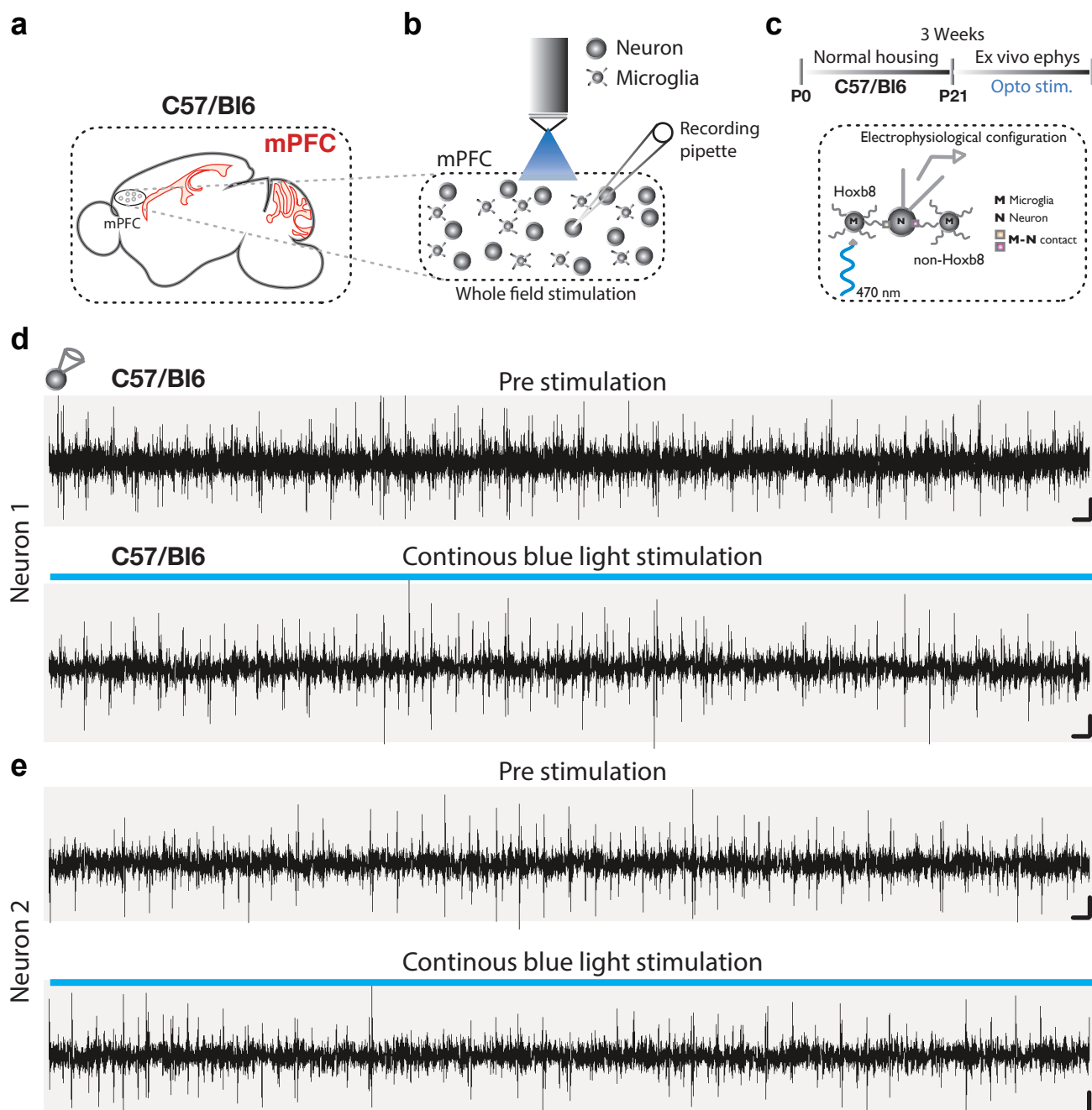

Supplement: Supplementary file 14 — Supplementary Figure 14 [file 41380_2023_2019_MOESM14_ESM.pdf]

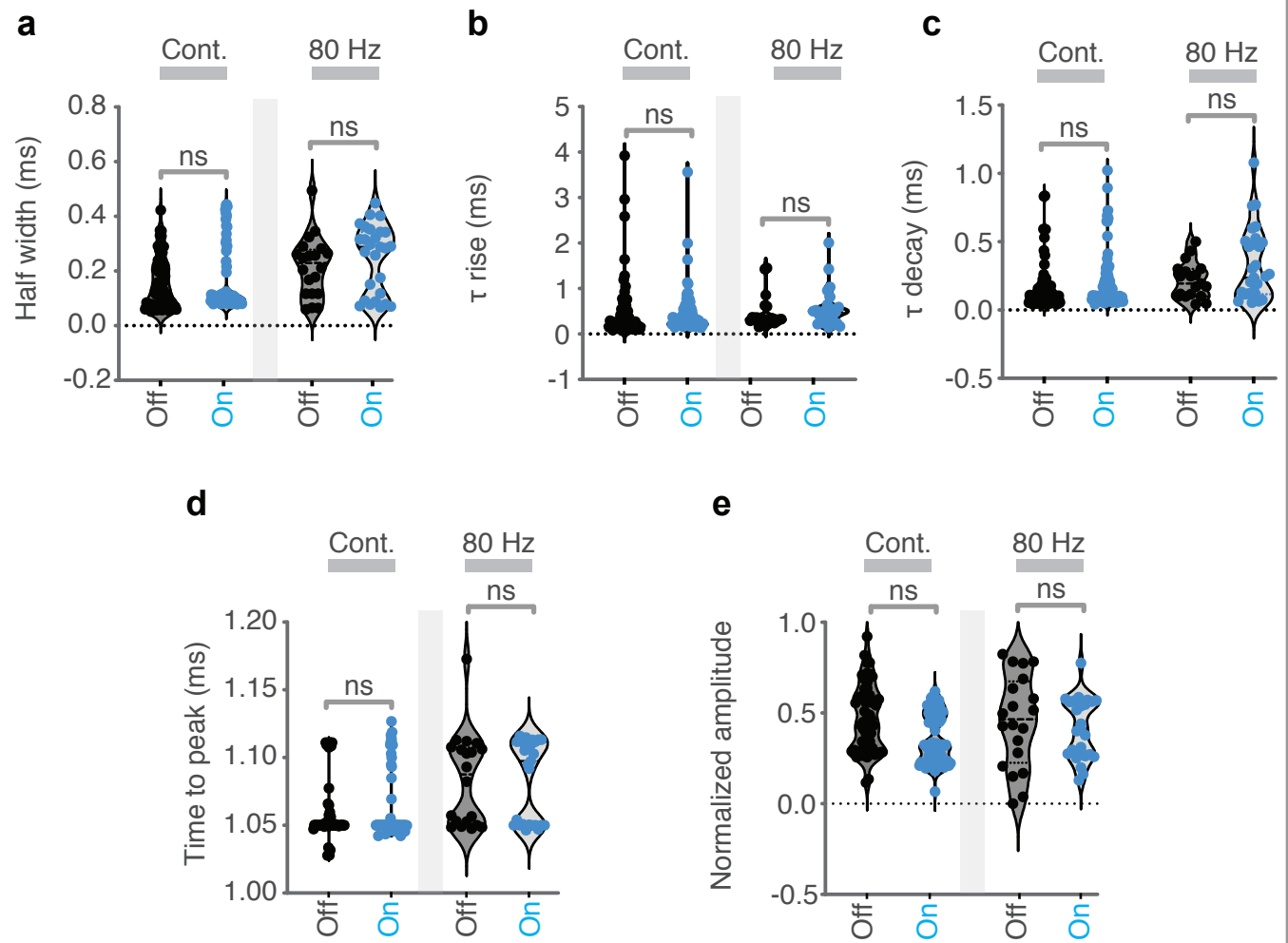

Supplement: Supplementary file 15 — Supplementary Figure 15 [file 41380_2023_2019_MOESM15_ESM.pdf]

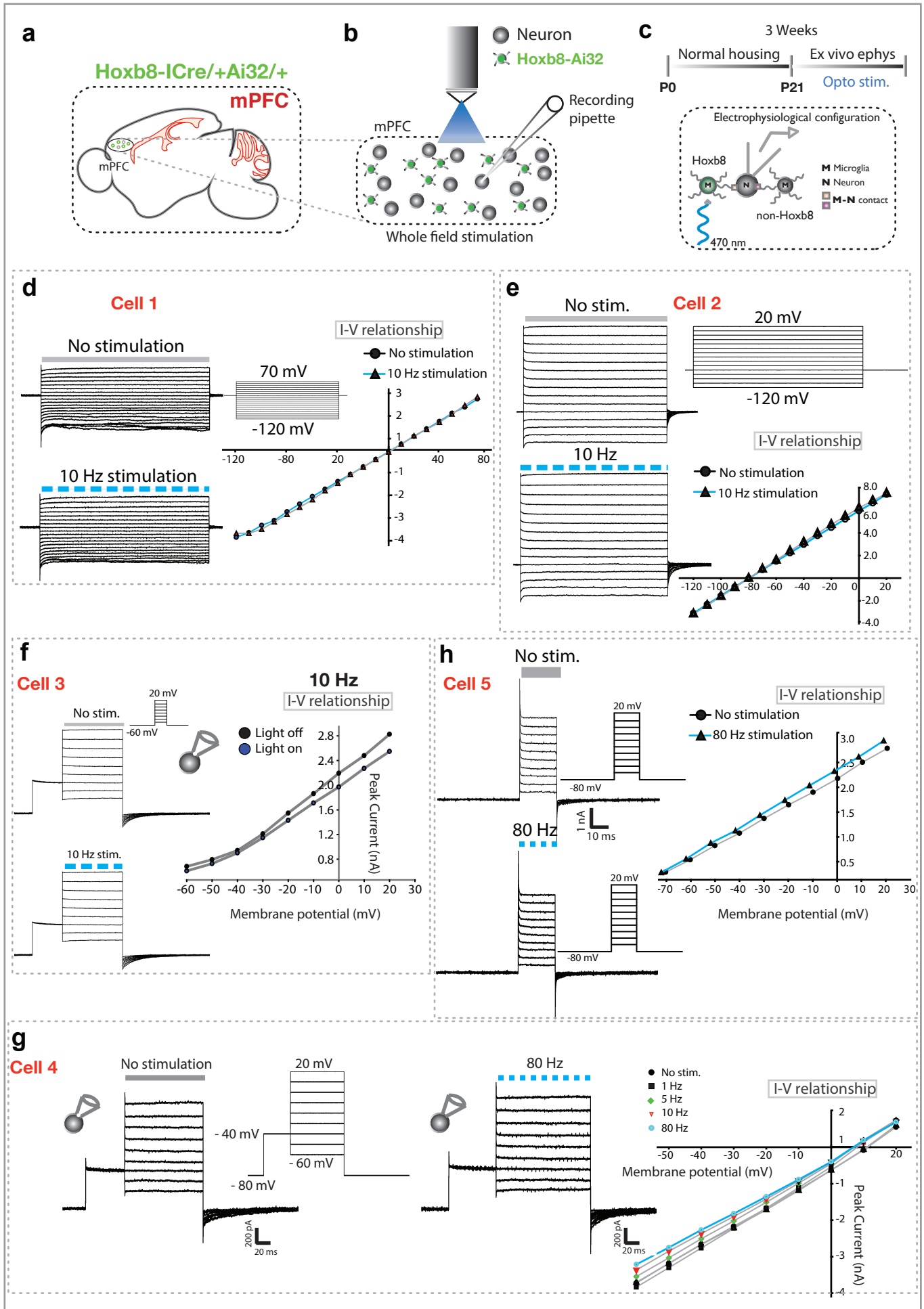

Supplement: Supplementary file 16 — Supplementary Figure 16 [file 41380_2023_2019_MOESM16_ESM.pdf]

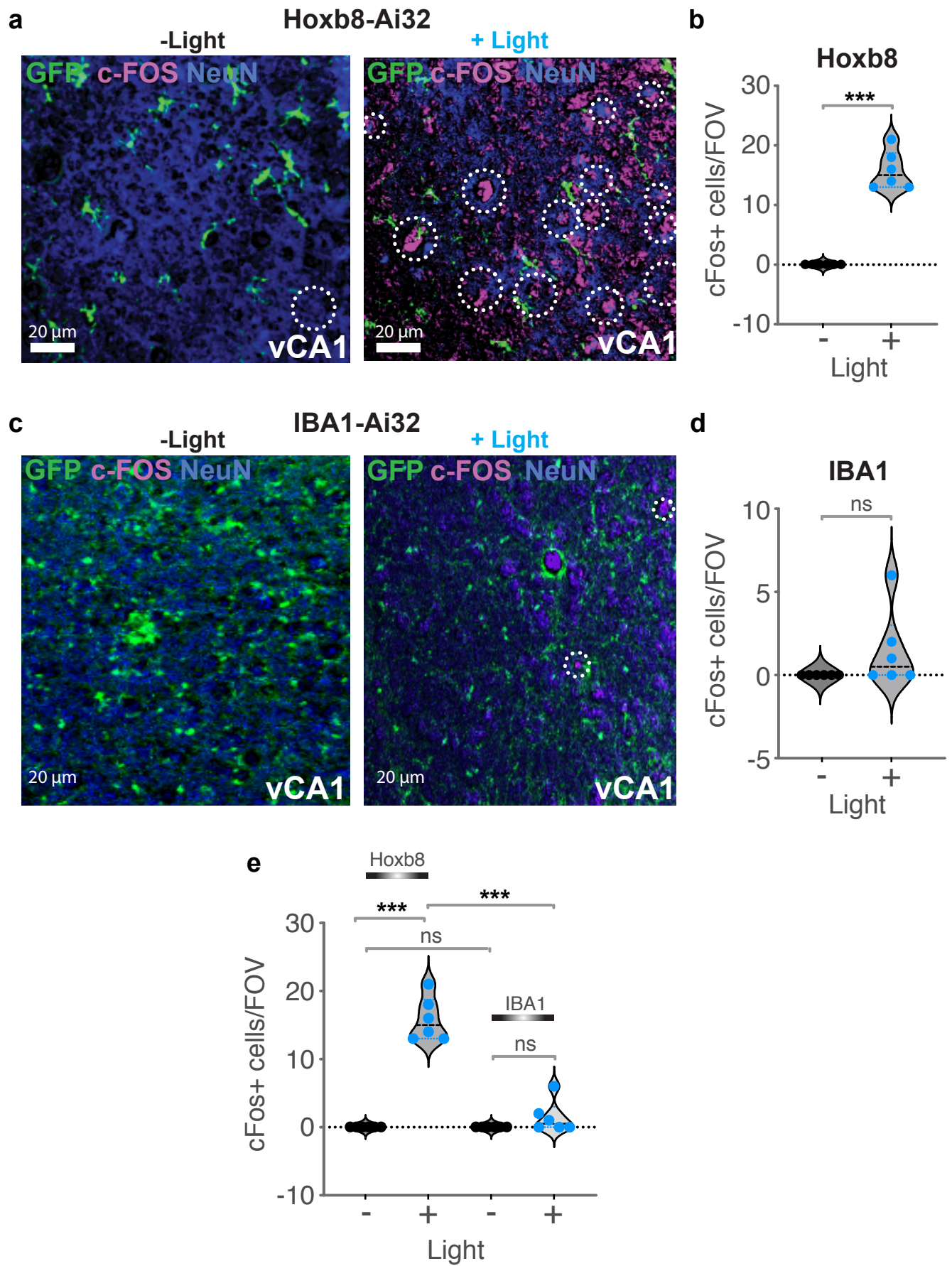

Supplement: Supplementary file 17 — Supplementary Figure 17 [file 41380_2023_2019_MOESM17_ESM.pdf]

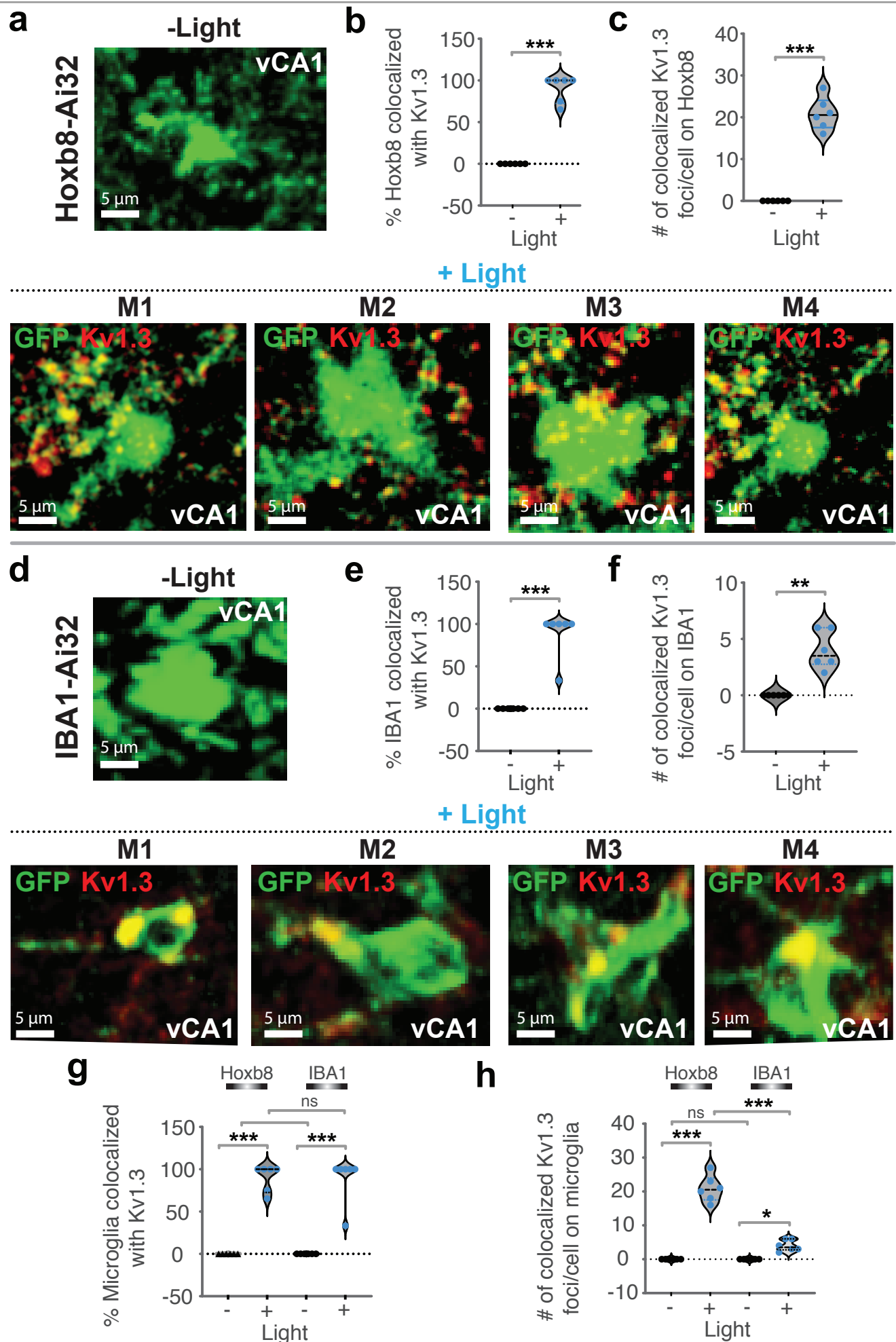

Supplement: Supplementary file 18 — Supplementary Figure 18 [file 41380_2023_2019_MOESM18_ESM.pdf]

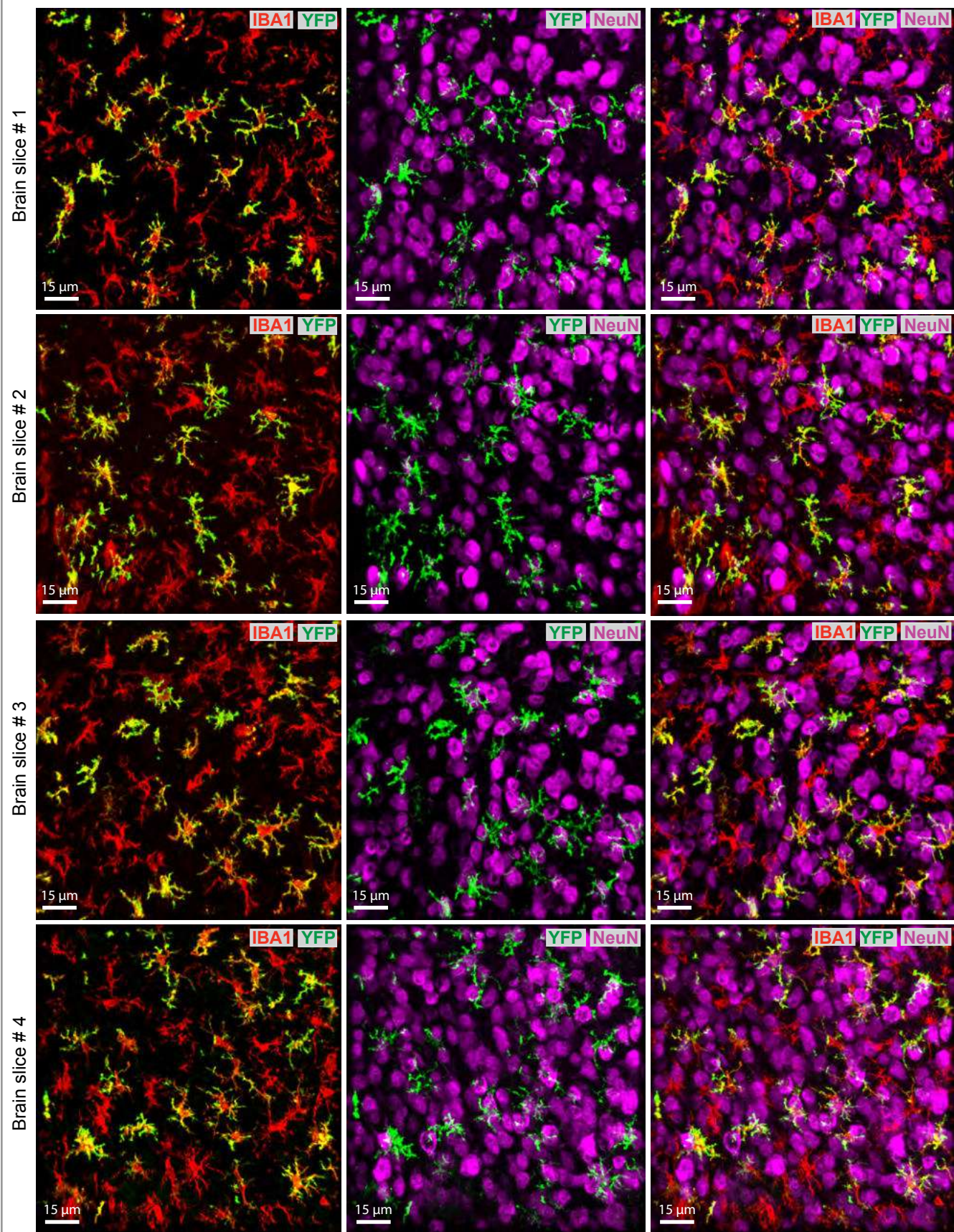

Supplement: Supplementary file 19 — Supplementary Figure 19 [file 41380_2023_2019_MOESM19_ESM.pdf]

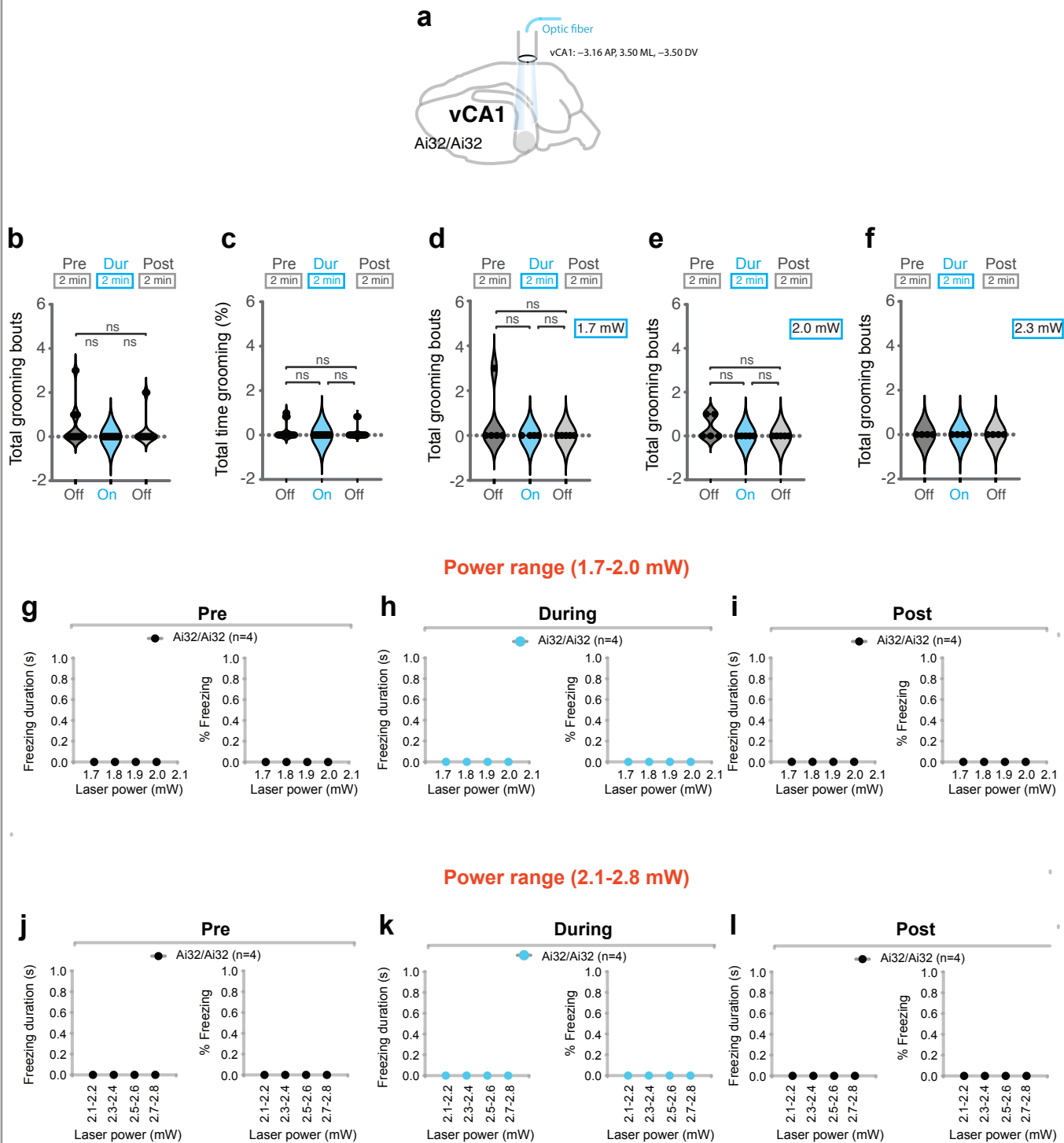

Supplement: Supplementary file 20 — Supplementary Figure 20 [file 41380_2023_2019_MOESM20_ESM.pdf]

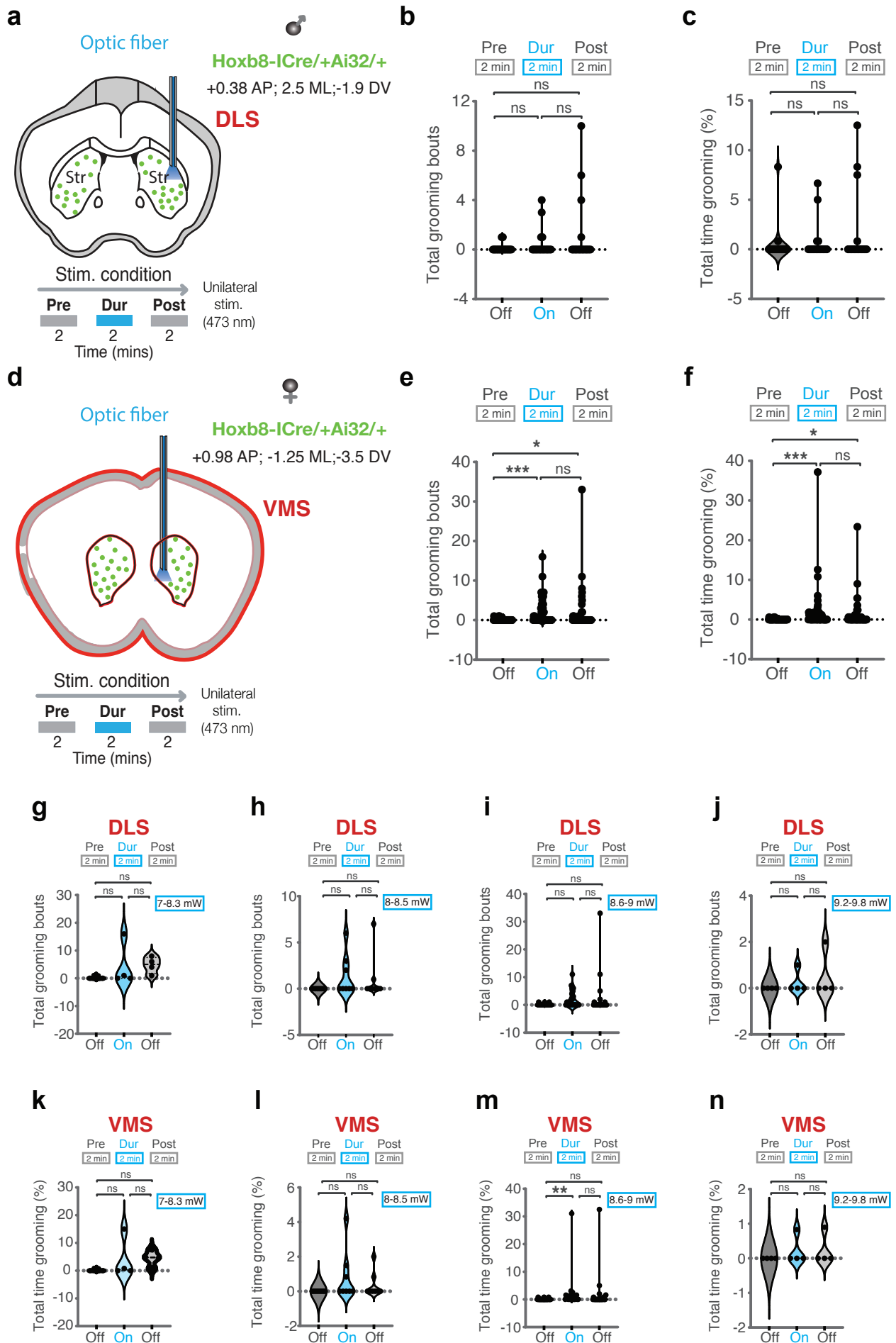

Supplement: Supplementary file 21 — Supplementary Figure 21 [file 41380_2023_2019_MOESM21_ESM.pdf]

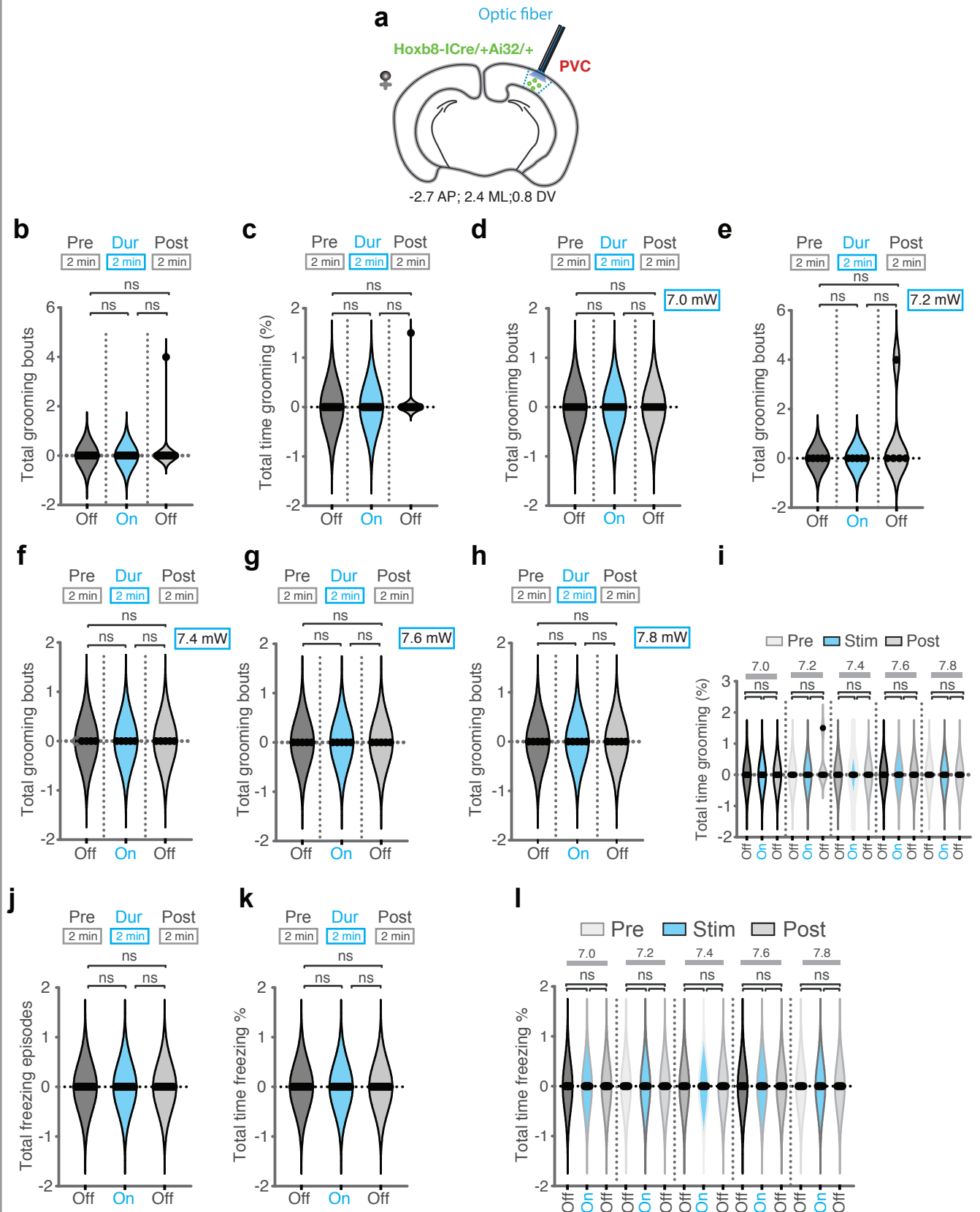

Supplement: Supplementary file 22 — Supplementary Figure 22 [file 41380_2023_2019_MOESM22_ESM.pdf]

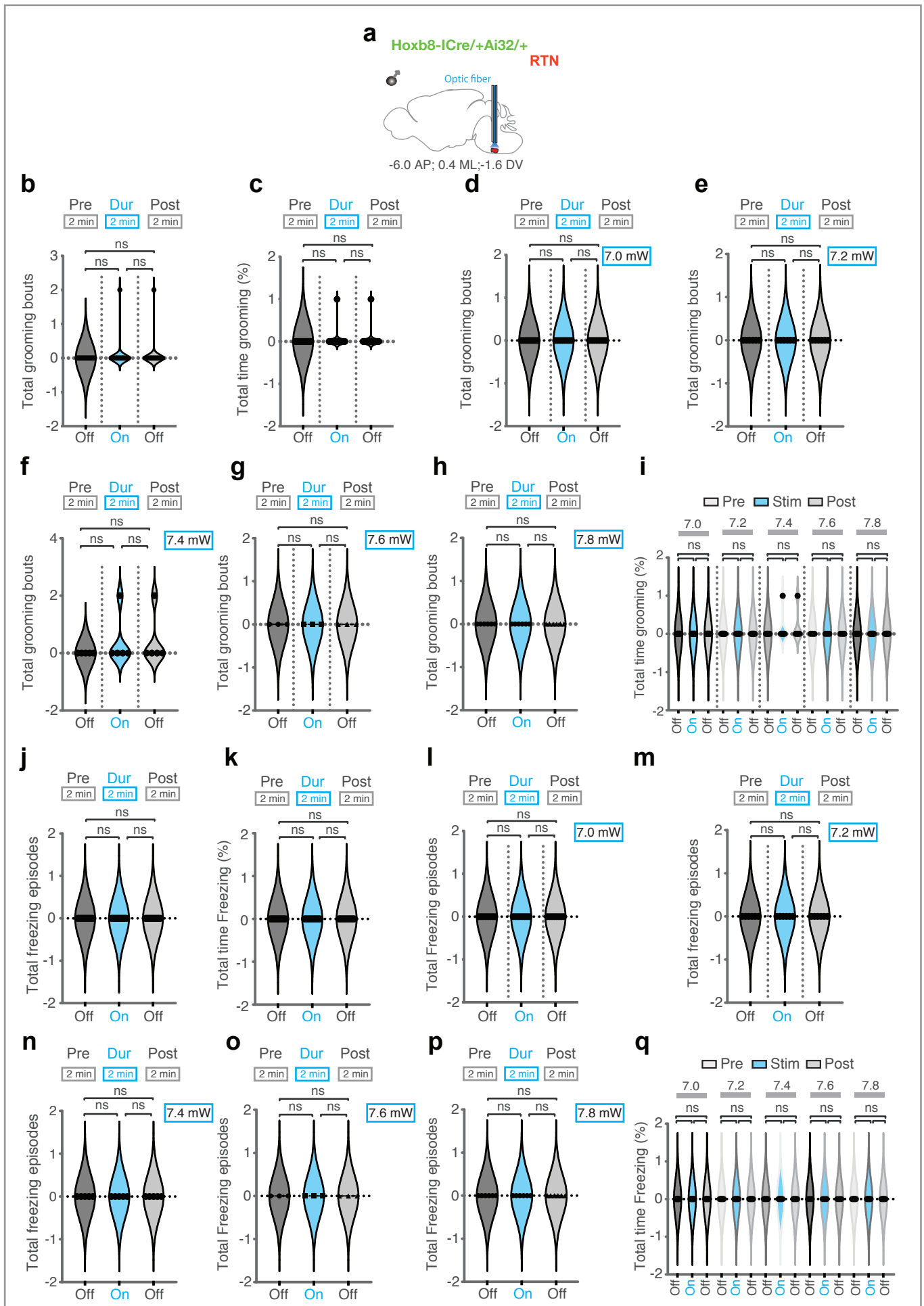

Supplement: Supplementary file 23 — Supplementary Figure 23 [file 41380_2023_2019_MOESM23_ESM.pdf]

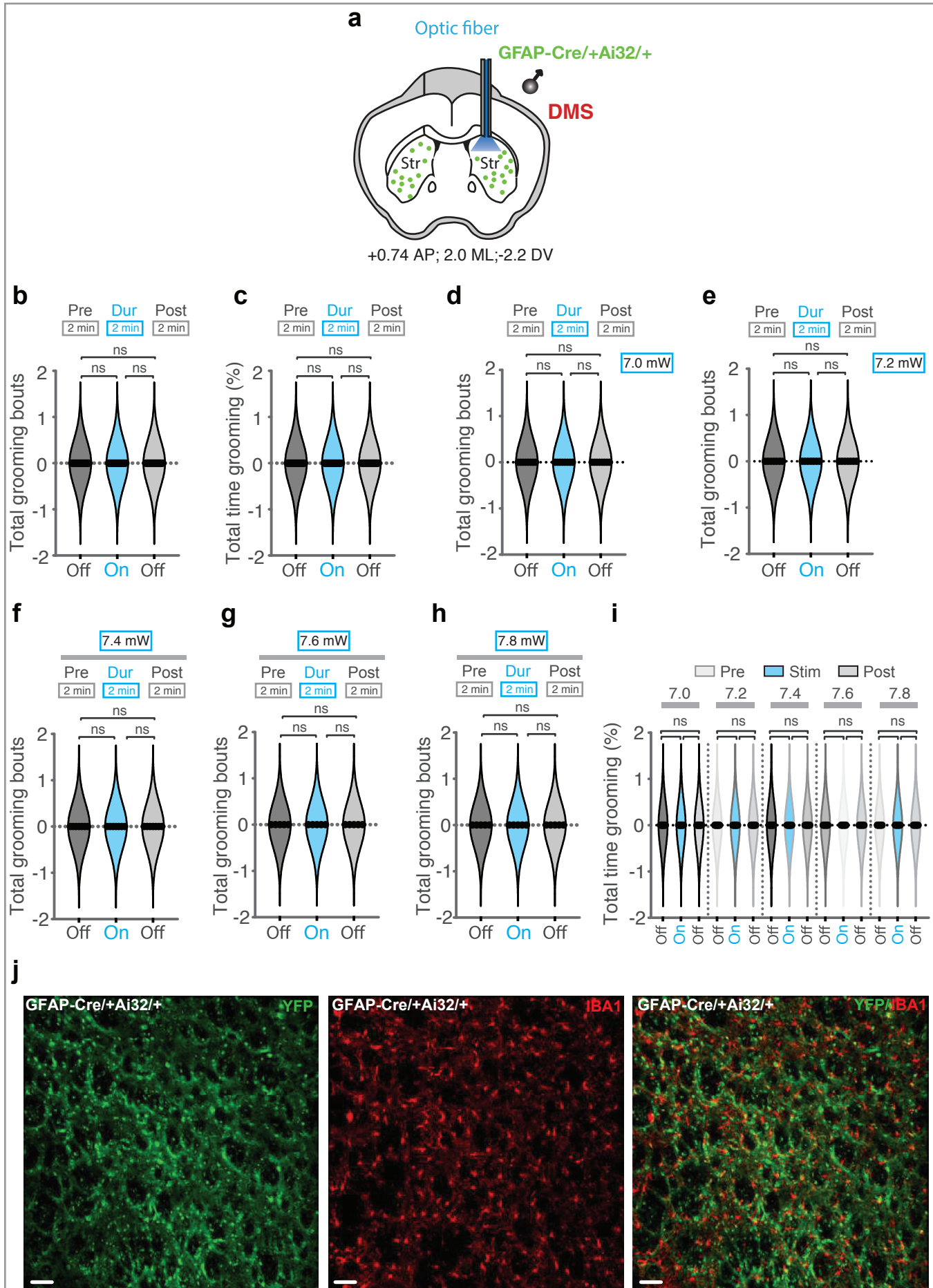

Supplement: Supplementary file 24 — Supplementary Figure 24 [file 41380_2023_2019_MOESM24_ESM.pdf]

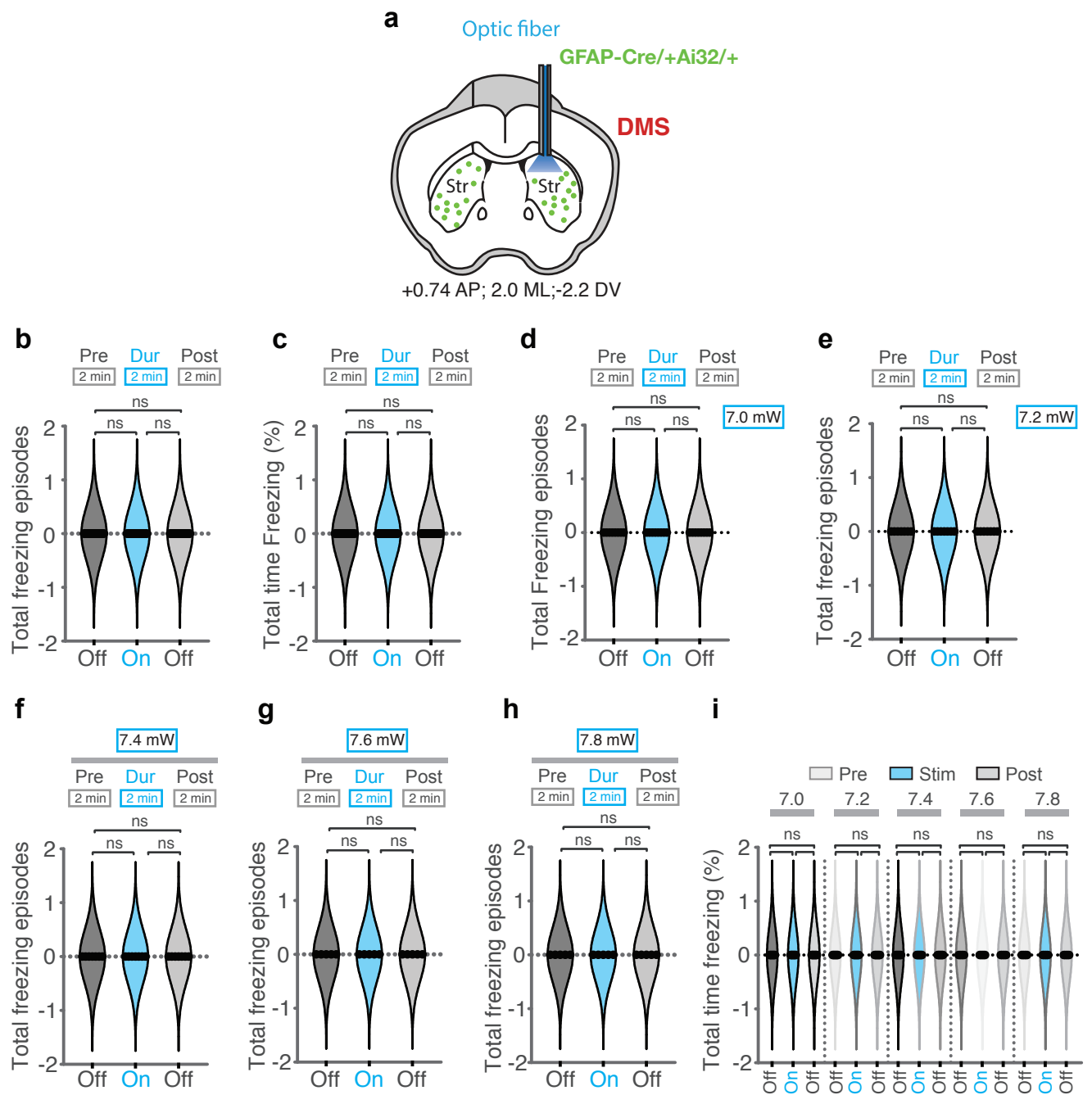

Supplement: Supplementary file 25 — Supplementary Figure 25 [file 41380_2023_2019_MOESM25_ESM.pdf]
