# Supplementary material for: Optogenetic stimulation of mouse Hoxb8 microglia in specific regions of the brain induces anxiety, grooming, or both
Source: Mol Psychiatry. 2023 Apr 10;29(6):1726–40. doi: 10.1038/s41380-023-02019-w (PMC11371632; doi:10.1038/s41380-023-02019-w)
Supplement: Supplementary file 26 — Supplementary Figure Legends [file 41380_2023_2019_MOESM26_ESM.pdf]

Supplementary Figure 1

Optogenetic stimulation of *Ai32/+* mice, lacking *Hoxb8*-$^{\text{IRES-Cre}}$, within DMS fails to induce grooming.

(**a**). Representation of cannula insertion and light (473 nm) delivery into the DMS of Ai32$^{/+}$ male mice lacking *Hoxb8*^{IRESCre}. Shown in the picture (a) is a straight cannula that was stereotaxically inserted within DMS in the right brain hemisphere at the coordinates +0.74 AP, +2.0 ML, -2.2 DV. Stimulation condition consisted of a two-minute baseline recording followed by 2 minutes of continuous optogenetic stimulation at 473 nm and 2 minutes of post stimulation recording. (**b, c).** No significant change in the total number of grooming bouts (b) or the percentage of grooming time (c) in response to DMS laser stimulation. (**d-h**). Representation of data compiled from pre, during and post stimulation conditions showing no significant change in the total number of grooming bouts (d-h) or the percentage of grooming time (i) in Ai32$^{/+}$ DMS implanted mice for the laser power ranges 7.0 mW (d), 7.2 mW (e), 7.4 mW (f), 7.6 mW (g) and 7.8 mW (h) for the total grooming bouts and similar power range and comparison for the percentage of time grooming (i). (**j-l**). Total grooming bouts was not altered significantly in response to 2 minute continuous optogenetic stimulation in three independent sessions (j, k, l). Representation of data compiled from pre, during and post stimulation conditions showing no significant change in the total number of grooming bouts (j-l) at laser power ranges 7.0 mW, 7.2 mW, 7.4 mW, 7.6 mW and 7.8 mW for the total grooming bouts. ns, not significant. Repeated measure One-way ANOVA with Geiser greenhouse correction and Tukey's HSD posthoc multiple comparison tests were used for b-l. 3-4 months old mice were used for the cannula implantation within DMS. n=5 mice.

Supplementary Figure 2

***Hoxb8* microglia preserves normal distribution and morphological features following optogenetic stimulation.**

(**a**). The picture shows the site of implantation of cannula within the DMS of *Hoxb8*$^{IRESCre/+}$;Ai32$^{/+}$ mouse. The experimental paradigm is described below. Optogenetic stimulation at 473 nm was conducted for two minutes on DMS unilaterally on the right brain hemisphere at the stereotaxic location +0.74 AP; 2.1 ML, -2.2 DV and video recordings were made for a total of 6 minutes with 2 minutes of pre, during and post stimulation condition for measuring the effect of optogenetic stimulation of *Hoxb8* microglia on the behavioral output. (**b**). Confocal images of brain samples obtained from a representative *Hoxb8*-$^{IRES\text{-}Cre/+}$; Ai32$^{/+}$ mouse following implantation and optogenetic experiment for the histological verification of the site of the implantation and the presence of *Hoxb8* microglia below the cannula. High resolution (63X) images of the *Hoxb8* microglia (left panel) within DMS retaining morphological features following implantation and optogenetic experiment. Labeling of *Hoxb8*-lineage microglia with IBA1, a pan microglia marker (right panel). Bottom ~~left~~ panel represents the colocalization of *Hoxb8* microglia with IBA1. The numbers on the images indicate spatially distinct representation of *Hoxb8* microglia. Scale bar 10 μm.

Supplementary Figure 3

Distribution and presence of *Hoxb8* and non-*Hoxb8* microglia within mPFC.

(**a, c**)**.** The picture shows the site of implantation of cannula within the mPFC. The experimental paradigm is shown below. Optogenetic stimulation at 473 nm was conducted for 2 minutes on mPFC unilaterally on the right brain hemisphere at the stereotaxic location +1.9 AP, 0.4 ML, -2.0 DV and video recordings were made for a total of 6 minutes with 2 minutes of pre, during and post stimulation condition for measuring the effect of optogenetic stimulation of *Hoxb8* microglia

on the behavioral output. (**b, d**). High resolution (20X) images of the *Hoxb8* microglia (left panel) within mPFC from two independent brain samples obtained from *Hoxb8*$^{IRES\text{-}Cre/+}$;*ROSA26*$^{CAG\text{-}LSL\text{-}tdTomato/+}$;CX3CR1$^{GFP/+}$mice to show the morphological distinction and distribution of *Hoxb8* (left) and non-*Hoxb8* microglia at the target site of stimulation. The middle panel represents the colocalization of *Hoxb8* (left) and non-*Hoxb8* microglia. Green color represents non-*Hoxb8* microglia only population and yellow represents *Hoxb8* microglia sub-population. The right panel represents the colocalization of *Hoxb8* and non-*Hoxb8* microglia sub-population with the neuronal marker NeuN. Upper panel, 20x confocal images, scale bar 50 µm. Bottom panel, zoom portion of region of interest marked, scale bar 30 µm.

Supplementary Figure 4

Optogenetic stimulation of *Hoxb8* microglia in BLA and CeA induces anxiety behaviors as assessed on the elevated plus maze compared to controls (C57/Bl6).

(**a, b**). Representation of cannula insertion and light (473 nm) delivery into BLA (a) and CeA (b) to stimulate *Hoxb8* microglia in *Hoxb8*-$^{IRES\text{-}Cre/+}$;Ai32$^{/+}$ or non-channelrhodopsin expressing C57/Bl6 control mice. (**c, d**). Open arm time for first five minutes is significantly reduced in BLA (c) or CeA (d) implanted *Hoxb8*-$^{IRES\text{-}Cre/+}$;Ai32$^{/+}$ mice that received photostimulation at 473 nm for 5 minutes but not in C57/Bl6 mice. (**e-h**). No significant difference in the total entries to open arm or total distance covered for C57/Bl6 control mice for BLA (e, f) and CeA (g, h) implanted subjects. (**i, j**). Total entries for first five minutes are significantly lower for the BLA and CeA implanted mice within the open (Anxiogenic, i) but not in the closed arm (Anxiolytic, j). Data comparison, Paired-t test for c, d. Repeated measure One-way ANOVA with Geisser greenhouse correction and Tukey's HSD posthoc multiple comparison test for e-j. ns, $P>0.05$ not significant, *$P<0.05$,

**P<0.001. 3-4 months old mice were used for BLA and CeA implantation. ‘n’ represents total number of mice used in the experiment.

Supplementary Figure 5

Optogenetic stimulation of BLA and CeA increases freezing time in the elevated plus maze test in *Hoxb8*-Ai32 mice compared to control mice.

(**a, b**). Representation of cannula insertion and light (473 nm) delivery into BLA (a) and CeA (b) to stimulate *Hoxb8* microglia in *Hoxb8*-$^{\text{IRES-Cre/+}}$;Ai32$^{/+}$ or non-channelrhodopsin expressing C57/Bl6 control mice. (**c-l**). Significantly increased freezing time in *Hoxb8*-$^{\text{IRES-Cre/+}}$;Ai32$^{/+}$ mice (c, i) but not in C57/Bl6 mice (d, j) in response to photostimulation at 473 nm for 5 minutes within BLA (c, d) and CeA (i, j) without affecting total freezing episodes for either *Hoxb8* or control C57/Bl6 subjects within BLA (e, f) and CeA (k, l). Repeated measure One-way ANOVA with Geisser greenhouse correction and Tukey’s HSD posthoc multiple comparison test for c-l. ns, P>0.05 not significant, *P<0.05. 3-4 months old mice were used for BLA and CeA implantation. Each data point represents individual experimental subject.

Supplementary Figure 6

Optogenetic stimulation of BLA and CeA in C57/Bl6 control mice does not induce anxiety behaviors as assessed on the elevated plus maze and open field tests.

(**a, d, g, k**). Representation of cannula insertion and light (473 nm) delivery into BLA (a, g) and CeA (d, k) to stimulate non-channelrhodopsin expressing C57/Bl6 control mice. (**b, e, h, l**). Track plot (b, e) of BLA and CeA implanted *Hoxb8*-$^{\text{IRES-Cre/+}}$;Ai32$^{/+}$ mice under pre and during stimulation conditions in elevated plus maze (b, e) and open field tests (h, l) showing non-preferential

behavior of the experimental subject to the elevated plus maze platforms and open field arena in response to continuous optogenetic 473 nm laser stimulation of C57/Bl6 mice. (**c, f**). No significant change in the open (left panel, c, BLA; f, CeA) and closed arm (right panel, c, BLA; f, CeA) entry probability in the BLA and CeA stimulated condition in C57/Bl6 mice. (**i, j, m, n**). No significant change in the amount of time spent in the center (i, left, BLA, m, left CeA) and periphery (j, right BLA, n, right CeA) for BLA and CeA under continuous stimulation condition at 11.5 mW laser power in C57/Bl6 mice. ns, *P*>0.05 not significant. 3-4 months old mice were used for implantation. n=8 mice for BLA and n=6 mice for CeA for plus maze test. n=7 mice for BLA and n=7 mice for CeA for open field test.

Supplementary Figure 7

Optogenetic stimulation increases freezing time and decreases total distance traveled in open field test in *Hoxb8*-Ai32 mice but not control mice.

(**a, c, e, g**). Representation of cannula insertion and light (473 nm) delivery into BLA (a, c) and CeA (e, g) to stimulate *Hoxb8* microglia in *Hoxb8*-$^{\text{IRES-Cre/+}}$;Ai32$^{/+}$ or non-channelrhodopsin expressing C57/Bl6 control mice. (**b, d, f, h**). Increase in freezing time and significant increase in total freezing time in *Hoxb8*-$^{\text{IRES-Cre/+}}$;Ai32$^{/+}$ mice implanted within BLA (b) and CeA (f) compared to control C57/Bl6 BLA (d) and CeA (h) implanted subjects in open field test in response to photostimulation at 473 nm for 5 minutes. (**i-l**). Total activity as measured by the total distance traveled was significantly reduced in response to photostimulation of *Hoxb8* microglia in *Hoxb8*-$^{\text{IRES-Cre/+}}$;Ai32$^{/+}$ within BLA (i) and CeA (k) but not in C57/Bl6 control mice (j, BLA, l, CeA). (**m, n**). Overall decrease (however not significant) in the amount of time spent in the center zone of the open field for *Hoxb8*-$^{\text{IRES-Cre/+}}$; Ai32$^{/+}$ subjects implanted within BLA (m) and CeA (n). ns, *P*>0.05 not significant, **P*<0.05, ***P*<0.001. Repeated measure One-way ANOVA with Geisser

greenhouse correction and Tukey's HSD posthoc multiple comparison test for b-l. Paired t test for m, n. 3-4 months old mice were used for BLA and CeA implantation. Each data point represents individual experimental subject. BLA, n=7 for *Hoxb8*-Ai32, n=7 for C57/Bl6, CeA, n=7 for *Hoxb8*-Ai32, n=6 for C57/Bl6 subjects.

Supplementary Figure 8

Distribution and presence of *Hoxb8* and non-*Hoxb8* microglia within BLA and CeA following exposure to laser stimulation appear normal and indistinguishable from non-laser exposed tissue.

(**a, c**). Representation of cannula insertion and light (473 nm) delivery into BLA (a) and CeA (c) to stimulate *Hoxb8* microglia in *Hoxb8*-$^{\text{IRES-Cre/+}}$;Ai32$^{/+}$ or non-channelrhodopsin expressing C57/Bl6 control mice. (**b, d**). 20x confocal images of the *Hoxb8* (left panel) microglia from two independent brain slice samples obtained from *Hoxb8*$^{\textit{IRES-Cre/+}}$*; ROSA26*$^{\textit{CAG-LSL-tdTomato/+}}$;CX3CR1$^{\text{GFP/+}}$mice to show the morphological distinction and distribution of the *Hoxb8* (left) microglia within BLA (b) and CeA (d). The middle panel represents the colocalization of *Hoxb8* and non-*Hoxb8* microglia. Green color represents non-*Hoxb8* microglia only population and yellow represents *Hoxb8* microglia. The right panel represents the colocalization of *Hoxb8* and non-*Hoxb8* microglia with the neuronal marker NeuN. Scale bar 50 µm. Bottom panel, zoomed portion of region of interest marked, scale bar 30 µm.

Supplementary Figure 9

Optogenetic stimulation of *Hoxb8* microglia in vCA1 does not produce visible damage to *Hoxb8* microglia, reduces total distance traveled, increases % total grooming time compared to control mice (C57/B6).

(**a**). Representation of cannula insertion and light (473 nm) delivery into vCA1 region of the hippocampus to stimulate *Hoxb8* microglia in *Hoxb8*-$^{\text{IRES-Cre/+}}$;Ai32$^{/+}$ mice. (**b**). 20x confocal images from *Hoxb8*$^{\textit{IRES-Cre/+}}$*;ROSA26*$^{\textit{CAG-LSL-tdTomato/+}}$;CX3CR1$^{\text{GFP/+}}$mice brain samples showing the distribution of the *Hoxb8* microglia (left) within vCA1. The middle panel represents the colocalization of *Hoxb8* (left) and non-*Hoxb8* microglia. Green color represents non-*Hoxb8* microglia only population and yellow represents *Hoxb8* microglia. The right panel represents the colocalization of *Hoxb8*-lineage and non-*Hoxb8* microglia with the neuronal marker NeuN. Upper panel, 20x confocal images, scale bar 50 µm. Bottom panel, zoom portion of region of interest marked, scale bar 30 µm. (**c**)**.** Representation of cannula insertion and light (473 nm) delivery into vCA1 region of the hippocampus to stimulate *Hoxb8* microglia in *Hoxb8*-$^{\text{IRES-Cre/+}}$;Ai32$^{/+}$ (left panel) or non-channelrhodopsin expressing control C57/Bl6 mice (right panel). (**d, e**). Photostimulation of *Hoxb8* microglia in *Hoxb8*-$^{\text{IRES-Cre/+}}$;Ai32$^{/+}$ at 473 nm within vCA1 reduced total activity of *Hoxb8*-$^{\text{IRES-Cre/+}}$;Ai32$^{/+}$ significantly (d) without affecting the C57/Bl6 control mice (e). (**f-i**). Significant increase in percentage grooming (g) and total grooming duration (i) in vCA1 implanted *Hoxb8*-$^{\text{IRES-Cre/+}}$;Ai32$^{/+}$ (g, i) compared to vCA1 implanted C57/Bl6 mice (f, h) in response to *Hoxb8* microglia stimulation within vCA1. Repeated measure One-way ANOVA with Geiser greenhouse correction and Tukey's HSD posthoc multiple comparison test for d, e, f, h. Kurskal-Wallis test followed by Dunn's multiple comparison test for g, i. ns, $P>0.05$ not significant, *$P<0.05$. 3-4 months old mice were used for vCA1 implantation. Each data point represents individual experimental subject. n=6 for *Hoxb8*-Ai32 and n=7 for C57/Bl6 implanted subjects.

Supplementary Figure 10

Optogenetic stimulation of *Hoxb8* microglia in vCA1 induces anxiety and freezing behaviors compared to C57/B6 control mice as measured by open field tests.

(**a**). Representation of cannula insertion and light (473 nm) delivery into vCA1 region of the hippocampus to stimulate *Hoxb8* microglia in *Hoxb8*-$^{IRES\text{-}Cre/+}$;Ai32$^{/+}$ or non-channelrhodopsin expressing C57/Bl6 control mice. (**b, c**)**.** Track plots for the vCA1 implanted male C57/Bl6 (b) and *Hoxb8*-$^{IRES\text{-}Cre/+}$;Ai32$^{/+}$ mice (c) in an open field arena showing a higher preference of the experimental subject to remain in the periphery compared to anxiety-sensitive center zone in response to 6 minutes of continuous photostimulation at 2.3 mW laser power in three independent *Hoxb8*-$^{IRES\text{-}Cre/+}$;Ai32$^{/+}$ subjects but not in C57/Bl6 control mice. (**d**) Significant increase in freezing time within open field arena for *Hoxb8*-$^{IRES\text{-}Cre/+}$;Ai32$^{/+}$ compared to C57/Bl6 mice during optogenetic stimulation of vCA1 region of the hippocampus. (**e**). Significant increase in the total freezing episodes for *Hoxb8*-$^{IRES\text{-}Cre/+}$;Ai32$^{/+}$ compared to C57/Bl6 mice within open field arena during optogenetic stimulation of vCA1 region of the hippocampus at 2.3 mW laser power.

(**f, h**). Significant increase in the freezing time of *Hoxb8*-$^{IRES\text{-}Cre/+}$;Ai32$^{/+}$ compared to C57/Bl6 mice in elevated plus maze test (EPMT). (**g, i**). No significant change in the total number of freezing episodes in *Hoxb8*-$^{IRES\text{-}Cre/+}$;Ai32$^{/+}$ and C57/Bl6 vCA1 implanted subjects. Data comparison, One-way ANOVA with Tukey's HSD posthoc multiple comparison tests for d, e. Repeated measure one-way ANOVA with Geiser greenhouse correction and Tukey's HSD posthoc multiple comparison tests for f-i. ns, $P>0.05$ not significant, *$P<0.05$, **$P<0.001$, ***$P<0.0001$. Open field test, n=5 for C57/Bl6, n=6 for *Hoxb8*-Ai32, plus maze test n=5 for C57/Bl6, n=7 for *Hoxb8*-Ai32 vCA1 implanted subjects. OFT = open field test, EPMT = elevated plus maze test.

Supplementary Figure 11

Optogenetic stimulation of both microglia subpopulations using *Iba1*^{IRESCre} within DMS do not induce grooming behavior.

(**a**). Representation of cannula insertion and light (473 nm) delivery into DMS to stimulate all microglia unilaterally in *IBA1*-$^{IRES\text{-}Cre/+}$; Ai32$^{/+}$ male mice. Shown in the picture (a) is a straight cannula that was stereotaxically inserted within DMS in the right brain hemisphere at the coordinates +0.74 AP, +2.0 ML, -2.2 DV. Stimulation condition consisted of a two-minute baseline recording followed by 2 minutes of continuous optogenetic stimulation at 473 nm and 2 minutes of post stimulation recording. (**b-f**). Representation of data compiled from pre, during and post stimulation conditions showing no significant changes in the total number of grooming bouts (b-f) or the percentage of grooming time (g) in IBA1-$^{IRES\text{-}Cre/+}$;Ai32$^{/+}$ DMS implanted mice for the laser power ranges 7.0 mW (b), 7.2 mW (c), 7.4 mW (d), 7.6 mW (e) and 7.8 mW (f) for the total grooming bouts and similar power range and comparison for the percentage of time grooming (g). ns, not significant. Repeated measure One-way ANOVA with Geiser greenhouse correction and Tukey's HSD posthoc multiple comparison tests were used for the figures b-g. 3-4 months old mice were used for the cannula implantation within DMS. n=5 mice.

Supplementary Figure 12

Optogenetic stimulation of both microglia subpopulations within mPFC using *Iba1*^{IRESCre} do not induce grooming behavior.

(**a**). Representation of cannula insertion and light (473 nm) delivery into mPFC to stimulate all microglia unilaterally in *IBA1*-$^{IRES\text{-}Cre/+}$; Ai32$^{/+}$ female mice. Shown in the picture (a) is a straight cannula that was stereotaxically inserted within mPFC in the right brain hemisphere at the coordinates +1.9 AP, +0.4 ML, -2.0 DV. Stimulation condition consisted of a two-minute baseline recording followed by 2 minutes of continuous optogenetic stimulation at 473 nm and 2 minutes

of post stimulation recording. (**b**). The panels show YFP positive microglia co-stained with DAPI, a cell nuclear marker showing no visible damage following laser exposure. (**c-f**). Representation of data compiled from pre, during and post stimulation conditions showing no significant change in the total number of grooming bouts (c-e) or the percentage of grooming time (f) in IBA1-$^{IRES-Cre/+}$; Ai32$^{/+}$ mPFC implanted mice for the laser power ranges 7.0 mW (c), 7.2 mW (d) and 7.4 mW (e) for the total grooming bouts and similar power range and comparison for the percentage of time grooming (f). ns, not significant. Repeated measure One-way ANOVA with Geisser greenhouse correction and Tukey's HSD posthoc multiple comparison tests were used for the figures c-f. 3-4 months old mice were used for the cannula implantation within mPFC. n=4 mice.

Supplementary Figure 13

Optogenetic stimulation of *Hoxb8* microglia in brain slice preparations derived from the mPFC alters neighboring neuronal membrane potential and neuronal activity.

(**a**). Representation of the location of mPFC used for optogenetics in brain slice preparation in *Hoxb8*-$^{IRES-Cre/+}$;Ai32$^{/+}$ male mice (3 week old). (**b, c**). Zoomed portion of the *Hoxb8* microglia and neurons exposed to whole field optogenetic stimulation at 470 nm wavelength. Shown is a recording pipette that was positioned on a neuron nearby *Hoxb8* microglia (b) and a cartoon (c) of electrophysiological configuration. (**d, e**). Representative traces from two independent (d, neuron 1, e, neuron 2) patched neurons under pre-stimulation and during continuous (d) and during 80 Hz blue light phasic stimulation. (**f, g, h**). I-V plots showing the current-voltage relationship of a single patched neuron in the absence and presence of blue light optogenetic stimulation at various exposure times, 1ms (f), 5 ms (g) and 10 ms (h). (i-k). I-V plots showing the current voltage relationship of a single patched neuron for the steady state (i), peak (j) and tail (k) amplitudes of the neuronal response. (**l**). Representative traces from independent neurons

patched and responsive to either 80 Hz phasic (neuron 3) or continuous (neuron 4-5) blue light stimulation. Neuron 3 shows the recording of pre, during and post-stimulation conditions. Scale bar, 30 pA and 50 ms.

Supplementary Figure 14

Optogenetic stimulation of brain slice preparation derived from control mice does not alter neighboring neuronal activity

(**a**)**.** Representation of the location of mPFC used for optogenetics in brain slice preparation in C57/Bl6 male mice (3 weeks old). (**b, c**). Zoomed portion of the non-channelrhodopsin expressing *Hoxb8* microglia and neurons exposed to whole field optogenetic stimulation at 470 nm wavelength. Shown is a recording pipette that was positioned on a neuron (b) and a cartoon (c) of electrophysiological configuration. (**d, e**). Representative traces from two independent (d, neuron 1, e, neuron 2) patched neurons under pre-stimulation and during continuous blue light stimulation. Scale bar, 30 pA and 50 ms.

Supplementary Figure 15

***Hoxb8*-lineage microglia stimulation alters neuronal spike activity but not half width, T rise, T decay, Time to peak and normalized amplitude.**

As shown in Fig. 7f-h Spike frequency, instantaneous frequency and inter-event interval are significantly increased in cell attached neuronal recording in response to continuous or 80 Hz optogenetic whole field stimulation of *Hoxb8*-lineage microglia in brain slice preparation from *Hoxb8*-$^{IRES\text{-}Cre/+}$;Ai32$^{/+}$ male mice (3 week old) at 470 nm wavelength. However, no significant change was observed in the half width of the response (a), rise time (b), decay time (c), time to

peak (d) or the normalized amplitude (e). Individual data point represents the spike activity of the recorded neuron from individual traces. Data represents spike analysis over multiple traces from individual cell-attached neuronal recordings performed blindly from 3 brain samples under pre-stimulation and during optogenetic stimulation conditions (n=10 cells for continuous stimulation, n=10 cells for 80 Hz stimulation). Data comparison, Wilcoxon matched-pairs signed rank test. ns, $P>0.05$ not significant, ***$P<0.0001$.

Supplementary Figure 16

Effect of optogenetic stimulation of *Hoxb8* microglia on current-voltage (I-V) relationship.

(**a**). Representation of the location of mPFC used optogenetics in brain slice preparation in *Hoxb8*-$^{IRES\text{-}Cre/+}$;Ai32$^{/+}$ male mice (3 week old). (**b, c**). Zoomed portion of the *Hoxb8* microglia and neurons exposed to whole field optogenetic stimulation at 470 nm wavelength. Shown is a recording pipette that was positioned on a neuron nearby *Hoxb8* microglia (b) and a cartoon (c) of electrophysiological configuration. (**d, e, f**). Current responses to ramping membrane potentials (-120 mV to +70 mV (d, cell 1), -120 mV to +20 mV (e, cell 2), -60 mV to +20 mV (f, cell 3) in the absence and presence of 10 Hz blue light phasic stimulation of *Hoxb8* lineage microglia showing minimal effect of the low frequency blue light stimulation on the I-V curves of the patched neurons. (**g**). Current response to stepping (-80 mV to -40 mV) and ramping membrane potentials (-60 mV to + 20 mV) for no stimulation and 80 Hz blue light phasic stimulation of *Hoxb8* microglia. I-V curves show the effect of blue light phasic stimulation of *Hoxb8* microglia at various frequencies from 1 Hz, 5 Hz, 10 Hz and 80 Hz. Note the shift in the I-V curve to 80 Hz stimulation frequency compared to other low frequencies and no stimulation condition. (**h**). Current responses from a patched neuron to ramping membrane potentials from -80 mV to +20 mV showing the shift in the I-V curve in response to 80 Hz blue light phasic stimulation of *Hoxb8* lineage microglia.

Supplementary Figure 17

Optogenetic stimulation of *Hoxb8* in the vCA1 but not by stimulation of both populations of microglia results in increased expression of neuronal c-FOS.

(**a**). Increased neuronal expression of c-FOS is observed resulting from optogenetic stimulation of neighboring *Hoxb8* microglia in response to blue light (473 nm) stimulation of *Hoxb8IRESCre/Ai32* mice. Shown are the confocal images of *Hoxb8* microglia (green), neurons (blue) and cFOS (pink) of vCA1 region of the hippocampus in 20 μm brain sections of male mice in the absence (left image) and presence (right image) of 473 nm continuous blue light stimulation of *Hoxb8* microglia for 2-minutes (n=5 mice). White circles represent numerous distinct neuronal cells in which c-FOS expression was high and localized. (**b**). c-FOS positive neuronal cells are significantly higher in response to optogenetic stimulation of *Hoxb8* positive microglia compared to non-stimulated condition in *Hoxb8IRESCre/Ai32* mice (n=5 mice, 6 brain sections per condition). (**c**). Minimal or no detectable expression of c-FOS in neuronal cells surrounding stimulated IBA1-Cre/Ai32 mice in response to optogenetic blue light (473 nm) stimulation of both microglial subpopulations of IBA1-Cre. Shown are the confocal images of IBA1 positive microglia (green), neurons (blue) and c-FOS (pink) within vCA1 region of the hippocampus of 20 μm thick brain sections from IBA1-$^{IRES\text{-}Cre/+}$;Ai32$^{/+}$ male mice in the absence (left image) and presence (right image) of 473 nm continuous blue light stimulation of IBA1 positive (all) microglia for 2 minutes. (**d**). c-FOS activation is minimal and non-significant in response to optogenetic stimulation of IBA1 positive microglia from IBA1-Cre/Ai32 mice (n=5 mice, 6 brain sections per condition). (**e**). c-FOS activation in neuronal cells are significantly higher in response to optogenetic stimulation of *Hoxb8* positive (from *Hoxb8IRESCre/Ai32*) compared to IBA1 positive (from IBA1-$^{IRES\text{-}Cre/+}$;Ai32$^{/+}$, all microglia) microglia stimulation. Scale bar, 20 μm. Two-tailed t test with Welch's correction P<0.0001, t=12.16, df=5.000, figure b; P=0.1780, t=1.567, df=5.000 for figure d; One-way ANOVA

with Tukey's multiple comparison test for figure e. ns, not significant, *P*=0.5658, *** *P*<0.0001. Scale bar, 20 μm.

Supplementary Figure 18

Optogenetic stimulation of *Hoxb8* using *Hoxb8*^{IRESCre} or of both microglial populations using *Iba1*^{IRESCre} results in similar numbers of microglia showing increased levels of Kv1.3 potassium channel production.

(**a**). Increased expression of Kv1.3 potassium channel expression (a marker of membrane depolarization) in *Hoxb8* microglia in response to optogenetic blue light (473 nm) stimulation. Shown are the confocal images of *Hoxb8* microglia in vCA1 region of the hippocampus in 20 μm brain sections from *Hoxb8*-$^{IRES\text{-}Cre/+}$;Ai32$^{/+}$ male mice in the absence (a) and presence (b-c and of four images M1-M4) of 473 nm continuous blue light stimulation for 2 minutes. Colocalized area of Kv1.3 expression on *Hoxb8* microglia is shown in yellow color (n=5 mice). (**b**). Percentage of Kv1.3 colocalized with *Hoxb8* microglia increases significantly in response to optogenetic stimulation of *Hoxb8* microglia within vCA1. (**c**). The number of Kv1.3 foci colocalized with *Hoxb8* microglia increases significantly in response to optogenetic stimulation of *Hoxb8* microglia within vCA1. (**d**). Increased expression of Kv1.3 potassium channel expression in IBA1 positive microglia in response to optogenetic blue light (473 nm) stimulation. Shown are the confocal images of IBA1 positive microglia of vCA1 region of the hippocampus in brain slice preparation from IBA1-$^{IRES\text{-}Cre/+}$;Ai32$^{/+}$ male mice in the absence (d) and presence (e, f ~~image~~ and lower M1-M4 images) of 473 nm continuous blue light stimulation for 2 minutes. Colocalized area of Kv1.3 expression on IBA1 positive microglia is shown in yellow color. (**e**). Percentage of Kv1.3 colocalized with IBA1 microglia increases significantly in response to optogenetic stimulation of IBA1 positive microglia within vCA1. (**f**). The number of Kv1.3 foci colocalized with IBA1 microglia increases significantly in response to optogenetic stimulation of IBA1 positive microglia within vCA1. (**g**). No significant difference in % of microglia colocalized with Kv1.3 in *Hoxb8* and IBA1

microglia in the presence of optogenetic stimulation of all microglia. (**h**). The number of Kv1.3 foci colocalized with IBA1 microglia is significantly lower compared to *Hoxb8* microglia. However, the size of the Kv1.3 foci appear larger. M1-M4 in figures a, d are independent microglia from different brain sections near the cannula implanted site within vCA1. Microglia shown in –Light condition are from non-implanted brain hemisphere from vCA1. Scale bar 5 μm. Two-tailed t test with Welch's correction $P<0.0001$, t=14.01, df=5.000, figure b; $P<0.0001$, t=13.19 , df=5.000, figure c; $P=0.0005$, t=8.000, df=5.000, figure e; $P=0.0021$, t=5.855, df=5.000, figure f. One-way ANOVA with Tukey's multiple comparison test for figures g, h. ns, not significant, * $P=0.0178$; *** $P<0.0001$.

Supplementary Figure 19

Channelrhodopsin activated expression is restricted to *Hoxb8* microglia and not observed in either non-*Hoxb8* microglia or neurons.

Representative confocal 20X images of *Hoxb8*$^{\text{-IRES-Cre/+}}$-Ai32$^{/+}$ mouse brain slices labeled with IBA1, a pan microglia marker that labels all microglia (red), GFP that enhances YFP signal in *Hoxb8*-Ai32 expressing *Hoxb8* microglia (green) and neuronal marker NeuN (magenta). The images show colocalization of *Hoxb8*-Ai32 microglia with IBA1 in *Hoxb8* microglia as well as the presence of non-*Hoxb8* microglia that lack YFP signal. No Ai32 (YFP) expression was detected in neurons or non *Hoxb8* microglia. Ai32 (YFP) expression is restricted to only *Hoxb8* microglia. Scale bar 15 μm.

Supplementary Figure 20

Optogenetic stimulation of Ai32/Ai32 mice within vCA1 but in the absence of *Hoxb8^{IRESCre}* does not induce grooming and freezing behavior.

(**a**). Representation of cannula insertion and light (473 nm) delivery into vCA1 region of the hippocampus to stimulate non-Cre activated cells in Ai32/Ai32 homozygous mice (i.e. in the absence of *Hoxb8^{IRESCre}*). (**b-f**). Total grooming bouts and percentage grooming time were not significantly altered between pre-, during and post-stimulation conditions. b, c represents the data compiled from all the laser powers tested on experimental subjects. d-f represents the data compiled from laser powers 1.7, 2.0 and 2.3 mW. (**g-l**). Freezing duration and percentage of freezing representation for pre- (g, l, left), during (h, k, center) and post-stimulation (i, l, right) conditions for lower (1.7-2.0 mW, g-i) and higher (2.1-2.8 mW, j-l) laser power range. 3-4 months old mice were used for vCA1 implantation. Repeated measure one-way ANOVA with Geisser greenhouse correction and Tukey's HSD posthoc test for group comparison for b-f. 3-4 months old male mice were used for the cannula implantation within vCA1. ns, *P*>0.05 not significant. n=4 mice.

Supplementary Figure 21

Effect of optogenetic stimulation of *Hoxb8* microglia in dorsolateral (DLS) and ventromedial striatum (VMS).

(**a**). Representation of cannula insertion and light (473 nm) delivery into DLS (a) and VMS (d) to stimulate *Hoxb8* microglia unilaterally in *Hoxb8*-$^{IRES\text{-}Cre/+}$;Ai32$^{/+}$ male (a) and female (d) mice. Shown in the picture (a, d) is a straight cannula that was stereotaxically inserted within DLS (a) and VMS (d) in the right brain hemisphere at the coordinates +0.38 AP, +2.5 ML,-1.9 DV (DLS) and +0.98 AP, -1.25 ML, -3.5 DV (VMS). Stimulation condition consisted of a two-minute baseline recording followed by 2 minutes of continuous optogenetic stimulation at 473 nm and 2 minutes

of post stimulation recording. (**b, c).** No significant change in the total number of grooming bouts (b) or the percentage of grooming time (c) in response to stimulation of *Hoxb8* lineage microglia in DLS. (**e, f**). Significant increase in total grooming bouts and percentage of total time grooming is observed in response to stimulation of *Hoxb8* microglia in VMS. Stimulation condition consisted of a two-minute baseline recording followed by 2 minutes of continuous optogenetic stimulation at 473 nm and 2 minutes of post stimulation recording. (**g-n**). Representation of data compiled from pre, during and post stimulation conditions showing no significant change in the total number of grooming bouts (g-j) in DLS stimulated subjects for the laser power range 7-8.3 mW, 8-8.5 mW, 8.6-9 mW and 9.2-9.8 mW. (**k-n**). Representation of data compiled from pre, during and post stimulation conditions showing no significant change in the percentage of grooming time in VMS stimulated subjects for the laser power range 7-8.3 mW, 8-8.5 mW, and 9.2-9.8 mW, but significant increase in the percentage of grooming time in the VMS implanted subjects that received photostimulation in the power range from 8.6-9 mW. ns, not significant. Kurskal-Wallis test followed by Dunn's multiple comparison test were used for b, c, e, f, i, m. Repeated measure One-way ANOVA with Geisser greenhouse correction and Tukey's HSD posthoc multiple comparison tests were used for g, h, j, k, l, n. 3-4 months old mice were used for the cannula implantation within DLS and VMS. n=4 Male mice for DLS. n=4 female mice for VMS.

Supplementary Figure 22

Optogenetic stimulation of *Hoxb8*-lineage microglia within primary visual cortex (PVC) does not induce grooming or freezing behavior.

(**a**). Representation of cannula insertion and light (473 nm) delivery into PVC to stimulate *Hoxb8* microglia unilaterally in *Hoxb8*-$^{IRES\text{-}Cre/+}$;Ai32$^{/+}$ female mice. Shown in the picture (a) is a straight cannula that was stereotaxically inserted within PVC in the right brain hemisphere at the

coordinates -2.7 AP, +2.4 ML,-0.8 DV. Stimulation condition consisted of a two-minute baseline recording followed by 2 minutes of continuous optogenetic stimulation at 473 nm and 2 minutes of post stimulation recording. (**b, c**). No significant change in the total number of grooming bouts (b) or the percentage of grooming time (c) in response to PVC stimulation. (**d-i**). Representation of data compiled from pre, during and post stimulation conditions showing no significant change in the total number of grooming bouts (d-h) or the percentage of grooming time (i) in *Hoxb8*-$^{IRES-Cre/+}$;Ai32$^{/+}$ PVC implanted mice for the laser power ranges 7.0 mW (d), 7.2 mW (e), 7.4 mW (f), 7.6 mW (g) and 7.8 mW (h) for the total grooming bouts and similar power range and comparison for the percentage of time grooming (i). (**j-l**). Total freezing episodes (j) and percentage of freezing time (k, l) was not altered significantly in response to 2 minute continuous optogenetic stimulation. ns, not significant. Repeated measure One-way ANOVA with Geiser greenhouse correction and Tukey's HSD posthoc multiple comparison tests were used for b-l. 3-4 months old mice were used for the cannula implantation within PVC. n=4 mice.

Supplementary Figure 23

Optogenetic stimulation of *Hoxb8* microglia within retrotrapezoid nucleus (RTN) also does not induce grooming or freezing behaviors.

(**a**). Representation of cannula insertion and light (473 nm) delivery into RTN to stimulate *Hoxb8* microglia unilaterally in *Hoxb8*-$^{IRES-Cre/+}$;Ai32$^{/+}$ male mice. Shown in the picture (a) is a straight cannula that was stereotaxically inserted within RTN in the right brain hemisphere at the coordinates -6.0 AP, +0.4 ML,-1.6 DV. Stimulation condition consisted of a two-minute baseline recording followed by 2 minutes of continuous optogenetic stimulation at 473 nm and 2 minutes of post stimulation recording. (**b, c**). No significant change in the total number of grooming bouts (b) or the percentage of grooming time (c) in response to RTN stimulation. (**d-i**). Representation

of data compiled from pre, during and post stimulation conditions showing no significant change in the total number of grooming bouts (d-h) or the percentage of grooming time (i) in *Hoxb8*-$^{\text{IRES-Cre/+}}$;Ai32$^{/+}$ RTN implanted mice for the laser power ranges 7.0 mW (d), 7.2 mW (e), 7.4 mW (f), 7.6 mW (g) and 7.8 mW (h) for the total grooming bouts and similar power range and comparison for the percentage of time grooming (i). (**j-k**). Total freezing episodes (j) and percentage of freezing time (k) was not altered significantly in response to 2 minute continuous optogenetic stimulation. (**l-q**). Representation of data compiled from pre, during and post stimulation conditions showing no significant change in the total number of freezing episodes (l-p) or the percentage of freezing time (q) at laser power ranges 7.0 mW (l), 7.2 mW (m), 7.4 mW (n), 7.6 mW (o) and 7.8 mW (p) for the total freezing episodes and similar power range and comparison for the percentage of freezing time (q). ns, not significant. Repeated measure One-way ANOVA with Geiser greenhouse correction and Tukey's HSD posthoc multiple comparison tests were used for b-q. 3-4 months old mice were used for the cannula implantation within RTN. n=3 mice.

Supplementary Figure 24

Optogenetic stimulation of GFAP positive astrocytes within DMS does not induce grooming behavior.

(**a**). Representation of cannula insertion and light (473 nm) delivery into DMS to stimulate astrocytes unilaterally in *GFAP*-$^{\text{IRES-Cre/+}}$;Ai32$^{/+}$ male mice. Shown in the picture (a) is a straight cannula that was stereotaxically inserted within DMS in the right brain hemisphere at the coordinates +0.74 AP, +2.0 ML, -2.2 DV. Stimulation condition consisted of a two-minute baseline recording followed by 2 minutes of continuous optogenetic stimulation at 473 nm and 2 minutes of post stimulation recording. (**b, c).** No significant change in the total number of grooming bouts

(b) or the percentage of grooming time (c) in response to DMS stimulation. (**d-i**). Representation of data compiled from pre, during and post stimulation conditions showing no significant change in the total number of grooming bouts (b, d-h) or the percentage of grooming time (c, i) in *GFAP*-$^{IRES\text{-}Cre/+}$;Ai32$^{/+}$ DMS implanted mice for the laser power ranges 7.0 mW (d), 7.2 mW (e), 7.4 mW (f), 7.6 mW (g) and 7.8 mW (h) for the total grooming bouts and similar power range and comparison for the percentage of time grooming (i). (**j**). Representative confocal 10X images of GFAP$^{Cre/+}$-Ai32$^{/+}$ mouse brain slices labeled with GFP (green, astrocytes, left), IBA1 (red, microglia, center), a pan microglia marker that labels all microglia (red), GFP that enhances YFP signal in GFAP$^{Cre/+}$-Ai32$^{/+}$ expressing GFAP-lineage astrocytes (green) and the colocalized image (right). The images show that Ai32 is expressed in the large astrocytes relative to microglia (red). The colocalized image showed no overlap of Ai32 with IBA1 labeled microglia. Scale bar 15 µm. ns, not significant. Repeated measure One-way ANOVA with Geiser greenhouse correction and Tukey's HSD posthoc multiple comparison tests were used for the figures b-i. 3-4 months old mice were used for the cannula implantation within DMS. n=5 mice.

Supplementary Figure 25

Optogenetic stimulation of all GFAP positive astrocytes within DMS does not induce freezing behavior (i.e. anxiety).

(**a**). Representation of cannula insertion and light (473 nm) delivery into DMS to stimulate astrocytes unilaterally in *GFAP*-$^{IRES\text{-}Cre/+}$; Ai32$^{/+}$ male mice. Shown in the picture (a) is a straight cannula that was stereotaxically inserted within DMS in the right brain hemisphere at the coordinates +0.74 AP, +2.0 ML, -2.2 DV. Stimulation condition consisted of a two-minute baseline recording followed by 2 minutes of continuous optogenetic stimulation at 473 nm and 2 minutes of post stimulation recording. (**b-i**). Total freezing episodes (b) and percentage of

freezing time (c) was not altered significantly in response to 2 minute continuous optogenetic stimulation. (**d-i**). Representation of data compiled from pre, during and post stimulation conditions showing no significant change in the total number of freezing episodes (b, d-i) or the percentage of freezing time (c) at laser power ranges 7.0 mW (d), 7.2 mW (e), 7.4 mW (f), 7.6 mW (g) and 7.8 mW (h) for the total freezing episodes and similar power range and comparison for the percentage of freezing time (i). ns, not significant. Repeated measure One-way ANOVA with Geiser greenhouse correction and Tukey's HSD posthoc multiple comparison tests were used for the figures b-i. 3-4 months old mice were used for the cannula implantation within DMS.
